# Effects of Chemical Structures Interacting with Amine Oxidases on Glucose, Lipid and Hydrogen Peroxide Handling by Human Adipocytes

**DOI:** 10.3390/molecules27196224

**Published:** 2022-09-22

**Authors:** Christian Carpéné, Pénélope Viana, Zsuzsa Iffiú-Soltesz, Pál Tapolcsányi, Anna Ágota Földi, Péter Mátyus, Petra Dunkel

**Affiliations:** 1 Institut des Maladies Métaboliques et Cardiovasculaires, INSERM UMR1297, 31432 Toulouse, France; 2 Team Dinamix, Institute of Metabolic and Cardiovascular Diseases (I2MC), Paul Sabatier University, 31432 Toulouse, France; 3 Department of Organic Chemistry, Semmelweis University, H-1092 Budapest, Hungary; 4 E-Group ICT SOFTWARE, H-1027 Budapest, Hungary

**Keywords:** copper-containing amine oxidases, semicarbazide-sensitive amine oxidase, adipose tissue, hydrogen peroxide, insulin-like agents, obesity, diabetes, human adipocytes

## Abstract

Benzylamine is a natural molecule present in food and edible plants, capable of activating hexose uptake and inhibiting lipolysis in human fat cells. These effects are dependent on its oxidation by amine oxidases present in adipocytes, and on the subsequent hydrogen peroxide production, known to exhibit insulin-like actions. Virtually, other substrates interacting with such hydrogen peroxide-releasing enzymes potentially can modulate lipid accumulation in adipose tissue. Inhibition of such enzymes has also been reported to influence lipid deposition. We have therefore studied in human adipocytes the lipolytic and lipogenic activities of pharmacological entities designed to interact with amine oxidases highly expressed in this cell type: the semicarbazide-sensitive amine oxidase (SSAO also known as PrAO or VAP-1) and the monoamine oxidases (MAO). The results showed that SZV-2016 and SZV-2017 behaved as better substrates than benzylamine, releasing hydrogen peroxide once oxidized, and reproduced or even exceeded its insulin-like metabolic effects in fat cells. Additionally, several novel SSAO inhibitors, such as SZV-2007 and SZV-1398, have been evidenced and shown to inhibit benzylamine metabolic actions. Taken as a whole, our findings reinforce the list of molecules that influence the regulation of triacylglycerol assembly/breakdown, at least in vitro in human adipocytes. The novel compounds deserve deeper investigation of their mechanisms of interaction with SSAO or MAO, and constitute potential candidates for therapeutic use in obesity and diabetes.

## 1. Introduction

Many of the inhibitors of copper-containing amine oxidases described so far have been found to impair the functions of Vascular Adhesion Protein-1 (VAP-1), a membrane-bound enzyme that is peculiarly up-regulated in endothelial cells at the sites of inflammation [1,2]. Indeed, VAP-1 participates in the extravasation of leukocytes to inflamed areas [3]. Consequently, most of the patents developed for VAP-1 inhibitors are dealing with anti-inflammatory treatments, whatever the cause or the anatomical location of the inflammation [4]. The search for pharmacological inhibitors of VAP-1 [5,6], or for engineered monoclonal antibodies that may block the activity of this adhesin [7], is still under development [2], with essentially the same objective: the development of safe and potent anti-inflammatory agents.

However, many cell types other than endothelial cells also express VAP-1. Among them, the adipocytes have been known for decades to highly express a membrane-bound semicarbazide-sensitive amine oxidase (SSAO) [8], which is identical to VAP-1 [9], and is also known as primary amine oxidase (PrAO). This glycoprotein SSAO/VAP-1/PrAO has been reported to be mainly the product of the gene *AOC3*, for copper-containing amine oxidase 3. The gene *AOC3* is a member of a family that encodes amine oxidases having topaquinone as cofactor [10]. The products of this family also include diamine oxidase (encoded by *AOC1*) and the so-called retina-specific amine oxidase (encoded by *AOC2*), which are less expressed in human fat cells [11]. The enzyme encoded by *AOC3* (that is to say SSAO/VAP-1/PrAO), will be simply called SSAO throughout this study, just to emphasize that it is an adipocyte amine oxidase activity that has been explored, while not an endothelial one, and to justify the use of its historical inhibitor semicarbazide as a benchmark pharmacological blocker, although other molecules exhibit higher specificity for this *AOC3* product.

Adipocytes are well known to store excessive energy intake under the form of fat, which more precisely consists in triacylglycerols that accumulate in lipid droplets. The fat cells are also involved in immune responses and in the insulin responsiveness of the whole organism. The fat cells synthesize and secrete anti-inflammatory cytokines, such as adiponectin, and pro-inflammatory ones, such as leptin. While no change was found in the expression of the former in the fat cells from mice invalidated for *AOC3*, leptin levels were altered [12], and there was an alteration in the population of immune cells surrounding the adipocytes [13]. Noteworthy, the *AOC3* knockout mice were slightly obese but not insulin-resistant [14]. These phenotypic changes suggested that SSAO is not only involved in immunity, but also in energy balance. In fact, whatever the amine oxidase considered, the oxidation of an amine substrate generates hydrogen peroxide, which is a member of the reactive oxygen species (ROS). Among the wide variety of oxidant molecules belonging to the ROS family, hydrogen peroxide contributes at low doses to cell signaling, while at higher doses, often overpassing the antioxidant defenses, it causes cell damage. It is the former of the multiple biological effects of hydrogen peroxide [15] that supports the insulin-like effects of benzylamine, a prototypic substrate of SSAO, when applied at submillimolar doses to human adipocytes [16]. These effects consist in the activation of glucose uptake and the inhibition of lipolysis. Benzylamine also mimics several insulin effects in rodent fat cells and cultured preadipocytes, which could be qualified as adipogenic and lipogenic [17,18]. Moreover, in vivo administration of benzylamine to obese and diabetic rodents improves glucose handling [19,20]. At this point, it also must be reminded that benzylamine is present in vegetables and is one of the major alkaloids found in the tree *Moringa oleifera*, a medicinal plant reported to exert robust antiphyperglycemic and antihyperlipemic effects in rodents [21,22,23,24] and in humans [25].

From a therapeutic point of view, the insulin-like effect of benzylamine, namely its acute activation of glucose transport, suffers from two major limitations: it does not totally reach the maximal activation induced by insulin; and it does not work in skeletal muscles, the fibers of which express only small amounts of SSAO [26]. However, this SSAO substrate stimulates glucose uptake in smooth muscles [27]. Another effect of benzylamine observed in adipocytes, namely lipolysis inhibition, is instrumental to limit the accumulation of lipid intermediates in non-adipose tissues, a syndrome known as lipotoxicity. While benzylamine is considered as a gold standard substrate for SSAO by enzymologists, all of its insulin-like effects found in adipocytes can be virtually replicated by any other SSAO substrate producing hydrogen peroxide once oxidized. Moreover, such insulin-like effects have to be impaired by SSAO inhibitors. This paradigm opens new directions towards the treatment of metabolic diseases for potential novel applications of SSAO substrates and inhibitors. Thus, on the one hand, SSAO substrates active on adipocytes might limit lipid mobilization and could alleviate the risk of noxious complications of obesity and diabetes, as a consequence of insulin resistance and ectopic lipid deposition. Identifying such novel substrates is therefore of potential interest for treating cardiometabolic diseases. On the other hand, several SSAO inhibitors have been reported as limiting fatness in obese rodents [28,29], but never entered clinical anti-obesity development because they belong to the family of hydrazines, endowed with deleterious toxicological effects. This also occurred for BI 1467335, a novel AOC3 inhibitor developed by the pharmaceutical company Boehringer Ingelheim, which has been administered in patients with diabetic retinopathy or non-alcoholic steatohepatitis, but which has been discontinued for safety reasons [30]. Indeed, the search for novel and safe SSAO inhibitors might bring valuable clues for future therapeutic approaches to unhealthy overweight and associated diabetes.

To contribute to the current discovery of novel substrates and novel inhibitors of SSAO, the present study describes the effects of several molecules on various biological functions of adipocytes, such as the breakdown of triacylglycerols (lipolysis), the entry of glucose, which is the first step of its intracellular metabolism (lipogenesis), and the production of hydrogen peroxide, which can be involved in both metabolic and vascular responses of adipose tissue [31,32]. Whether the studied molecules also exhibit interaction with monoamine oxidase (MAO) is not a concern for such approach, since MAO substrates are also capable of activating glucose entry in adipocytes and in cardiomyocytes [33]. More recently, tyramine has been reported to inhibit lipolysis [34] and to activate glucose entry [35] in human adipocytes in a manner depending on MAO activity. Thus, the search for novel agents interacting with either SSAO or MAO might lead to detect candidates with beneficial effects on metabolism regulation.

One of the objectives of this work was therefore the detection of molecules interacting with SSAO or MAO in fat cells, and the selection of those producing more hydrogen peroxide than benzylamine. By consequence, we expected to identify hits that stimulate stronger than benzylamine the glucose uptake in human adipocytes. The other objective was to detect SSAO inhibitors that could upgrade the pharmacological properties of the historical inhibitor semicarbazide, or even those of more recently defined SSAO inhibitors, such as phenylallylhydrazine, also named LJP1207 [36].

## 2. Results and Discussion

### 2.1. Chemical Structures of Recognized SSAO-Interacting Agents and Novel Compounds

To extend the above-mentioned insulin-like effects of benzylamine to other potential substrates of SSAO, novel molecules were tested for their ability to regulate hydrogen peroxide production and triacylglycerol assembly/breakdown in human adipocytes. The tests were performed without ‘a priori’ with regard to the inhibitory or substrate nature of the compounds explored in parallel for their direct interplay with amine oxidase activity, and for their influence on lipogenic and lipolytic functions of human adipocytes. Accordingly, no preincubation was performed with the tested agents and all were tested after the same incubation time than the reference substrate (benzylamine) or inhibitor (semicarbazide).

**Figure 1:**
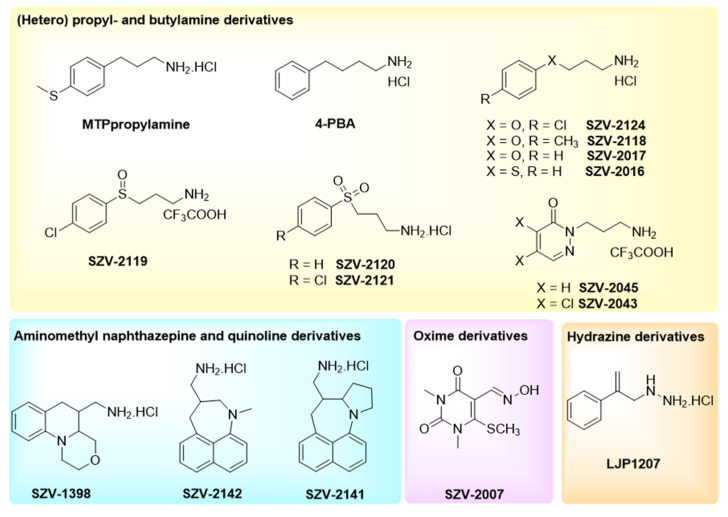
Structural formulae of the test compounds and the reference agents used in the study. 4-PBA: phenylbutylamine; LJP1207: phenylallylhydrazine.

Known SSAO substrate structures were included in the study, such as 3-(4-methylthiophenyl)propylamine (MTPpropylamine), 4-phenylbutylamine (4-PBA or phenylbutylamine) and aminomethylpyrrolidines, described as substrates for rat SSAO [37] (Figure 1). In the case of arylalkylamine substrates (as benzylamine itself), the aryl ring substitution (position, electronic effects) and modifications of the alkyl chain are critical for the effect on the enzymatic activity [37]. Thus, for the present study, a set of differently substituted arylalkylamine derivatives were selected, encompassing two pyridazine derivatives: SZV-2045 or 2-(3-aminopropyl)pyridazin-3(2*H*)-one trifluoroacetate; and SZV-2043 or 2-(3-aminopropyl)-4,5-dichloropyridazin-3(2*H*)-one trifluoroacetate (Figure 1). Replacement of one methylene of the alkyl chain with a heteroatom was also addressed. In the case of sulfur, the respective sulfide, sulfoxide and sulfone derivatives were prepared, since such modifications affect the electronic properties and the basicity of the amine [38].

To better identify the mechanism of action of SSAO in adipocytes, and to explore the potential interest of SSAO inhibition for indications other than inflammatory diseases, compounds developed at Semmelweis University as novel SSAO inhibitors [39,40] were also included in the present study. These heterocycles were obtained via an intramolecular “*tert*-amino” cyclization and functionalized with an aminomethyl moiety [39,41,42]. A typical prerequisite for the cyclization was the presence of electron-withdrawing groups, such as nitriles. From the dinitrile derivatives with a combination of a decyanation [43] and a reduction step [44], the aminomethyl derivatives could be efficiently prepared [45].

From a previously characterized library of oxime-based inhibitors [40], the oxime derivative SZV-2007 was included in the present comparative approach, while SZV-1287, which is 3-(4,5-diphenyl-1,3-oxazol-2-yl)propanal oxime was not included since already characterized as a SSAO inhibitor, and since its production was dedicated to demonstrate elsewhere that it exerts beneficial effects in diabetic states [46]. These novel molecules—exemplifying different structural types—were designed, synthesized and purified in the Department of Organic Chemistry at Semmelweis University (Budapest, Hungary) owing to a recognized expertise in the chemistry of heterorings. These compounds (thereafter named by an identifier following the prefix SZV) were tested together with SSAO-interacting hydrazine derivatives containing a reactive group binding to the topaquinone cofactor (e.g., semicarbazide, and LJP1207 or phenylallylhydrazine).

The structural formulae of these compounds are given in Figure 1, together with the chemical structures of other molecules recognized as SSAO-interacting agents, while their design and synthesis are depicted in Supplementary data (including Figures S1–S38).

### 2.2. Influence of SZV Agents on Hydrogen Peroxide Release in Human Adipose Tissue

**Figure 2:**
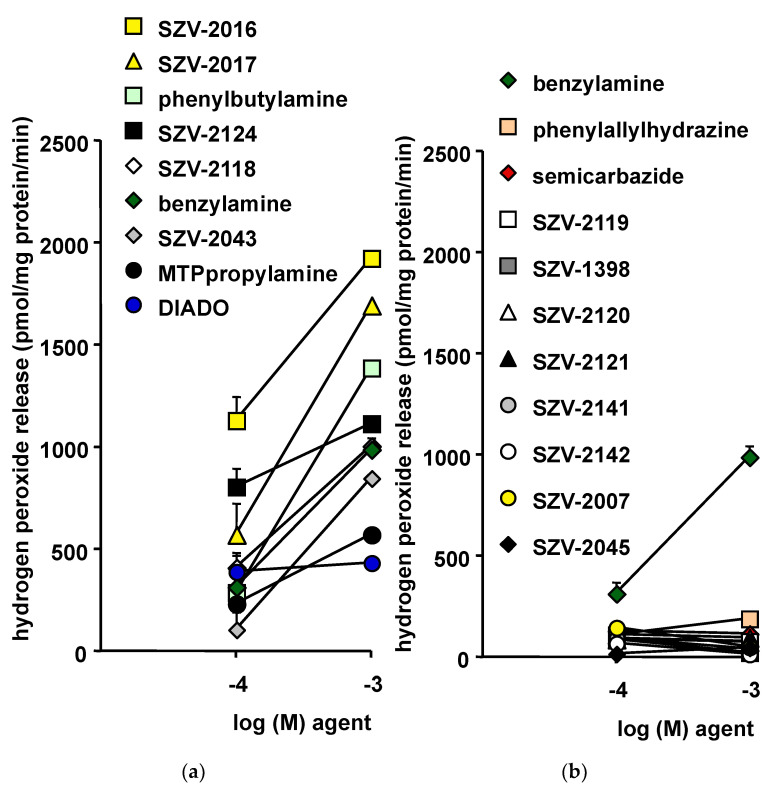
Effects of SZV molecules on the hydrogen peroxide production by homogenates of human subcutaneous adipose tissue (hAT): influence of putative SSAO substrates and inhibitors. (**a**) Benzylamine (green diamonds) is used at 0.1 and 1 mM as a reference SSAO substrate in both panels, while henylbutylamine (4-PBA, green squares) and 1,12-diaminododecane (DIADO, blue circles) are more recently characterized substrates. (**b**) Historical SSAO inhibitor is semicarbazide (red diamonds), while a more recent inhibitor is phenylallylhydrazine (LJP1207, pink squares). Mean ± SEM of 4 to 6 preparations, excepted for benzylamine, which is the mean of 12 observations. In several occurrences, error bar lies within the caption. Although not indicated for the sake of clarity, all the agents reported in panel (**a**) released hydrogen peroxide to levels significantly higher than those found without any added drug.

For comparing hydrogen peroxide production by hAT homogenates in response to the benchmark SSAO substrate benzylamine and to SZV agents, a well-established fluorometric method was used [47]. All the molecules were incubated for 30 min with hAT in identical conditions. However, for clarity, the results are presented in Figure 2 as two panels split post-hoc, based on the apparent inhibitory or substrate behavior of the agents. In this comparison, four molecules produced more hydrogen peroxide than the reference benzylamine at 0.1 and 1 mM (Figure 2a). One of them, phenylbutylamine, is known for being oxidized by SSAO [48]. The three other hits, SZV-2016, SZV-2017 and SZV-2124, could be presented potentially as “novel SSAO substrates”. In addition, SZV-2118 was almost as efficient as benzylamine itself. In addition, three molecules released a lower amount of hydrogen peroxide as did 0.1 or 1 mM benzylamine. Among them was the 1,12-diaminododecane (DIADO), which has been described as a good substrate for SSAO by the group of Di Paolo [49], but which is also an inhibitor of various polyamine oxidases [50]. Another poor activator of hydrogen peroxide production in hAT was the 3-(4-Methylthiophenyl)-propylamine (MTPpropylamine), initially characterized by the group of Unzeta [51] as a SSAO substrate.

On the other hand, eight presumed inhibitors were unable to activate hydrogen peroxide release (Figure 2b). Clearly, these SZV molecules did not reach the capacity of benzylamine in activating hydrogen peroxide release. Their lack of effect resembled that of two recognized SSAO inhibitors tested in parallel: semicarbazide and phenylallylhydrazine (Figure 2b). For most of these molecules, there was a tendency to reduce the hydrogen production by hAT homogenates when increasing their concentration from 0.1 to 1 mM. This suggested the eventuality of a spontaneous SSAO activity in hAT, functioning with undefined endogenous substrates, and which could be inhibited by SZV agents such as: SZV-2045, SZV-2007, SZV-2142, SZV-2141, SZV-2121, SZV-2120, SZV-2119 and SZV-1398.

However, SSAO is not the sole hydrogen peroxide-generating system present in fat tissue. Although the use of crude homogenates did not allow complex pathways, such as the mitochondrial respiratory chain, to be functional and to release hydrogen peroxide as a side-product of their oxidative activity, many other enzymes could have been activated or inhibited by the SZV agents, thereby altering hydrogen peroxide release by hAT independently from change in SSAO activity. MAO-A and MAO-B, which belong to the amine oxidases having flavin adenosine dinucleotide (FAD) as cofactor, are likely among such candidates, since they are highly expressed in human fat cells [52].

Only submillimolar and millimolar doses of SZV-2016, SZV-2017, SZV-2124 and SZV-2118 released more hydrogen peroxide than benzylamine when incubated with hAT. Such a requirement for relatively high concentrations can be considered at the first glance as a probe of a poorly selective or artifactual process. Indeed, this is suggesting that the tested molecules, and their reference amines, were not triggering hydrogen peroxide release via a receptor-mediated mechanism coupled to an efficient second messenger amplification, but rather via a stoechiometric release of hydrogen peroxide during oxidative deamination, whatever the AO involved.

To further investigate the putative interplay between the above-mentioned molecules and SSAO activity, several complementary tests were performed in hAT homogenates. One of these tests consisted in verifying the capacity to compete for the oxidation of ^14^C-benzylamine, demonstrated to be entirely SSAO-dependent in human fat tissue [16]. Owing to the radiometric method used, SSAO activity was determined by the amounts of radioactive aldehydes and derivatives generated as products of ^14^C-benzylamine oxidation. At this stage, it must be kept in mind that, in the chosen competition conditions, inhibition of SSAO-mediated oxidation of ^14^C-benzylamine could be obtained either with a cold substrate or with a cold inhibitor. Our approach was thereby limited to the determination of the maximal inhibition obtained with 1 mM of each SZV agent when co-incubated for 30 min with 0.5 mM ^14^C-benzylamine. The most competitive agent for ^14^C-benzylamine oxidation was phenylallylhydrazine, leading to total inhibition. This was in agreement with the established SSAO-blocking properties of this agent, initially called LJP-1207 [36]. Alongside this best hit, the decreasing rank order of maximal competition was: SZV-2016 followed by SZV-2017, MTPpropylamine, phenylbutylamine and SZV-2043 (leading to 85.6, 79.8, 67.5, 66.2 and 61.3% inhibition, respectively; n = 3, not shown). All these agents were previously supposed to be SSAO substrates, since they increased hydrogen peroxide release in hAT as shown above. Among the presumed SSAO inhibitors, the best hits for inhibition of ^14^C-benzylamine oxidation were SZV-2142, SZV-2007 and SZV-2045 (leading to 60.0, 59.7 and 47.8% inhibition, respectively). Finally, SZV-2119, SZV-2120 and SZV-2121 were very poor competitors (not shown), at least when tested at 1 mM without preincubation.

To better discriminate the supposed SSAO inhibitors from the substrates and to determine whether inhibitors of ^14^C-benzylamine oxidation were competitive or not, irreversible, or not, numerous other expensive experiments would have been necessary (requiring preincubation, washing, increasing doses). Such complete pharmacological characterization was out of the scope of the current study, which aimed to describe the biological effects resulting from changes in the activity of the so-called ‘ectopic SSAO’ abundant in adipocytes [53]. Thus, considering the paucity of available biological resource and of radioactive material, another approach was developed. Indeed, it was the capacity of the supposed inhibitors to impair the benzylamine-induced production of hydrogen peroxide that was measured with the fluorometric method used above, since it differentiates SSAO inhibitory from SSAO substrate properties.

### 2.3. Inhibition by SZV Agents of the Benzylamine-Induced Hydrogen Peroxide Release in Human Adipose Tissue

**Figure 3:**
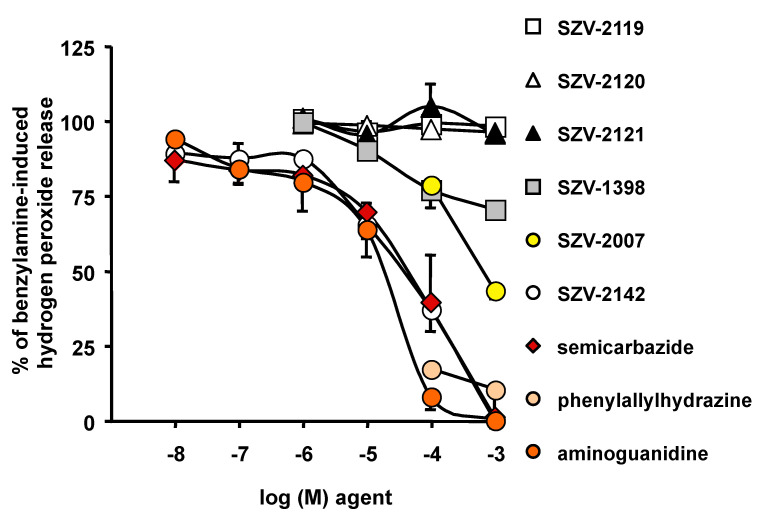
Inhibition by SZV agents and recognized SSAO inhibitors of the benzylamine oxidation by homogenates of human subcutaneous fat depots. The oxidation of 1 mM benzylamine was set at 100% without any competitor. Each agent was added at the indicated final concentration at the same time as the benzylamine and the fluorogenic mixture, then incubation lasted 30 min at 37 °C. Each point is the mean +/− SEM of six preparations, except for SZV-2119, SZV-2120 and SZV-2121, for which n = 3.

The capacity to inhibit the oxidation of 1 mM benzylamine was tested in hAT preparations essentially for the molecules that were unable to activate hydrogen peroxide (see Figure 2b). In parallel, we used recognized SSAO inhibitors: semicarbazide, aminoguanidine and phenylallylhydrazine. Only several SZV agents inhibited benzylamine-induced hydrogen peroxide release: especially SZV-2142, then SZV-2007 and SZV-1398 (Figure 3). Similar to that found with the radiometric method, a millimolar dose of SZV-2007 impaired approximately 50% of the SSAO activity found in hAT. In contrast, three other molecules (SZV-2119, SZV-2120 and SZV-2121) did not exhibit any notable SSAO inhibitory property (Figure 3). Since these three compounds were not stimulating hydrogen peroxide, their putative functional interplay with SSAO, either as substrate or blocker, was discarded.

Taken together, the two verification steps (competition for ^14^C-benzylamine oxidation and inhibition of benzylamine-induced hydrogen peroxide release) converged in indicating that SZV-2007 and SZV-2142 behaved as notable SSAO inhibitors. However, their apparent inhibitory capacity was lower than that of phenylallylhydrazine. Another limit of our complementary studies on inhibition of copper-containing amine oxidases, is that we did not consider whether the various lysyl oxidases [54] potentially present in hAT play a relevant role in oxidizing benzylamine.

### 2.4. Semicarbazide-Sensitive Activation of Hydrogen Peroxide Release by SZV Agents

**Figure 4:**
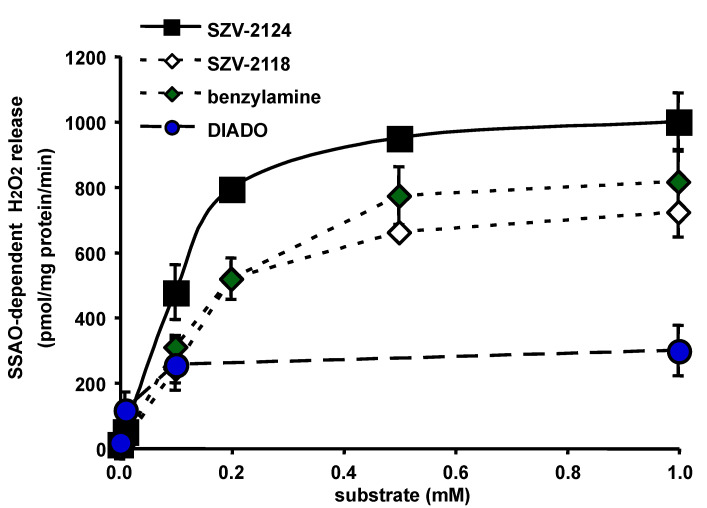
Activation of amine oxidase in human adipose tissue preparations by increasing concentrations of benchmark and novel substrates. The oxidation of benzylamine (green diamonds), the benchmark substrate for human SSAO/VAP-1 was determined in a total of 15 hAT homogenates. SSAO activity (difference between substrate-induced hydrogen peroxide release in the absence of any inhibitor and the residual signal obtained in the presence of 1 mM semicarbazide) was measured in parallel conditions for DIADO (blue circles), SZV-2118 (open diamonds) and SZV-2124 (black squares). Each value is the mean of at least two independent experiments or is the mean ± SEM of at least five determinations when error bars are visible.

It was then investigated whether the substantial increase of hydrogen peroxide production by hAT in response to presumed SSAO substrates was really related to SSAO activity. Since SZV-2124 and SZV-2118 generated at 0.1 mM more hydrogen peroxide production than benzylamine (see Figure 2a), a wider range of increasing concentrations was studied (0.01–1 mM) to determine the K_m_ and V_max_ for their SSAO-dependent oxidation, i.e., sensitive to inhibition by 1 mM semicarbazide. Michaelis-Menten hyperbolic curves were obtained with up to six different concentrations of substrate (Figure 4). Double reciprocal plot analysis of hAT SSAO activity resulted in mean K_m_ values of 49 and 36 µM, with V_max_ values of 1084, and 712 pmoles of hydrogen peroxide released/mg protein/min for SZV-2124 and SZV-2118, respectively (n = 2–5). For benzylamine, K_m_ was 41 µM and V_max_ was 693 pmol/mg/min. These results suggested that SZV-2124 and SZV-2118 were slightly better SSAO substrates than benzylamine. Moreover, semicarbazide inhibited more than 95% of the oxidation of all these amines, and there was no detectable inhibition of the hydrogen peroxide produced by these agents in the presence of 0.1 mM pargyline, an observation indicating that SZV 2118 and SZV 2124 were practically devoid of MAO substrate activity. Enzyme kinetics also showed that DIADO was a high affinity substrate (K_m_ of 10 µM) but with unexplained limited maximal velocity of metabolism by SSAO in hAT (V_max_ of 337 pmol/ mg protein/min) (Figure 4). Our observation contrasted with the conclusion of the original description of DIADO, characterizing it as a molecule exhibiting a higher catalytic efficiency than the prototypic substrate benzylamine [49]. Such a discrepancy was probably due to the fact that the crude preparation of hAT used here was mixing lipids and proteins, and thereby altered the bioavailability of a somewhat lipophilic substrate, which was probably trapped in the lipids of disrupted fat droplets. Alternatively, DIADO, which has also been reported to be a competitive inhibitor for a FAD-containing amine oxidase, the acetylpolyamine oxidase (PAOX) [50], did not behave simply as a SSAO substrate in hAT. While the use of a more purified enzyme source is highly recommended for accurate kinetics, the use of crude hAT homogenates or isolated adipocytes has the advantage of tracking the behavior of the tested molecules under ‘physiological’ conditions. In such an environment, the substrate availability, the presence of allosteric regulators, or even the pH environment, are not optimal for SSAO activity, but remain relevant for potential in vivo applications. Moreover, the direct study of the human form of SSAO, even not under a purified form, avoided the limitations due to interspecies differences in substrate and inhibitor selectivity when compared to the rodent form, as recently documented [6].

Unfortunately, the oxidation of low doses of two other presumed substrates, SZV-2016 and SZV-2017, could not be achieved successfully in parallel due to a device failure. Nevertheless, it could be established that, for 1 mM SZV-2016 and SZV-2017, 99.7 ± 0.4% and 93.2 ± 2.6% of the oxidation was sensitive to 1 mM semicarbazide, respectively. Although incomplete, these findings provided evidence for their SSAO substrate property.

All these complementary analyses indicated the presence of “good” substrates and “novel” inhibitors for SSAO among the tested SZV agents. While SSAO, which supports benzylamine oxidation in hAT, has to be considered as a major target of these SZV agents, it could not be excluded that these molecules were also interacting with other AOs expressed in hAT. Nevertheless, our working hypothesis simplified the activities of SSAO and MAO to hydrogen peroxide-generating systems. They were supposed to modulate redox-sensitive signaling targets within the fat cell, and thereby to influence responses that are sensitive to insulin, i.e., the stimulation of hexose uptake or the inhibition of triacylglycerol breakdown. The following experiments were aimed at exploring this issue.

### 2.5. Modulation of Glucose Transport in Human Adipocytes by SSAO-Interacting Agents

To investigate a potential therapeutic interest of SSAO-interacting agents other than their promising anti-inflammatory properties (as reviewed in: [5,55]), it was important to examine cell models expressing SSAO activity other than endothelial cells, which loose SSAO expression once cultured. Thus, the influence of SZV agents and suitable controls was investigated on the assembly/breakdown of triacylglycerols in freshly isolated adipocytes, i.e., on functions commonly known as lipogenesis and lipolysis.

**Figure 5:**
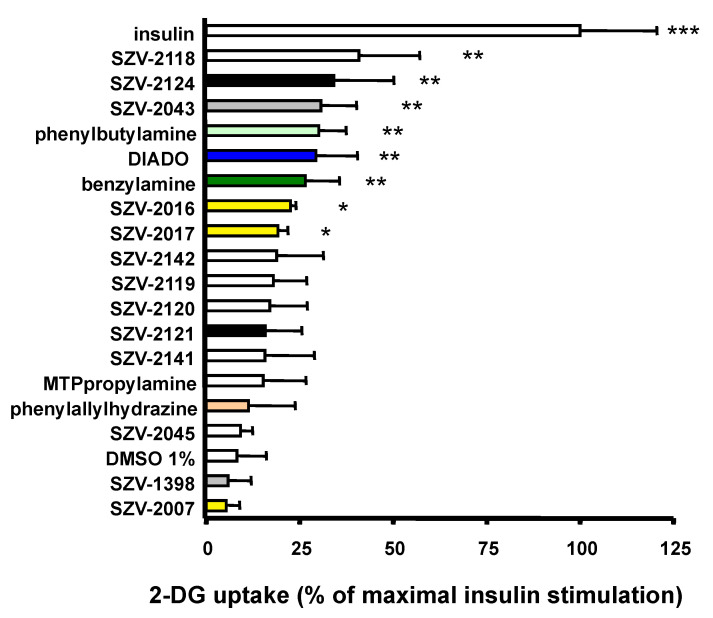
Stimulation of ^3^H-2-deoxyglucose (2-DG) uptake into human adipocytes by 0.1 mM of SZV agents and SSAO-interacting agents. Data are expressed as percentage of insulin-stimulated 2-DG uptake, with 100 nM insulin set at 100% (open top bar) and basal at 0% in each adipocyte preparation. All the pharmacological agents were tested at 0.1 mM, save DMSO at 1% (*v*/*v*) (used as vehicle for SZV-2007, -2141 and -2142), then ranked according to decreasing insulin-like effect. Each bar is mean ± SEM of 5–8 adipocyte preparations. Different from basal uptake at: * *p* < 0.05, ** *p* < 0.01, *** *p* < 0.001.

The hexose uptake, which is the first step of de novo lipogenesis, was determined in human adipocytes and was expressed as a percentage of the maximal response to 100 nM insulin. The SZV agents were tested at 0.1 mM together with negative and positive controls. The stimulation by benzylamine of the transport of a nonmetabolizable glucose analogue was reproduced by various SZV agents (Figure 5). Alongside phenylbutylamine and DIADO, SZV-2118, SZV-2124 and SZV-2043 appeared to surpass the stimulatory effect of benzylamine on the uptake of ^3^H-2-deoxyglucose (2-DG). At 0.1 mM, SZV-2016 and SZV-2017 also reproduced partly the insulin activation of hexose uptake to an extent that was similar to that obtained with benzylamine. SZV-2142 induced a variable response, the intermediate nature of which could not be determined. In contrast, SZV-2007, SZV-1398, SZV-2045 and phenylallylhydrazine, already shown to be inactive on hydrogen peroxide production, did not activate glucose entry (Figure 5). Lastly, DMSO, which was used at 1% final as vehicle for several agents (SZV-2007, SZV-2141 and SZV-2142), was without noticeable influence of hexose uptake.

As with hydrogen peroxide release, it therefore appeared that the best substrates were significantly active at 0.1 mM on 2-DG uptake, while inhibitors were inefficient. However, there was not a clear-cut frontier between these two categories and many molecules exhibited intermediate responses. Of note, among the two pyridazinone derivatives, SZV-2043 and SZV-2045, only the former, which releases hydrogen peroxide in hAT, was activatory. The lack of hydrogen peroxide-releasing and of 2-DG uptake activating properties of SZV-2045—being a 2-(3-aminopropyl)-3(2*H*)-pyridazinone—was consistent with the fact that various pyridazinone derivatives have been described as inhibitors for human SSAO/VAP-1 [56], and for MAO-B as well [57].

Thus, it was examined whether a relationship exists between the capacities to release hydrogen peroxide and to stimulate hexose uptake in human subcutaneous adipocytes.

### 2.6. Relationship between the Ability to Generate Hydrogen Peroxide and to Activate Hexose Uptake in Adipocytes

**Figure 6:**
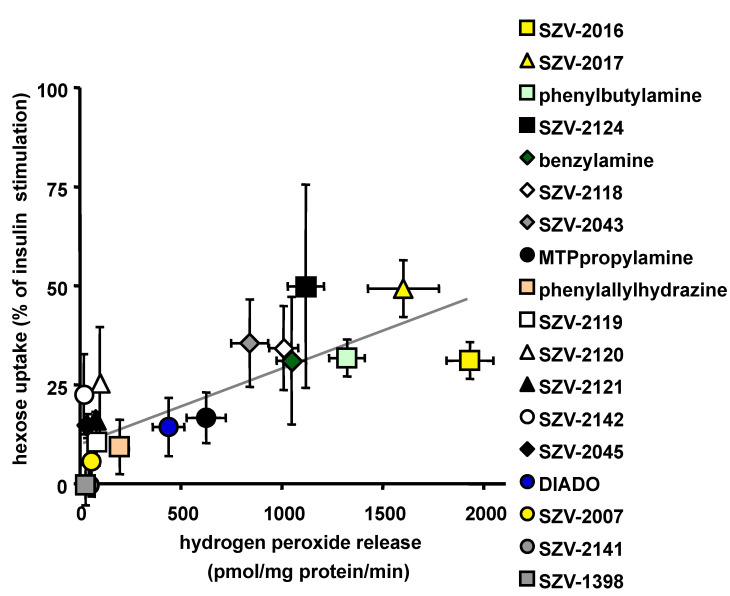
Scatter plot of the activation of hexose uptake in human adipocytes induced by SZV molecules and reference agents as a function of their abilities to generate hydrogen peroxide in adipose tissue. In the legend, the molecules tested at 1 mM are ordered according to the decreasing rank order of the hydrogen peroxide release they provoked in adipose tissue, as reported in Figure 1. Each point is the mean ± SEM of four to nine determinations. The Pearson correlation coefficient of the regression line is: r = 0.736, significant at *p* < 0.01.

A relationship appeared clearly when plotting the activation of hydrogen peroxide release vs the activation of glucose uptake in response to 1 mM of each of the tested SZV agents and their control. Figure 6 shows that most of the presumed SSAO inhibitors were inefficient when tested alone, while the SSAO substrates increased both hydrogen peroxide production and 2-DG uptake. When considering all the tested agents, including fourteen SZV molecules and four reference drugs, a linear relationship could be established between hydrogen peroxide release and hexose uptake (with Pearson correlation coefficient r = 0. 736, *p* < 0.0001) (Figure 6). However, the scatter plot showed that not all the agents tightly followed the apparent linear relationship estimated by regression analysis. Among the most noticeable outliers, the supposed inhibitors SZV-2142 and SZV-2120 reproduced up to 25% of the maximal effect of insulin on glucose entry without producing detectable amounts of hydrogen peroxide (Figure 6). Nevertheless, at the left bottom part of the estimated regression line, the inhibitors SZV-1398 and SZV-2141 were unable to release hydrogen peroxide and to promote hexose uptake.

The relationship between the capacity to generate hydrogen peroxide in hAT and for that to activate glucose uptake in adipocytes, although significantly estimated by a linear correlation, could be more complex since it appeared that a plateau was reached in the capacity to activate glucose transport. It therefore seemed that a limiting factor hampered the amines to totally reproduce the insulin stimulatory effect.

However, focusing on the most active SSAO substrates, there were five molecules that acted similarly to—or even better than—benzylamine in promoting both hydrogen peroxide release and hexose uptake: SZV-2016, SZV-2017, SZV-2043, SZV-2118 and phenylbutylamine. A sixth molecule, namely SZV-2124, exhibited a large variability in stimulating 2-DG uptake, but reproduced almost 50% of the insulin stimulating effect, as did SZV-2017. These two molecules were among the most efficient hydrogen peroxide releasers in hAT homogenates, thereby confirming our working hypothesis: like benzylamine, any other amine oxidase substrate that is readily oxidized in adipocytes can acutely activate glucose utilization, at least in vitro.

### 2.7. Inhibition of Benzylamine-Induced Glucose Uptake by SSAO-Interacting Molecules

**Figure 7:**
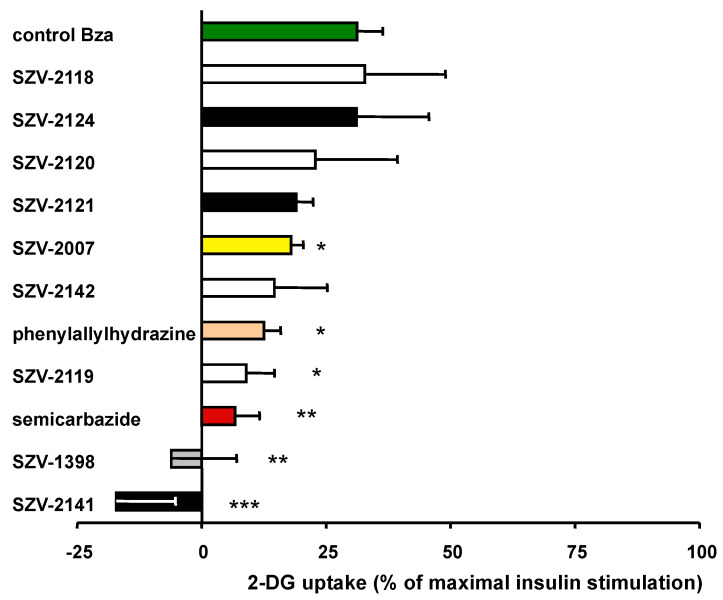
Stimulation of glucose transport in human adipocytes by benzylamine is sensitive to SZV inhibitors. Human adipocytes were incubated 45 min without (basal, set at 0%), with 100 nM insulin (100% reference), or with 1 mM benzylamine alone (control Bza, green bar) or combined with 1 mM of SZV agents or reference SSAO inhibitors. 2-DG uptake was then assayed on over a 10 min period and expressed as in Figure 6. Mean ± SEM of four to six adipocyte preparations. Different from benzylamine alone at: * *p* < 0.05; ** *p* < 0.01; *** *p* < 0.001.

It was then investigated whether the presumed inhibitors were impairing the insulin-like effect of benzylamine on glucose entry. In six independent human adipocyte preparations, 1 mM benzylamine induced 31.1 ± 5.2% of the maximal glucose transport (Figure 7), therefore confirming our previous observations [16]. This ‘partial insulin-like effect’ was significantly impaired by 1 mM of semicarbazide or phenylallylhydrazine (Figure 7). SZV-1398 and SZV-2141 abolished hexose uptake, but strikingly below the basal levels, suggesting that, in addition to their SSAO inhibitory component, they were interfering with another step of hexose uptake assay. Additionally surprising was the inhibitory behavior of SZV-2119, which was discarded as notable SSAO inhibitor since it did not inhibit benzylamine-induced hydrogen peroxide production (see Figure 3). In contrast, SZV-2007, able to inhibit the SSAO-mediated oxidation of benzylamine in hAT homogenates, impaired moderately the benzylamine stimulation of glucose transport (Figure 7). In a more consistent manner, SZV-2120 and SZV-2121, found to be inactive on benzylamine-induced hydrogen peroxide release, were unable to alter its insulin-like effect on glucose transport. Lastly, the substrates SZV-2118 and SZV-2124 did not impair the benzylamine effect. However, their own effect was not clearly additive to that of benzylamine, suggesting that a limiting step common to all these amine oxidase substrates prevented to activate 2-DG uptake as strongly than insulin.

Although many of these discrepant observations deserved additional verifications, it was investigated whether the SZV molecules could modulate the lipolytic activity of human adipocytes.

### 2.8. Inhibition of Lipolysis as Another Insulin-like Effect of Novel SSAO Substrates Exerted on Human Adipocytes

Since insulin is antilipolytic, and based on the already described antilipolytic effects of benzylamine [16], the antilipolytic property of the SZV molecules was studied with the same comparative approach. As with glucose transport assays, the use of functional, freshly isolated, human adipocytes is required to explore their regulation of triacylglycerol breakdown. It is recognized that the use of a lipolytic agent enhancing moderately the lipolytic process is a prerequisite for suitable detection of antilipolytic responses. It was therefore investigated whether 0.1 or 1 mM of each of the tested SZV molecules was capable of inhibiting the glycerol release stimulated by 10 nM isoprenaline. The submaximal stimulation of lipolysis by 10 nM isoprenaline increased basal glycerol release by a factor equivalent to 2.9 ± 0.2 times. This allowed the detection of any further increase or inhibition in response to a lipolytic or an antilipolytic factor, respectively. The mild lipolytic effect of 10 nM isoprenaline was used as reference and was set at 100%. Therefore, any significant decrease of this percentage traduced an antilipolytic response. This was the case for benzylamine since only 40.5 ± 10.6% and 31.6 ± 7.9% of the 10 nM isoprenaline-induced lipolysis was resistant to 0.1 and 1 mM of the prototypic SSAO substrate, respectively (n = 12, *p* < 0.01). In the same conditions, more than 66.3 ± 6.1% of isoprenaline-induced lipolysis resisted to 100 nM insulin (n = 8, *p* < 0.05). Even if the antilipolytic effect of benzylamine was greater than that of insulin, it was expected that novel substrates could reproduce or improve such insulin-like effect.

**Figure 8:**
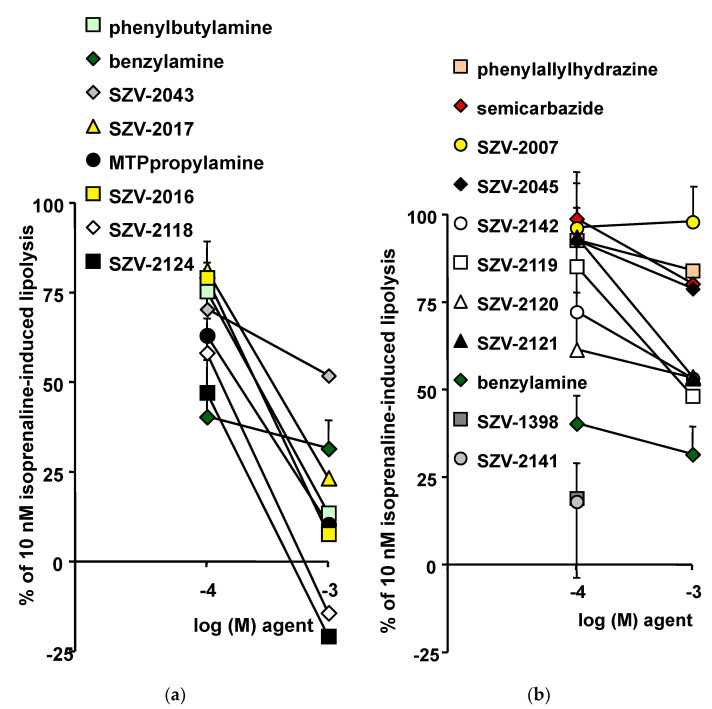
Comparison of the inhibitory effects of SZV agents on isoprenaline-induced lipolysis in human subcutaneous adipose cells. In each adipocyte preparation, the submaximal stimulation of glycerol release by 10 nM isoprenaline was set at 100%, while basal (equivalent to 0.23 ± 0.05 µmol/100 mg lipids/90 min) was set at 0%. The remaining lipolytic effect of isoprenaline in the presence of the indicated concentrations of supposed or recognized SSAO substrates (**a**) or inhibitors (**b**) was expressed as percentage of 10 nM isoprenaline-induced lipolysis. In several conditions, this percentage was >100% or <0%. Mean ± SEM of five to eight determinations.

In fact, 1 mM of the supposed (SZV-2016, SZV-2017), or recognized (phenylbutylamine, MTPpropylamine) SSAO substrates elicited a stronger antilipolytic response than benzylamine (Figure 8a). Only SZV 2043 impaired isoprenaline-induced lipolysis to a lesser extent than benzylamine. Two compounds exhibited an unexpected behavior, SZV-2118 and SZV-2124, since they totally abolished lipolysis, and even reduced glycerol release to levels lower than baseline (Figure 8a).

In contrast, most of the inhibitors were much less efficient than benzylamine in inhibiting lipolysis (Figure 8b). Indeed, for SSAO inhibitors, there was no rationale for activation or inhibition of isoprenaline-induced lipolysis. Accordingly, SZV-2007 and SZV-2045, as well as phenylallylhydrazine and semicarbazide, did not impair significantly the isoprenaline lipolytic action (Figure 8b). Again, there was an exception to the rule, since SZV-1398 and SZV-2141were potent antilipolytic agents at 0.1 mM. This was likely related to the colored solution they generated, which hampered the colorimetric detection of glycerol release, and even prevented an accurate determination of their effects at 1 mM. One of the limitations of the present study is that the properties of SZV-1398 and SZV-2141 could not be entirely characterized as a consequence of their colored solution at 1 mM, which could not be compensated.

### 2.9. Relationship between the Ability to Generate Hydrogen Peroxide and to Inhibit Lipolysis in Adipocytes

**Figure 9:**
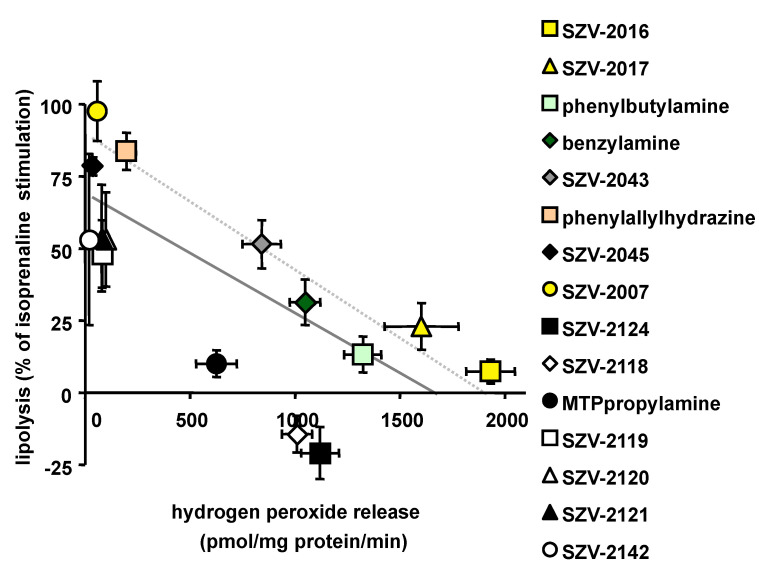
Correlation analysis between hydrogen peroxide release in hAT homogenates and antilipolytic effect in adipocytes for 1 mM of SZV molecules and reference agents. The X axis correspond to the amine-induced hydrogen peroxide release as expressed in Figure 2, while the Y axis correspond to the inhibition of 10 nM-induced lipolysis, as expressed in Figure 8. The plain line represents the linear regression estimating the relationship between the two responses to all tested agents (with equation being y = 0.039x + 64.5, and Pearson correlation coefficient r = 0.727, *p* < 0.02). The linear regression line for the substrates only (coloured points in the scatter plot) is represented by a dotted line, characterized by: y = 0.046x + 88.8, with r = 0.961, *p* < 0.001). Each point is the mean ± SEM of four to eight observations. In several occurrences, x-error bar lies within the caption.

Then, it was investigated whether a relationship exists between the capacity of SZV agents to promote hydrogen peroxide release in hAT and their capacity to impair isoprenaline-induced lipolysis. The scatter plot shown in Figure 9 indicates that a linear regression could be established between these two responses, irrespective of the substrate or inhibitor nature of the tested molecules. However, the relationship appeared less tight than in the case of glucose transport. Linkage appeared more evident when considering only a subset of substrates (displayed as a dotted line in Figure 9). Again, SZV-2016 and SZV-2017 behaved as substrates that reproduce the effects of benzylamine, or even exceed it. In other words, for SZV-2043, benzylamine, phenylbutylamine, SZV-2017 and SZV-2016, the greater the hydrogen peroxide production, the stronger was the antilipolytic response.

Among the agents excluded from such tight relationship, were those able to moderately inhibit lipolysis, but which were unable to generate hydrogen peroxide: SZV-1398, SZV-2141, SZV-2142, SZV-2119, SZV-2120, SZV-2121 and SZV-2045. Moreover, there was also three molecules that inhibited lipolysis to apparently a larger extent than presumed by the regression built with the SSAO substrates: MTPpropylamine, and more especially SZV-2124 and SZV-2118. Since the two latter molecules were the strongest antilipolytic agents found in this study, one could suppose that toxic events other than SSAO activation were involved in their inhibitory effects.

### 2.10. Verifications about SSAO Contribution to the Antilipolytic Effects of SZV Agents

Additional investigations were performed in human adipocytes to decipher the causes of the unexpectedly strong antilipolytic responses to several SZV agents. First, it must be mentioned that, at 0.1 mM, SZV-2118 and SZV-2124 were less antilipolytic than 0.1 mM benzylamine. This also applied for MTPpropylamine (see Figure 8a), suggesting that, when used at 1 mM, these compounds impacted targets other than SSAO. Then, it was observed that MTPpropylamine on its own did not alter basal lipolysis (0.28 ± 0.09 vs 0.28 ± 0.07 µmol glycerol/100 mg lipids/90 min, n = 5, NS), as it was the case for benzylamine (0.22 ± 0.04 µmol glycerol/100 mg lipids/90 min, n = 5, NS). In contrast, 1 mM of SZV-2118 and SZV-2124 inhibited basal lipolysis (both lowering to 0.14 ± 0.04 µmol glycerol/100 mg lipids/90 min, n = 5, *p* < 0.05). Generally, inhibition of basal lipolysis is a phenomenon observed only when the baseline is elevated by in vivo stressing conditions (prolonged fasting, cold exposure), which did not apply in our study, or when adipocytes are subjected to cytotoxic agents. The extreme antilipolytic effect of high doses of SZV-2118 or SZV-2124 was therefore considered doubtful. Such supposed toxicity of SZV-2124 relies with its above-reported stimulatory effect on 2-DG uptake: exhibiting a higher variability at 1 mM (see Figure 6), while at 0.1 mM, it was among the most efficient insulin-like agents (see Figure 5).

**Figure 10:**
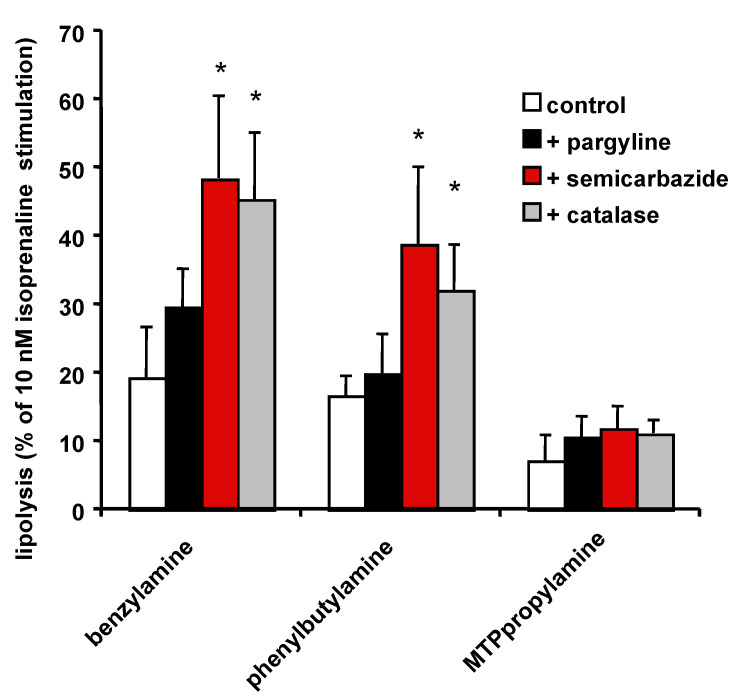
Inhibition of the antilipolytic effect of SSAO substrates of by amine oxidase inhibitors and catalase. The antilipolytic effect of 1 mM benzylamine, phenylbutylamine and methylthiophenylpropylamine was expressed as percentage of 10 nM isoprenaline-induced lipolysis, without inhibitor (control, open columns), or with 1 mM pargyline (black columns), 1 mM semicarbazide (red columns) or 5000 IU/mL catalase (grey columns). Mean ± SEM of seven determinations. Significantly different from respective control at: * *p* < 0.05.

Finally, experiments aiming at blocking the effect of SZV antilipolytic agents confirmed that semicarbazide and catalase significantly impaired the benzylamine antilipolytic effect (Figure 10), as previously reported [16]. Such a blockade of SSAO activity or hydrogen peroxide scavenging also impaired the antilipolytic effect of phenylbutylamine, but surprisingly not that of MTPpropylamine (Figure 10). Thus, MTPpropylamine antilipolytic effect was not predominantly mediated by the hydrogen peroxide generated by SSAO activation.

Since MTPpropylamine is an aromatic amine that might interact with adrenergic receptors (ARs), additional experiments aimed at verify whether its impairment of isoprenaline-induced lipolysis was due to impairment of the lipolytic β-ARs or to activation of the antilipolytic α_2_-ARs present in human fat cells. Table 1 summarizes these observations and indicates that MTPpropylamine was able to inhibit the lipolytic effect of forskolin, which is independent from β-AR activation, and was also inhibiting isoprenaline effect even in the presence of the α_2_-AR antagonist methoxyidazoxan (Table 1). A mediation by AR inhibition or activation was therefore discarded. Nevertheless, the present study could not decipher the mechanisms involved in the strong antilipolytic effect of MTPpropylamine, which alongside its SSAO substrate property, also shares MAO-B substrate and MAO-A inhibitor abilities [51]. In the case of benzylamine and phenylbutylamine, the antilipolytic action was not only a consequence of oxidation by SSAO, since a residual antilipolytic effect was detectable in the presence of semicarbazide. However, this SSAO-independent mechanism was not related to an interplay with α_2_-ARs or β-ARs (Table 1).

**Figure 11:**
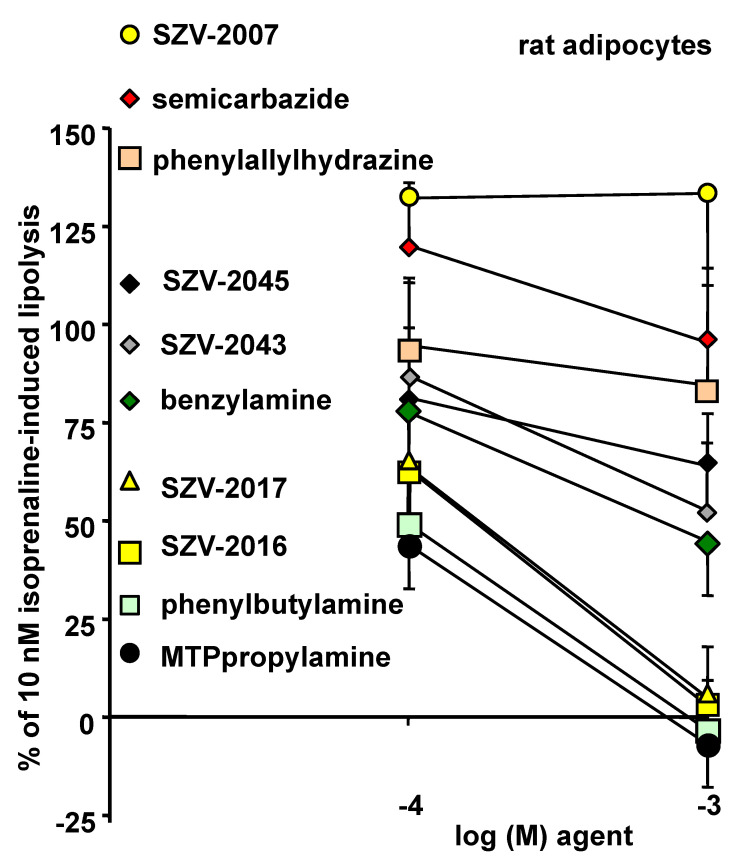
Antilipolytic effects of SZV molecules and SSAO-interacting benchmark agents in rat adipocytes. In each adipocyte preparation, the submaximal stimulation of glycerol release by 10 nM isoprenaline is set at 100%, while basal is set at 0%. Negative percentages indicate lipolysis inhibition below baseline by millimolar concentrations of SSAO substrates (SZV-2016, SZV-2017, phenylbutylamine and methylthiophenylpropylamine). Percentages higher than 100% indicate a tendency to improve the lipolytic effect of 10 nM isoprenaline, which resulted non-significant after ANOVA and post-hoc tests for the inhibitors SZV-2007, semicarbazide or phenylallylhydrazine. For clarity, only one superior or inferior error bar is shown for each point, being the mean of eight determinations.

To discard a putative mediation of the MTPpropylamine effect via an activation of the antilipolytic α_2_-ARs, ancillary experiments were performed in rat adipocytes. In this animal species, the α_2_-AR agonists are poorly antilipolytic [29]. Regardless, MTPpropylamine totally abolished isoprenaline-induced lipolysis in rat adipocytes (Figure 11). These additional explorations also confirmed that inhibitors behaved differently from amine oxidase substrates. Like in human adipocytes, three subgroups of molecules could be evidenced. In the first one, the inhibitors SZV-2007, semicarbazide and phenylallylhydrazine were unable to exhibit antilipolytic effects. An intermediate subgroup of molecules consisted in SZV-2045, SZV-2043 and benzylamine, which limited moderately the lipolytic activity. A third group of strongly antilipolytic agents encompassed the novel substrates SZV-2016 and SZV-2017, together with the known SSAO activators phenylbutylamine and MTPpropylamine. Although it could not be verified whether the strong antilipolytic effect of these latter compounds was solely dependent on amine oxidation, the use of this animal model confirmed our observations made in human adipocytes and reinforced our proposed hypothesis linking amine oxidation, generation of hydrogen peroxide and induction of antilipolytic effects.

## 3. Materials and Methods

### 3.1. Chemistry

The SZV compounds were prepared at the Department of Organic Chemistry, Semmelweis University (Budapest, Hungary), according to [40]. The synthetic procedures and characterization of the studied compounds are provided in the supporting information (see Supplementary data, including Figures S1–S38).

Moreover, semicarbazide and N’(2-phenylallyl)hydrazine were used as control, since they are well-established inhibitors of copper-containing amine oxidases [36]. The latter drug is also known as LJP 1207, the development of which has been discontinued as a consequence of its potential toxicity [54]. Thus, its synthesis was reproduced using the protocol described in [58]. This was also the case for 3-(4-Methylthiophenyl)-propylamine, characterized by the group of Unzeta as a good SSAO substrate [51], in-house synthetized and elsewhere named as MTPpropylamine. In this comparative study, was also included 1,12-diaminododecane (DIADO), previously reported to be a good substrate for the human SSAO/VAP-1 by Bonaiuto and coworkers [49], and which was a kindly gift from Maria Luisa Di Paolo (Univ. Padova, Padova, Italy).

### 3.2. Human Adipose Tissue Samples and Preparation of Adipocyte Suspensions

Samples of subcutaneous abdominal adipose tissue were obtained from a total of 24 non-obese women undergoing reconstructive surgery at Rangueil hospital, Toulouse, France: mean body mass index 31.6 ± 2.9 kg/m^2^, mean age of 39 years (range: 26–52). The surgically removed pieces of human adipose tissue (hAT) were immediately transported to the laboratory following agreement of the INSERM guidelines and of the local ethics committee for the protection of individuals (Comité de Protection des Personnes Sud Ouest et Outre-Mer II) under the reference: DC-2014–2039. The hAT pieces were immediately subjected to collagenase digestion at 37 °C to obtain freshly isolated adipose cells as described in [59], while small samples were stored at −80 °C until amine oxidase assays.

### 3.3. Biological Analyses

Homogenates were prepared with freshly thawed subcutaneous hAT samples by using homogenizer Tissue Master-125 (Omni International, Kennesaw, GA, USA) for approximate 30 s at room temperature. SZV agents were tested on in parallel biological material in a blinded fashion, i.e., handled by investigators who only know their code number but were unaware of their chemical structure.

Hydrogen peroxide release by hAT was measured using the fluorescent probe Amplex Red (10-acetyl-3,7-dihydrophenoxazine) as initially described by Zhou et al. for the continuous fluorometric determination of MAO activity [60], with already reported minor adaptations [61]. The inhibition by 1 mM semicarbazide of the amine-induced hydrogen peroxide release was also used to determine SSAO-dependent activity as in [61].

Inhibition of benzylamine oxidation was determined by a radiochemical method using 0.5 mM [^14^C]-benzylamine incubated for 30 min at 37 °C together with homogenates (approximately 100 µg proteins) and without or with the tested agents, as already described [62]; then counted by liquid scintillation as in [63]. Protein quantification was performed using DC Protein Assay kit (BioRad, Hercules, CA, USA).

[^3^H]-2-deoxyglucose uptake assay was performed for 10 min after 45 min incubation with the indicated agents, as already detailed [62]. Hexose uptake was normalized as a percentage of maximal stimulation by insulin.

The tests of the antilipolytic properties of SZV agents were performed in 400 µL of fat cell suspension. The agents were incubated with the fat cells at 37 °C under constant, gentle, shaking for 90 min. Incubations were stopped by placing the incubation tubes on ice. Once the buoyant adipocytes were frosted at the surface, 150 µL of medium were removed for glycerol release determination by spectrophotometric measurement as previously described [64].

This technique also applied for rat adipocytes, which were prepared from eight males bred at CREFRE (Centre Régional d’Exploration Fonctionnelle et Ressources Expérimentales, Toulouse, France), kept in plastic boxes with ad libitum access to standard rodent chow and water under controlled environment. All animal procedures complied with the principles established by the Institut National de la Santé et de la Recherche Médicale (INSERM, Paris, France) and were approved by the local Ethics Committee.

## 4. Conclusions

Among the novel SSAO substrates, SZV-2016 and SZV-2017 were capable of surpassing the effect of benzylamine, regarding hydrogen peroxide production, hexose uptake activation or glycerol release inhibition. Two other compounds, SZV-2118 and SZV-2124, stimulated hydrogen peroxide release and glucose entry, even better than benzylamine itself. However, they cannot be proposed simply as SSAO substrates since they inhibited lipolysis in a manner that was, at 1 mM, unexpectedly stronger than benzylamine and other SZV compounds. Moreover, our observations confirmed that phenylbutylamine is able to reproduce and even to overpass the SSAO-dependent effects of benzylamine hydrogen peroxide production, hexose uptake activation or glycerol release inhibition. Finally, SZV-2043 behaved as benzylamine, with regard to its insulin-like actions in human adipocytes. Surprisingly, MTPpropylamine, which was considered as a SSAO substrate of reference [51], did not surpass benzylamine when one takes into account its capacity to stimulate hexose uptake, while it was a potent antilipolytic agent in a manner that appeared to be independent from SSAO activation. All these agents activate glucose utilization and also inhibit lipolysis in hAT. Consequently, they can be deemed with potential antidiabetic properties, improving glucose disposal at the expense of a moderate fattening, as seen with the antidiabetic thiazolidinedione drugs.

Regarding the presumed novel SSAO inhibitors, the best hits were SZV-2142 and SZV-2007, with SZV-2045 to a lesser degree. However, these three agents inhibited the SSAO activity found in human adipose cells less strongly than phenylallylhydrazine or semicarbazide. Their only advantage consists, therefore, in the fact that they do not belong to the family of hydrazines and that they are thereby supposed to exert less unwanted toxic reactions, if used for long-term treatments.

## Figures and Tables

**Table 1:** Antilipolytic effect of SSAO substrates in human adipocytes are detected on forskolin-stimulated lipolysis and in the presence of the α2-AR antagonist methoxyidazoxan.

Glycerol Release (% of Maximal Lipolysis)	Isoprenaline 10 nM	Isoprenaline 10 nM + Methoxyidazoxan 10 µM	Forskolin 10 µM
control	77.6 ± 3.6	67.7 ± 7.2	64.9 ± 7.1
+ benzylamine 1 mM	16.1 ± 6.1 ***	27.8 ± 6.0 ***	33.1 ± 6.6 **
+ phenylbutylamine 1 mM	15.5 ± 3.2 ***	17.1 ± 3.8 ***	23.8 ± 5.8 ***
+ methylthiophenylpropylamine 1 mM	5.4 ± 2.8 ***	4.3 ± 3.4 ***	20.0 ± 5.9 ***

Mean ± SEM of seven determinations. Significantly different from respective control at: ** *p* < 0.01; *** *p* < 0.001.

## Data Availability

Part of the data presented in this study is available upon reasonable request, under the authorization of INSERM, which recurrently supported authors’ research.

## Supplementary Materials

The experimental procedures for the synthesis and the NMR spectra of the studied SZV compounds can be downloaded at: https://www.mdpi.com/article/10.3390/molecules27196224/s1.

## Sample Availability

Samples of the SZV compounds are available from the authors as response to justified queries.
