# Supplementary material for: Effects of Chemical Structures Interacting with Amine Oxidases on Glucose, Lipid and Hydrogen Peroxide Handling by Human Adipocytes
Source: Molecules. 2022 Sep 22;27(19):6224. doi: 10.3390/molecules27196224 (PMC9571511; doi:10.3390/molecules27196224)
Supplement: Supplementary file 1 [file molecules-27-06224-s001.zip › molecules-1866167-supplementary.pdf]

molecules

MDPI

supporting information

Effects of Chemical Structures Interacting with Amine Oxidases on Glucose, Lipid and Hydrogen Peroxide Handling by Human Adipocytes

Christian Carpéné [1,2,*], Pénélope Viana [1,2], Zsuzsa Iffiú-Soltesz [1], Pál Tapolcsányi [3], Anna Ágota Földi [3], Péter Mátyus [3,4] and Petra Dunkel [3]

1 Institut des Maladies Métaboliques et Cardiovasculaires, INSERM UMR1297, Toulouse 31432, France
2 Team Dinamix, Institute of Metabolic and Cardiovascular Diseases (I2MC), Paul Sabatier University, Toulouse 31432, France
3 Department of Organic Chemistry, Semmelweis University, H-1092 Budapest, Hungary
4 E-Group ICT SOFTWARE, H-1027 Budapest, Hungary
* Correspondence: christian.carpene@inserm.fr

Contents

Experimental procedures for the synthesis and the ^{1}H-NMR and ^{13}C-NMR spectra of the studied compounds

Figures S1–S38

General

All reagents and solvents were purchased from commercial sources and were utilized without further purification. Melting points were determined on a Büchi-540 (Büchi Labortechnik AG, Flawil, Switzerland) capillary melting point apparatus and are uncorrected. The ^{1}H and ^{13}C NMR spectra were recorded at ambient temperature, in the solvent indicated, with a Varian Mercury Plus spectrometer (Agilent Technologies, Santa Clara, CA, USA) at a frequency of 400 or 100MHz or with a Bruker 500MHz (Bruker Biospin, Rheinstetten, Germany) spectrometer, at a frequency of 500 or 125MHz, and are reported in parts per million (ppm). Chemical shifts are given on the δ-scale relative to tetramthylsilane or the residual solvent signal as an internal reference. In reporting spectral data, the following abbreviations were used: s = singlet, d = doublet, t = triplet, m = multiplet, dd = doublet doublet, dm = doublet multiplet, tm = triplet multiplet, and br = broad. For structure elucidation, one-dimensional ^{1}H, ^{13}C, DEPT, two-dimensional ^{1}H, ^{1}H-COSY, ^{1}H, ^{13}C-HSQC, ^{1}H, ^{13}C-HMBC measurements were run. Elemental analyses were performed on a Carlo Erba 1012 apparatus (Thermo Fisher Scientific, Milan, Italy), analyses indicated by the symbols of the elements affording satisfactory results. For flash column chromatography purification, Kieselgel 60 (Merck 0.040–0.063mm) (Merck KGaA, Darmstadt, Germany) was used; for thin layer chromatography (TLC) analysis, silica gel 60 F_{254} (Merck) plates were applied. Solvent mixtures used for chromatography are always given in a v/v ratio. The structures of all compounds were consistent with their analytical and spectroscopic data.

The following compounds were prepared according to the literature procedures cited and had melting points and/or spectral data identical with the published values: 4-PBA (HCl salt), MTPpropylamine [1], SZV-2045 and SZV-2043 [2], SZV-2007 [3], LJP1207 [4].

The following compounds were prepared as described below and had melting points and/or spectral data identical with the published values: SZV-2017 [5].

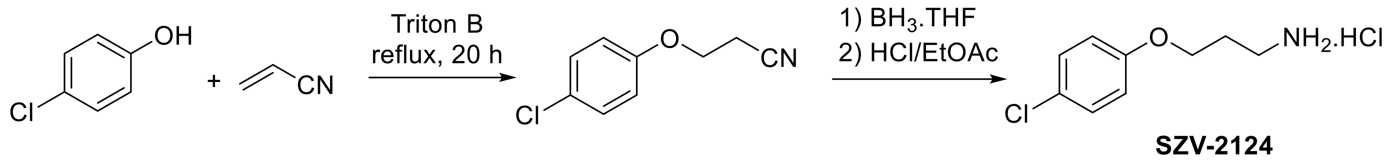

3-(4-chlorophenoxy)propanenitrile

A two-neck flask was flushed with argon and charged with the reagents: 4-chlorophenol (5.12 g, 40.00 mmol, 1.0 equiv), acrylonitrile (50 mL, 40.00 mmol, 1 equiv) and Triton B (40 wt% in methanol, 0.8 mL). The mixture was heated to reflux for 20 h. After cooling to rt, 120 mL DCM was added. The mixture was washed with 2 × 75 mL 5% NaOH, 1 × 60 mL 2M HCl and 1 × 60 mL water. The organic phase was dried over MgSO4, filtered and evaporated to dryness. The crude product was purified by column chromatography (DCM:hexane 1:1). White crystals (2.44 g, 34%), mp 46.4–47.5°C. 1H NMR (CDCl3) δ (ppm): 7.29–7.23 (m, 2H), 6.87–6.81 (m, 2H), 4.16 (t, J = 6.4 Hz, 2H), 2.83 (t, J = 6.4 Hz, 2H). 13C NMR (CDCl3) δ (ppm): 156.4, 129.7, 126.9, 117.1, 116.1, 63.0, 18.7.

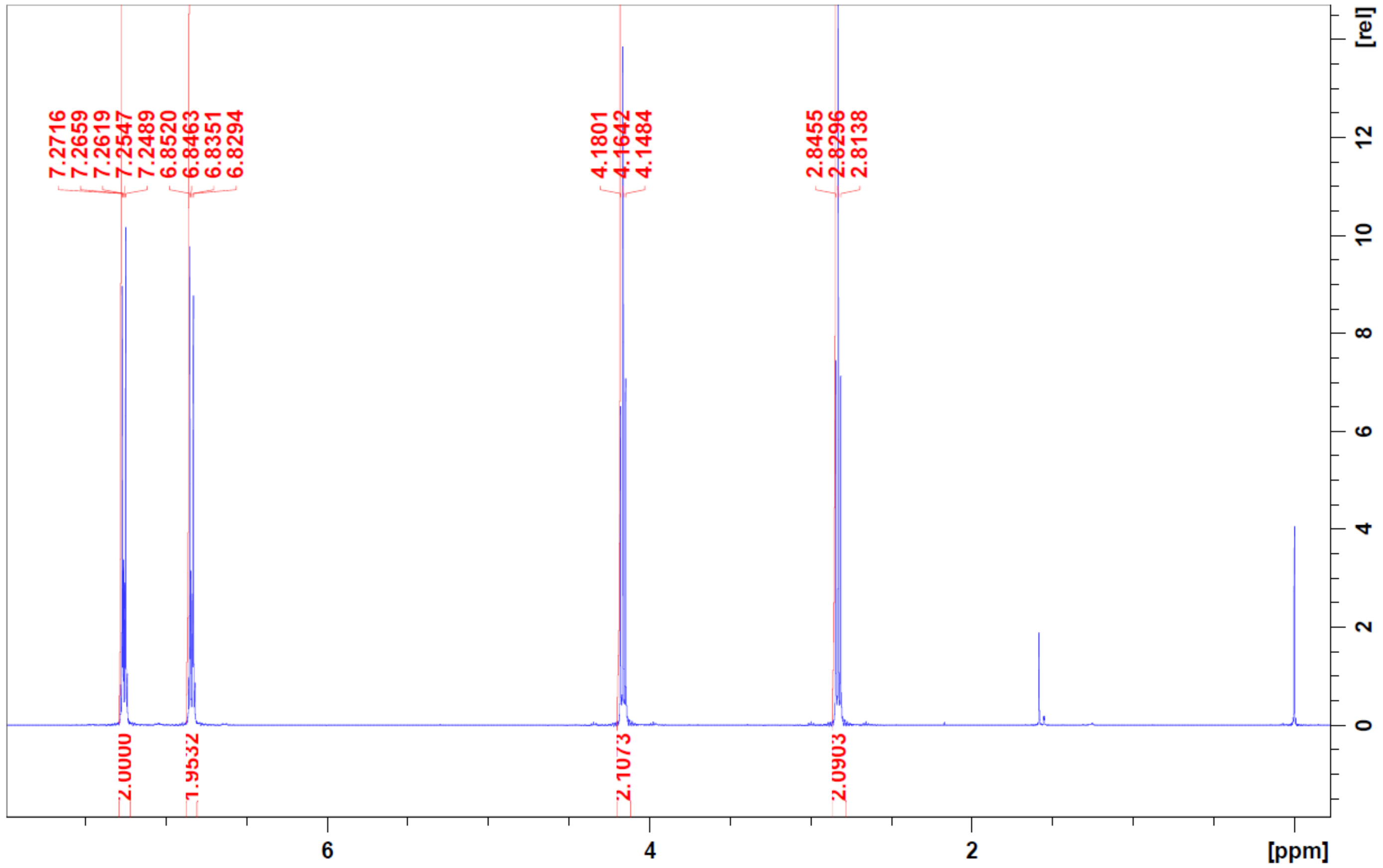

Figure S1. ^{1}H-NMR spectrum of 3-(4-chlorophenoxy)propanenitrile.

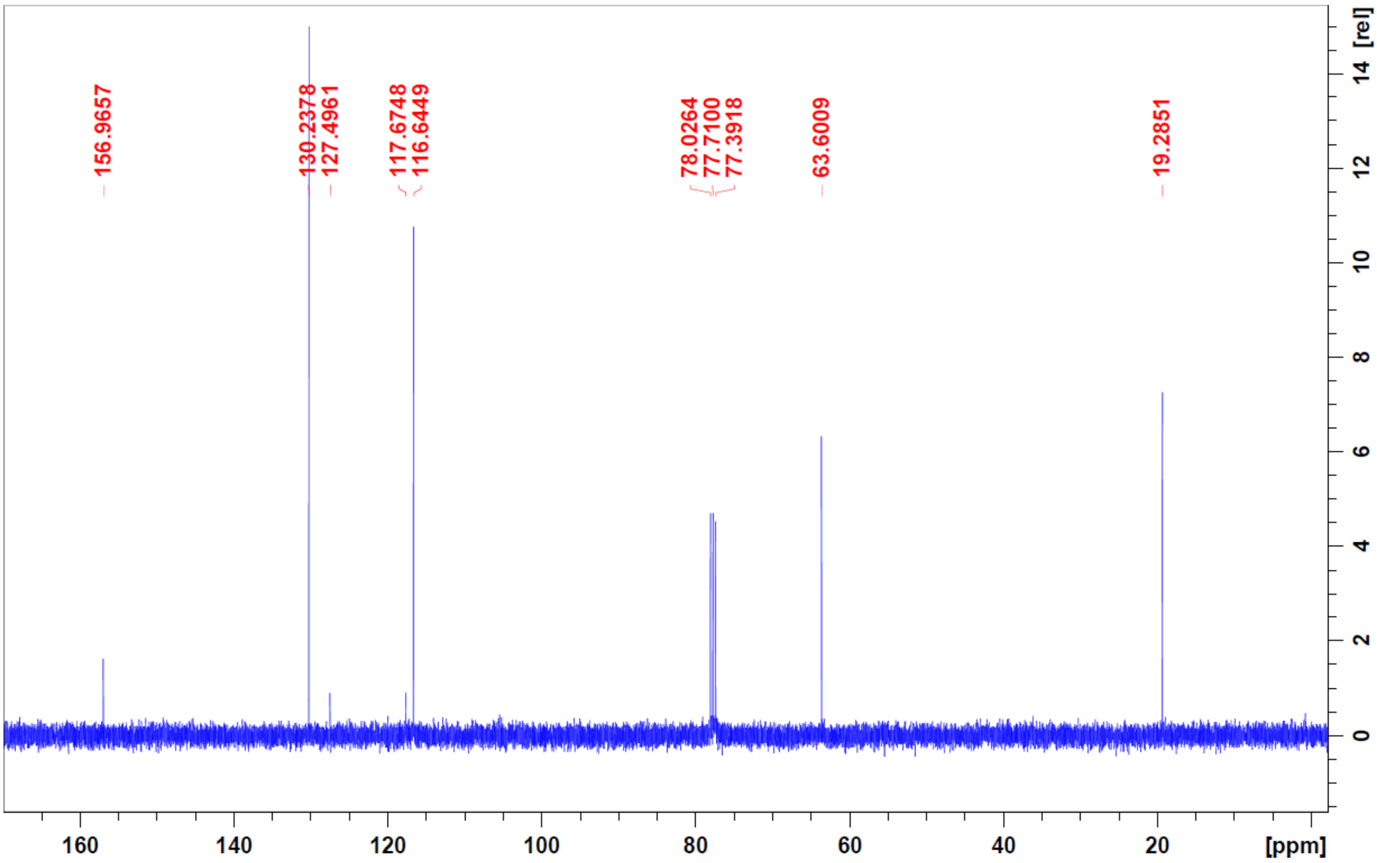

Figure S2. ^{13}C-NMR spectrum of 3-(4-chlorophenoxy)propanenitrile

3-(4-chlorophenoxy)propan-1-amine hydrochloride (SZV-2124)

A three-neck flask equipped with a thermometer and a condenser was flushed with argon and charged with 3-(4-chlorophenoxy)propanenitrile (1.82 g, 10.00 mmol, 1.0 equiv) dissolved in anhydrous THF (20 mL). The solution was cooled to 2–5°C and borane-THF (1 M solution in THF, 10 mL, 10.00 mmol, 1.0 equiv) was added dropwise, keeping the temperature at 5°C. After the addition of the reagent was completed, the reaction mixture was heated to reflux for 1.5 hours (reaction monitored with TLC). The reaction mixture was placed in an ice-water bath and 10 mL methanol was added dropwise. The mixture was heated to reflux for 30 min and then evaporated to dryness. To the residue 40 mL water was added. Upon cooling in an ice-water bath, a mixture of 5 mL cc. HCl and 5 mL water was added dropwise. The reaction mixture was heated to reflux for 20 min, before cooling it back to rt. The reaction mixture was extracted with 2 × 20 mL DCM. The pH of the aqueous phase was adjusted to >12 with 2 M NaOH and it was extracted with 4×30 mL DCM. The combined organic phases were dried over Na2SO4, filtered and evaporated to dryness. The crude product obtained was dissolved in 5 mL EtOAc, filtered (Whatman) and the filter was washed with an additional 5 mL EtOAc. Upon cooling in an ice-water bath HCl (4.37 M in EtOAc, 3 mL) was added dropwise (stirring till complete precipitate formation). The reaction mixture was evaporated to dryness, the product was recrystallized from EtOH. White crystals (940 mg, 42%), mp 179.0–179.5°C. 1H NMR (DMSO-d6) δ (ppm): 8.21 (br s, 2H), 7.35–7.29 (m, 2H), 6.99–6.93 (m, 2H), 4.05 (t, J = 6.2 Hz, 2H), 2.92 (t, J = 7.2 Hz, 2H), 2.03 (quint, J = 6.3 Hz, 2H). 13C NMR (DMSO-d6) δ (ppm): 157.1, 129.1, 124.3, 116.2, 64.9, 36.0, 26.6. Anal. (C9H13Cl2NO) calcd. C 48.67; H 5.90; N 6.31. Found: C 48.91; H 5.53; N 6.25.

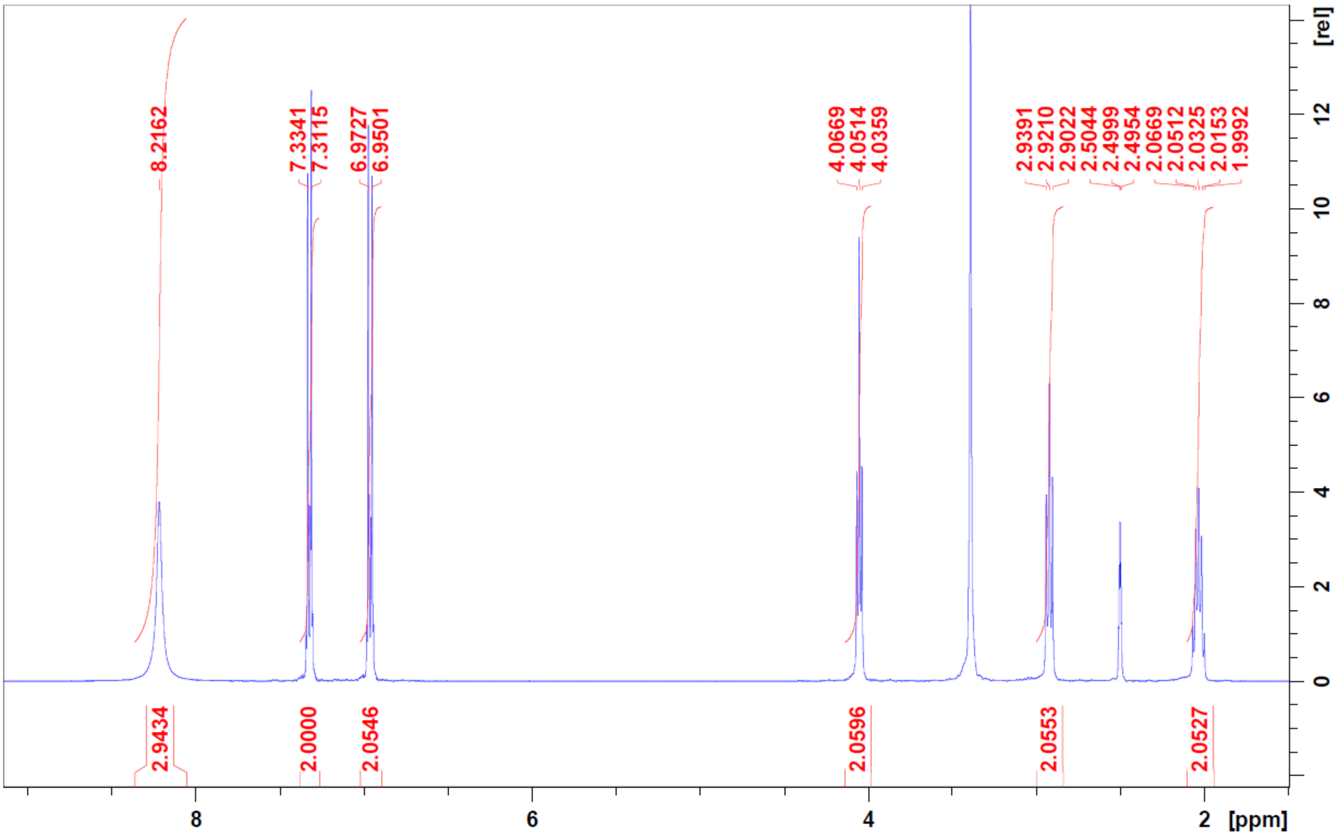

Figure S3. ^{1}H-NMR spectrum of 3-(4-chlorophenoxy)propan-1-amine hydrochloride

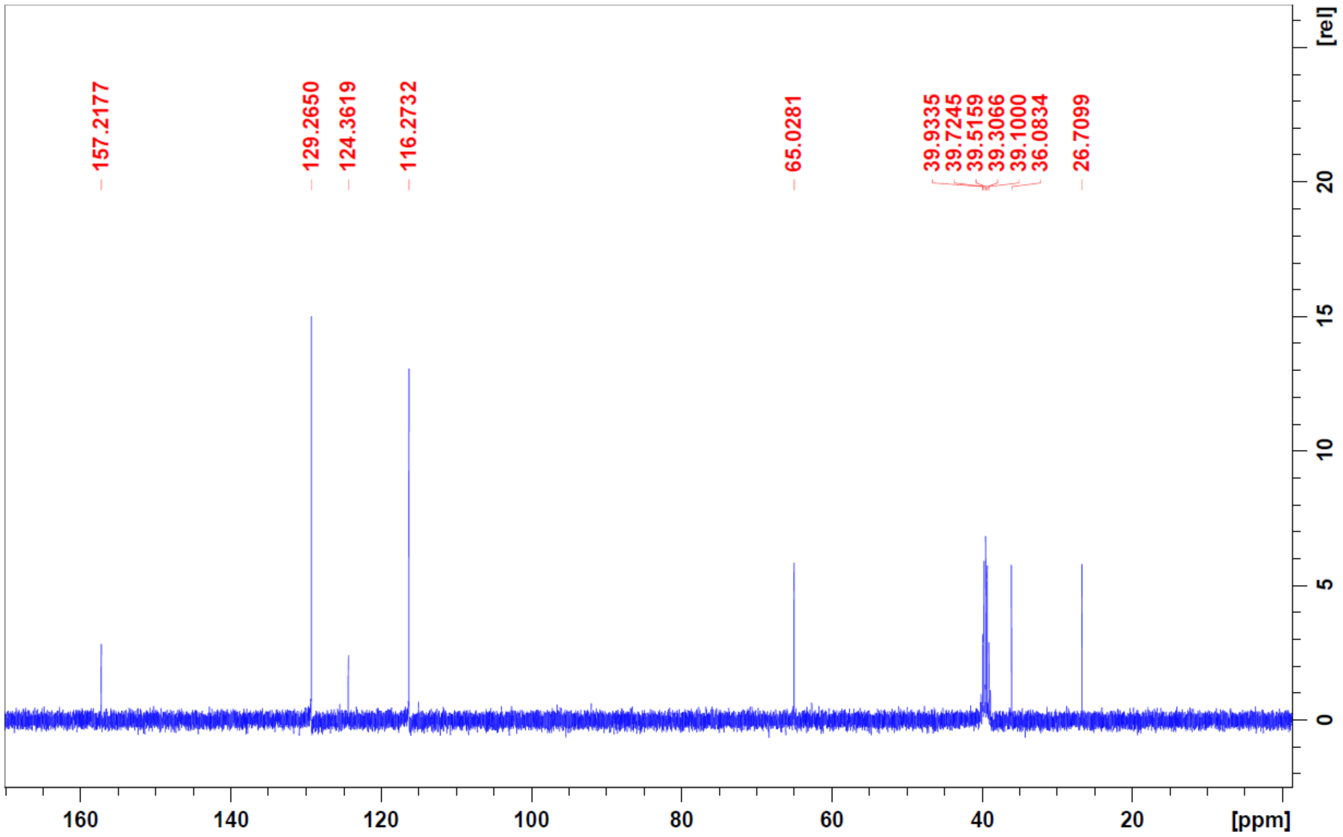

Figure S4. ^{13}C-NMR spectrum of 3-(4-chlorophenoxy)propan-1-amine hydrochloride

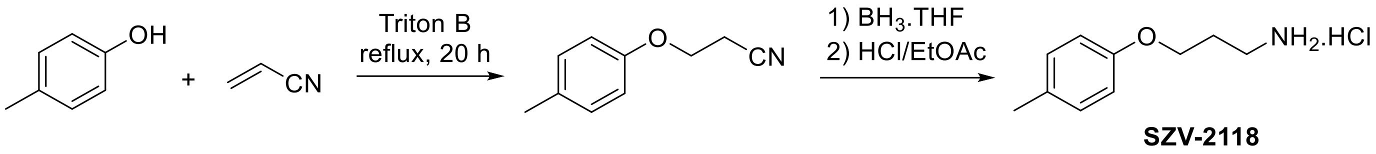

3-(4-methylphenoxy)propanenitrile

A two-neck flask was flushed with argon and charged with the reagents: *p*-cresol (1.08 g, 10.00 mmol, 1.0 equiv), acrylonitrile (12 mL, 10.00 mmol, 1 equiv) and Triton B (40 wt% in methanol, 0.2 mL). The mixture was heated to reflux for 24 h. After cooling to rt, 30 mL diethyl ether was added. The mixture was washed with 2×20 mL 5% NaOH, 1×20 mL 2M HCl and 1×20 mL water. The organic phase was dried over Na_2SO_4, filtered and evaporated to dryness. The crude product was purified by column chromatography (DCM:hexane 1:1). White crystals (1.20 g, 75%), mp 46.8–47.7 °C. ^{1}H NMR ($CDCl_3$) δ (ppm): 7.12–7.07 (m, 2H), 6.83–6.78 (m, 2H), 4.15 (t, *J* = 6.4 Hz, 2H), 2.79 (t, *J* = 6.4 Hz, 2H), 2.29 (s 3H). ^{13}C NMR ($CDCl_3$) δ (ppm): 155.7, 131.3, 130.2, 117.4, 114.7, 62.9, 20.6, 18.7. Anal. ($C_{10}H_{11}NO$) calcd. C 74.51; H 6.88; N 8.69. Found: C 74.63; H 6.84; N 8.69.

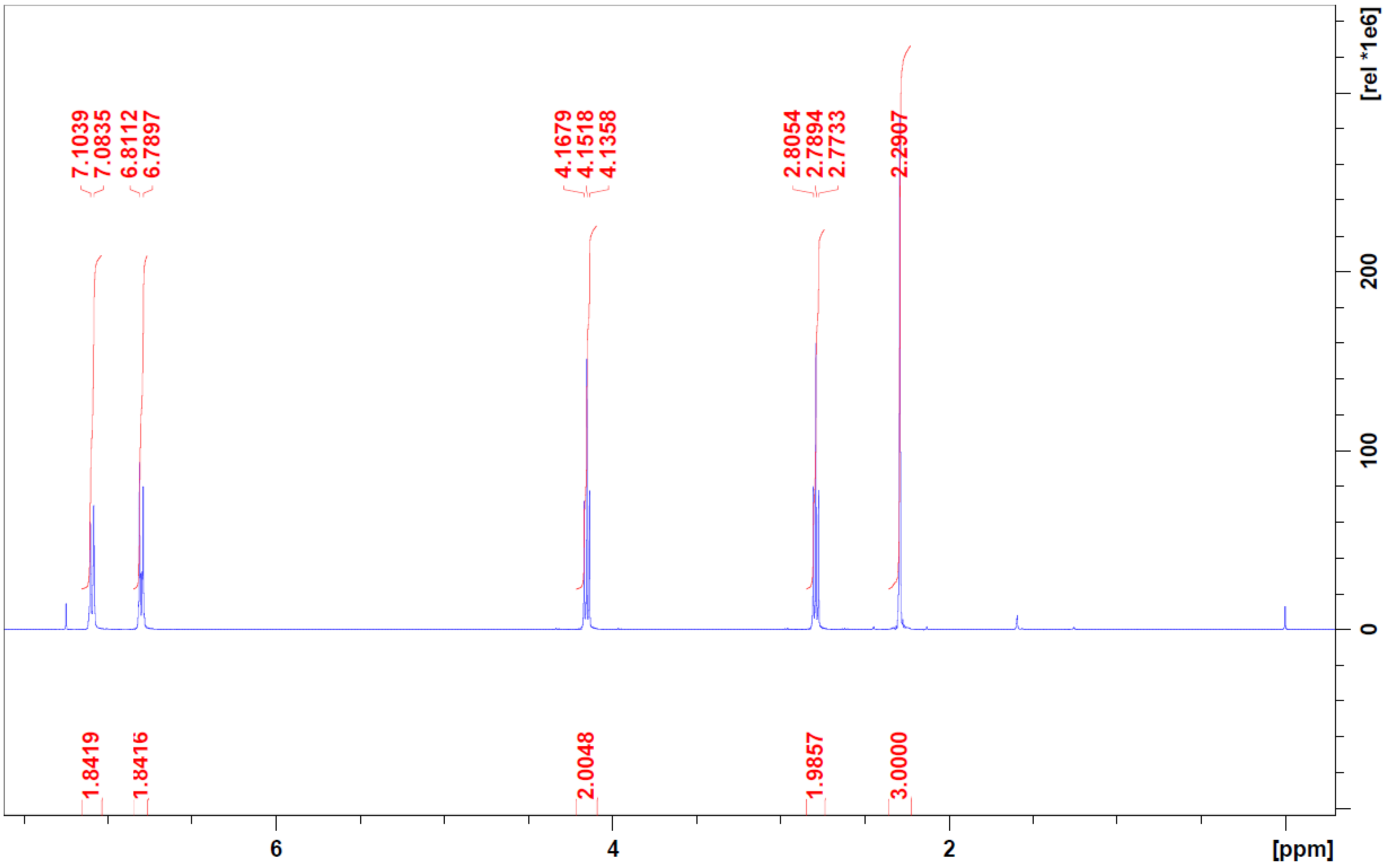

Figure S5. ^{1}H-NMR spectrum of 3-(4-methylphenoxy)propanenitrile

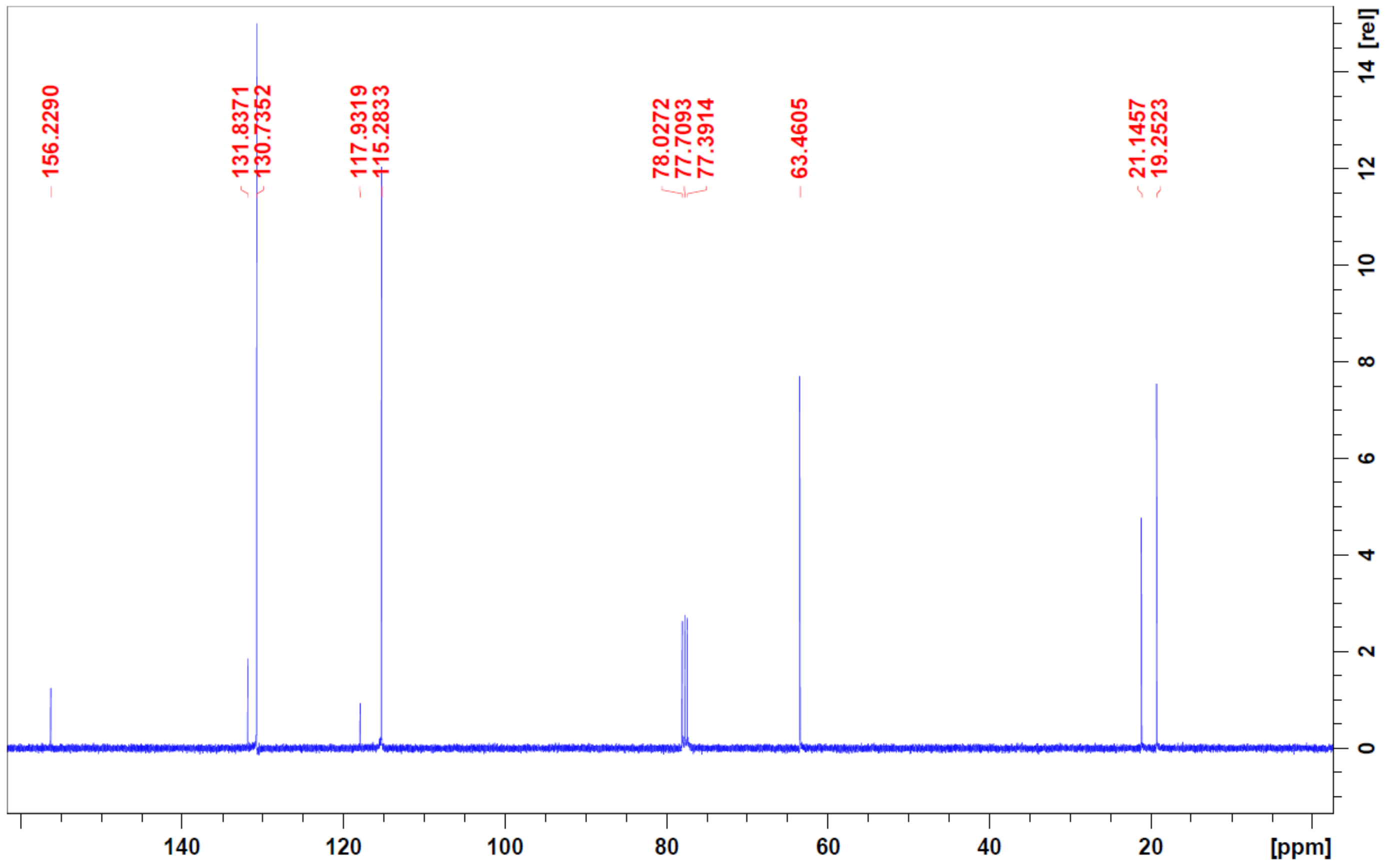

Figure S6. ^{13}C-NMR spectrum of 3-(4-methylphenoxy)propanenitrile

3-(4-methylphenoxy)propan-1-amine hydrochloride (SZV-2118)

A three-neck flask equipped with a thermometer and a condenser was flushed with argon and charged with 3-(4-methylphenoxy)propanenitrile (806 mg, 5.00 mmol, 1.0 equiv) dissolved in anhydrous THF (40 mL). The solution was cooled to 5°C and borane-THF (1 M solution in THF, 12 mL, 5.00 mmol, 1.0 equiv) was added dropwise, keeping the temperature at 5°C. After the addition of the reagent was completed, the reaction mixture was heated to reflux for 2.5 hours (reaction monitored with TLC). The reaction mixture was placed in an ice-water bath and 10 mL methanol was added dropwise, followed by 1.5 mL water. The mixture was left overnight at rt. The following day the mixture was evaporated to dryness, then 20 mL water was added. Upon cooling in an ice-water bath, a mixture of 2 mL cc. HCl and 2 mL water was added dropwise. The reaction mixture was extracted with 1×20 mL DCM. The pH of the aqueous phase was adjusted to >12 with 2 M NaOH and it was extracted with 3×20 mL DCM. The combined organic phases were dried over Na2SO4, filtered and evaporated to dryness. The crude product obtained was dissolved in 10 mL EtOAc and upon cooling in an ice-water bath HCl (4.3 M in EtOAc, 2 mL) was added dropwise (stirring till complete precipitate formation). The reaction mixture was evaporated to dryness, the product was recrystallized from EtOH. White crystals (291 mg, 29%), mp 198.1–199.9°C. 1H NMR (CDCl3) δ (ppm): 8.07 (br s, 2H), 7.12–7.06 (m, 2H), 6.86–6.80 (m, 2H), 4.00 (t, J = 6.2 Hz, 2H), 2.92 (t, J = 7.2 Hz, 2H), 2.22 (s, 3H), 2.00 (quint, J = 7.6 Hz, 2H). Anal. (C10H16ClNO) calcd. C 59.55; H 8.00; N 6.94. Found: C 59.38; H 8.23; N 6.89.

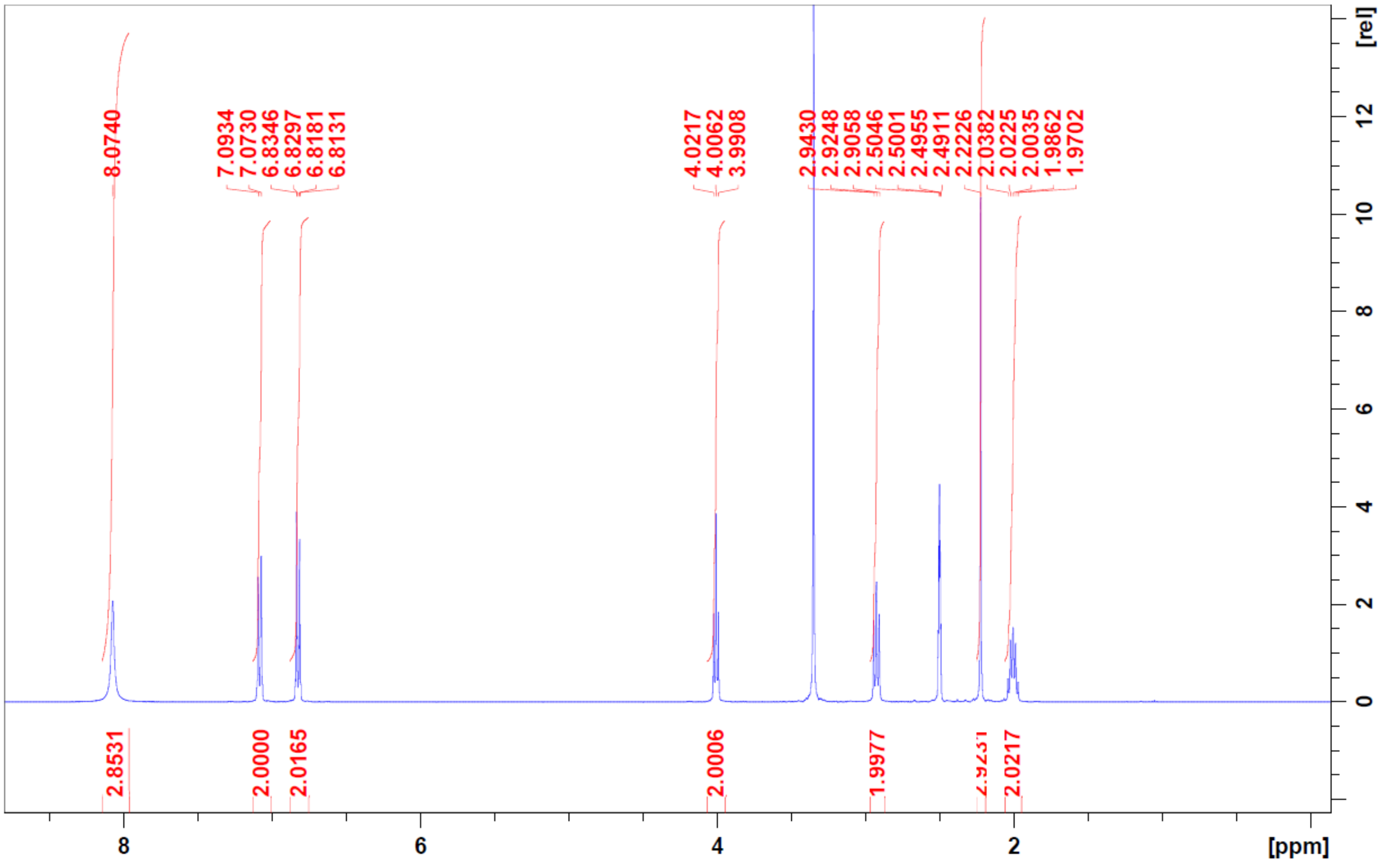

Figure S7. ^{1}H-NMR spectrum of 3-(4-methylphenoxy)propan-1-amine hydrochloride

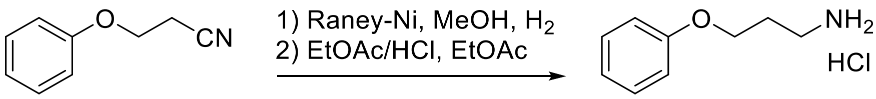

SZV-2017

3-phenoxypropan-1-amine hydrogen chloride (SZV-2017)

To a solution of 3-phenoxypropanenitrile (4.50 g, 30.00 mmol) in methanol (100 mL) Raney-Ni (1.20 g) was added and the mixture was stirred at 110°C for 7 h under hydrogen (40 bar). After filtration the solvent was distilled off and the crude product was purified with column chromatography (CHCl3:MeOH:H2O 60:12:1). The obtained product (2.80 g) was dissolved in EtOAc-EtOH and EtOAc/HCl was added dropwise till pH 2–3. The precipitated crystals were filtered off, washed with EtAOc and EtOH and dried under vacuum. White crystals (2.53 g, 45%), mp 175.0–176.0°C. 1H NMR (DMSO-d6) δ (ppm): 8.10 (br s, 3H), 7.33–7.25 (m, 2H), 6.97–6.90 (m, 2H), 4.05 (t, J = 6.2 Hz, 2H), 2.94 (t, J = 7.3 Hz, 2H), 2.02 (quint, J = 7.1 Hz, 2H). Anal. (C9H14ClNO) calcd. C 57.60; H 7.52; N 7.46. Found: C 57.47; H 7.26; N 7.38.

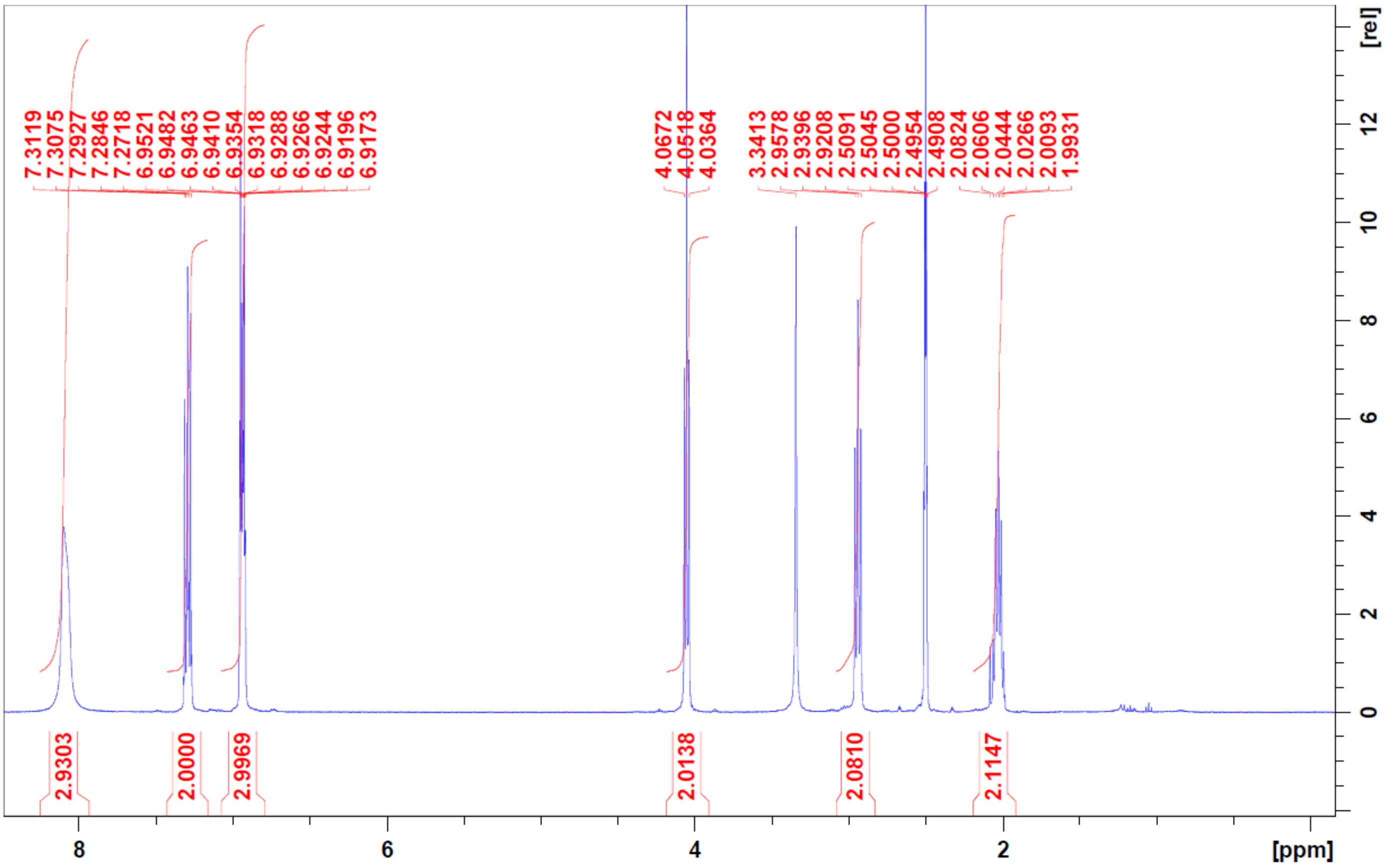

Figure S8. ^{1}H-NMR spectrum of 3-phenoxypropan-1-amine hydrogen chloride

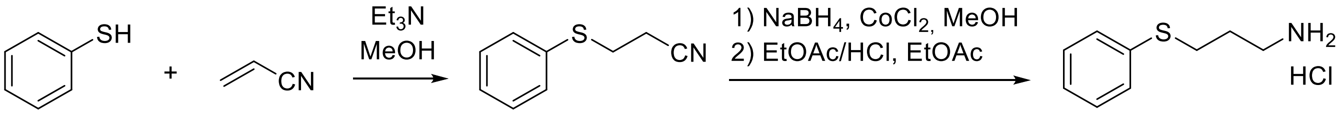

SZV-2016

3-(phenylsulfanyl)propan-1-amine hydrochloride (SZV-2016)

To a solution of 3-(phenylsulfanyl)propanenitrile (prepared according to [6] (5.71 g, 35.00 mmol) in methanol (210 mL) CoCl2×6H2O (16.66 g, 70.00 mmol, 2.0 equiv) was added. Upon cooling NaBH4 (13.30 g, 350.00 mmol, 10.0 equiv) was added in small portions. After the addition of the reagent the mixture was stirred at rt for 1 h. The reaction was quenched with 3 M HCl (110 mL) and the methanol was distilled off. The residue was extracted with diethyl ether. The pH of the aqueous phase was adjusted to >10 with 3% NaOH and it was extracted with DCM. The combined DCM phases were evaporated to dryness and purified with column chromatography (CHCl3:MeOH:H2O 60:12:1) to yield the amine product (2.19 g, 37%). A portion of the amine product (0.73 g, 4.36 mmol) was dissolved in EtOAc and treated with EtOAc/HCl to yield the hydrochloride salt. White crystals (0.78 g, quant.), mp 163.0–167.0°C. Anal. (C9H14ClNS) calcd. C 53.06; H 6.93; N 6.88. Found: C 51.98; H 6.49; N 6.74.

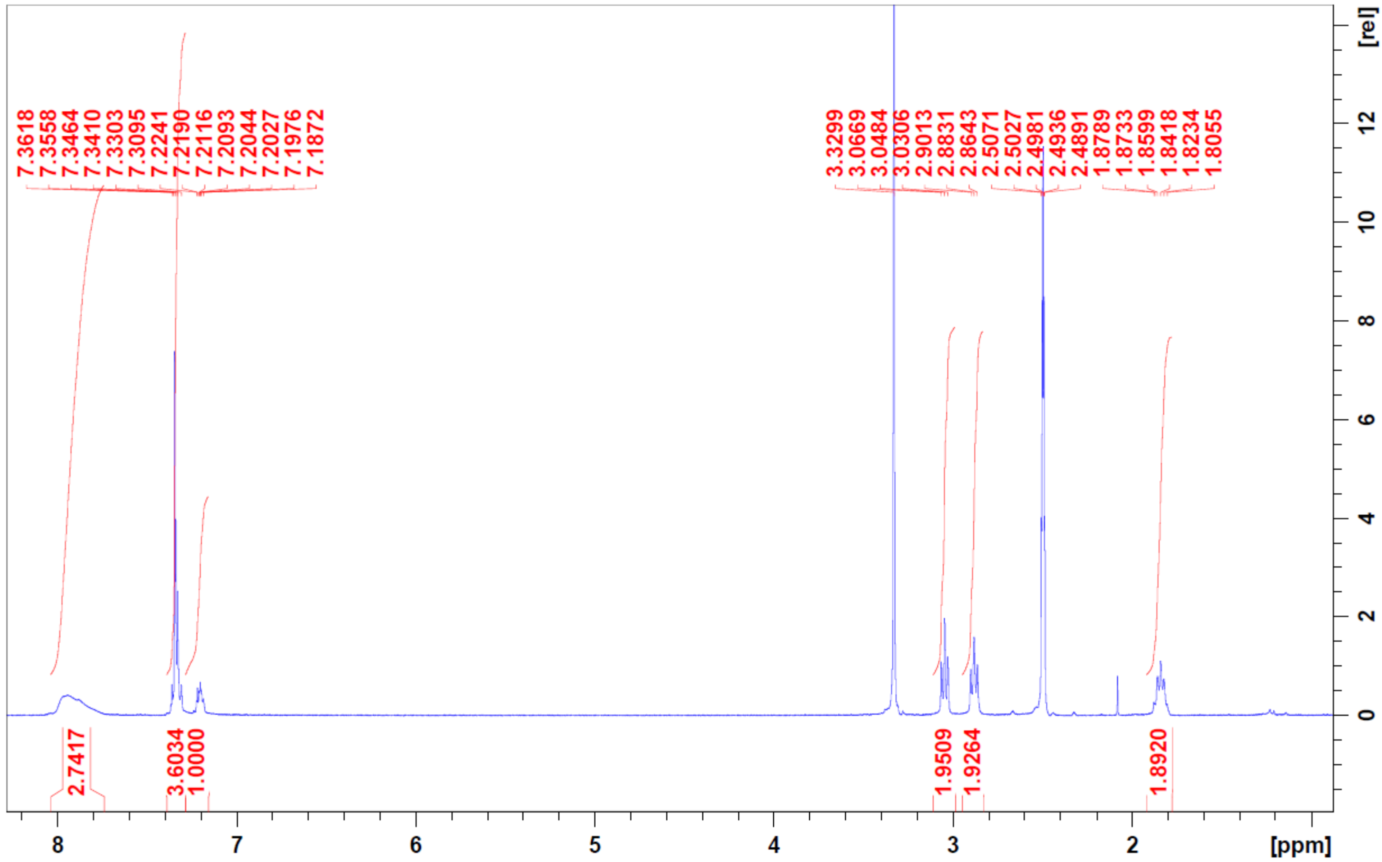

Figure S9. ^{1}H-NMR spectrum of 3-(phenylsulfanyl)propan-1-amine hydrochloride

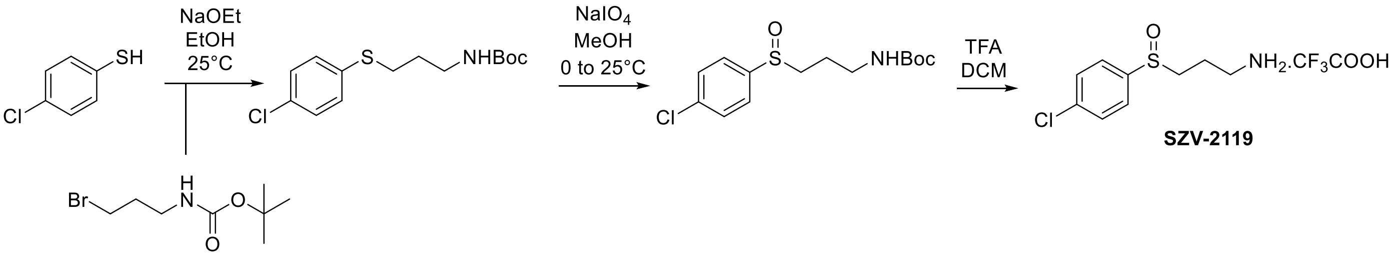

tert-butyl {3-[(4-chlorophenyl)sulfanyl]propyl}carbamate

A three-neck flask was charged with 20 mL EtOH and sodium (276 mg, 12.00 mmol, 1.5 equiv) was added (stirring till complete dissolution and cooling back to rt). Then 4-chlorothiophenol (1.74 g, 12.00 mmol, 1.5 equiv) was added, followed by 10 min stirring. Finally tert-butyl (3-bromopropyl)carbamate (1.91 g, 8.00 mmol, 1.0 equiv) was added to the reaction mixture. The reaction was stirred at rt for 18 h (reaction monitored with TLC). The reaction mixture was evaporated to dryness. Water (30 mL) was added and the mixture was extracted with 3×30 mL DCM. The combined organic phases were washed with brine (1×30 mL), dried over MgSO4, filtered and evaporated to dryness. The crude product was purified with column chromatography (toluene→DCM). White crystals (2.27 g, 94%), mp 62.7–63.8°C. 1H NMR (CDCl3) δ (ppm): 7.30–7.21 (m, 4H), 4.64 (br s, 1H), 3.23 (q, J = 6.4 Hz, 2H), 2.92 (t, J = 7.1 Hz, 2H), 1.80 (quin, J = 7.0 Hz, 2H), 1.43 (s, 9H). 13C NMR (CDCl3) δ (ppm): 156.0, 134.8, 132.1, 130.7, 129.2, 79.5, 39.5, 31.4, 29.5, 28.5. Anal. (C14H20ClNO2S) calcd. C 55.71; H 6.68; N 4.64. Found: C 55.77; H 6.77; N 4.70.

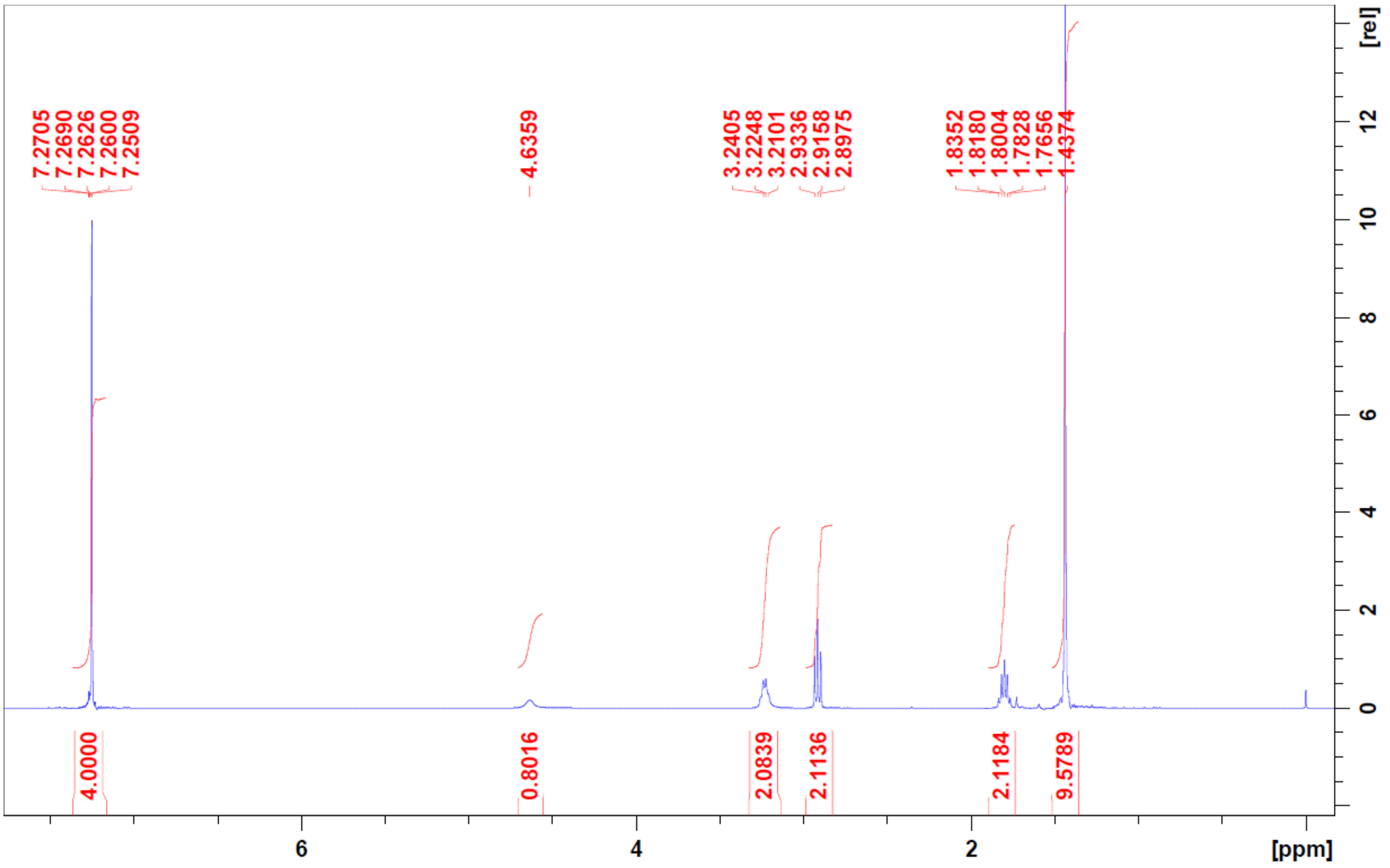

Figure S10. ^{1}H-NMR spectrum of *tert*-butyl {3-[(4-chlorophenyl)sulfanyl]propyl}carbamate

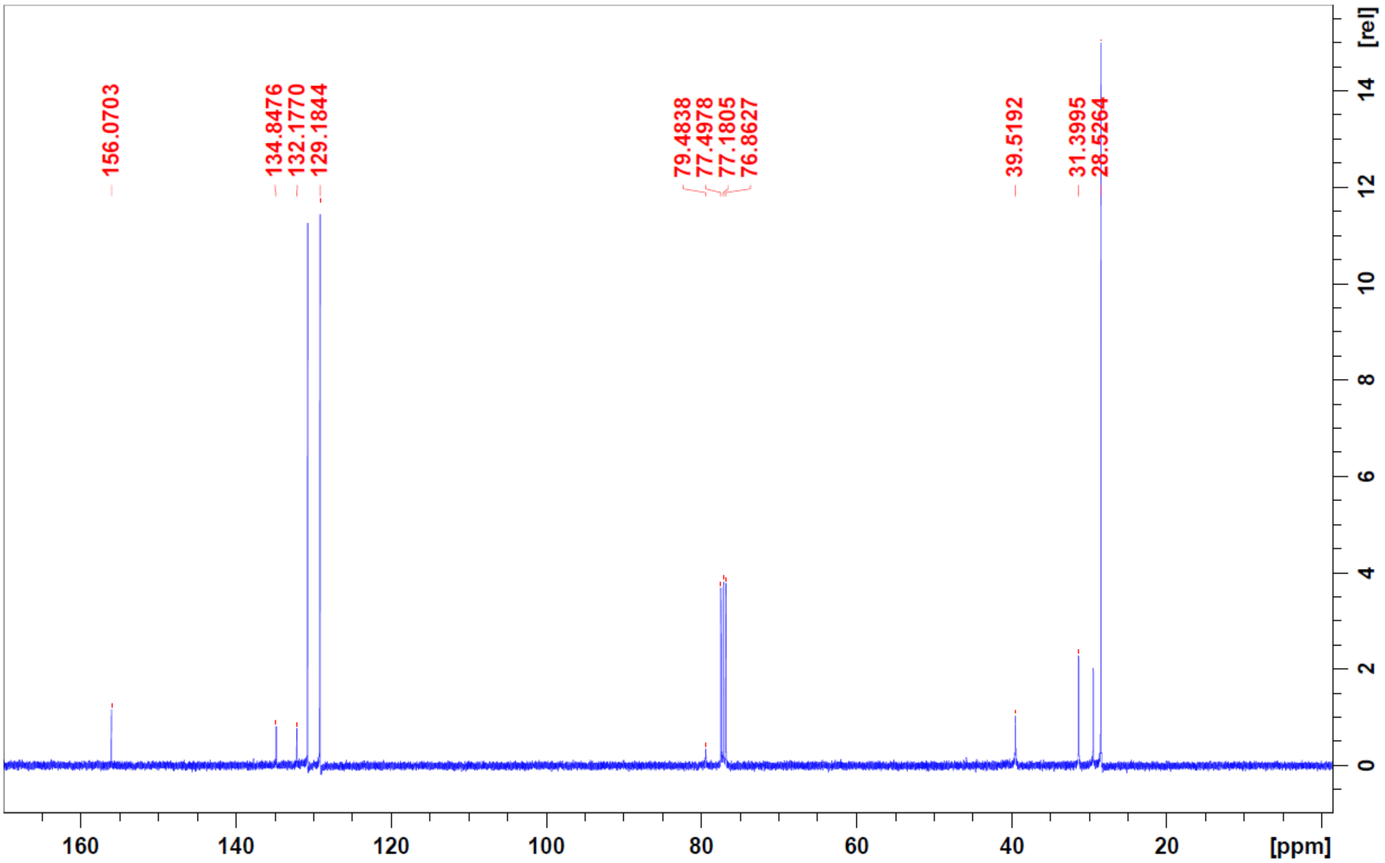

Figure S11. ^{13}C-NMR spectrum of *tert*-butyl {3-[(4-chlorophenyl)sulfanyl]propyl}carbamate

***tert*-butyl [3-(4-chlorobenzene-1-sulfinyl)propyl]carbamate**

A three-neck flask equipped with a thermometer and a dropping funnel was charged with NaIO4 (706 mg, 3.30 mmol, 1.1 equiv), methanol (8 mL) and water (2 mL). The white suspension was cooled to 0°C in an ice-water bath. A solution of tert-butyl {3-[(4-chlorophenyl)sulfanyl]propyl}carbamate (905 mg, 3.00 mmol, 1.0 equiv) in 2.4 mL methanol was added dropwise upon cooling (dropping funnel flushed with an additional 2×0.5 mL of methanol). The reaction mixture was let to warm up to rt and was stirred for 18 h (reaction monitored with TLC). The white precipitate was filtered off and washed with 3×20 mL EtOAc. The filtrate was washed with 2×20 mL water and 1×20 mL brine, dried over MgSO4, filtered and evaporated to dryness. The crude product was purified with column chromatography (EtOAc:CHCl3 4:6). The oil obtained after evaporation of the fractions was dissolved in CHCl3, filtered and evaporated to dryness. White crystals (809 mg, 95%), mp 84.3–84.9°C. 1H NMR (CDCl3) δ (ppm): 7.59–7.55 (m, 2H), 7.53–7.48 (m, 2H), 4.85 (br s, 1H), 3.34–3.19 (m, 2H), 2.94–2.84 (m, 1H), 2.83–2.72 (m, 1H), 2.05–1.93 (m, 1H), 1.84–1.71 (m, 1H), 1.43 (s, 9H). 13C NMR (CDCl3) δ (ppm): 156.1, 142.2, 137.3, 129.6, 125.5, 79.6, 54.8, 39.3, 28.5, 23.2. Anal. (C14H20ClNO3S) calcd. C 52.92; H 6.34; N 4.41. Found: C 53.07; H 6.09; N 4.37.

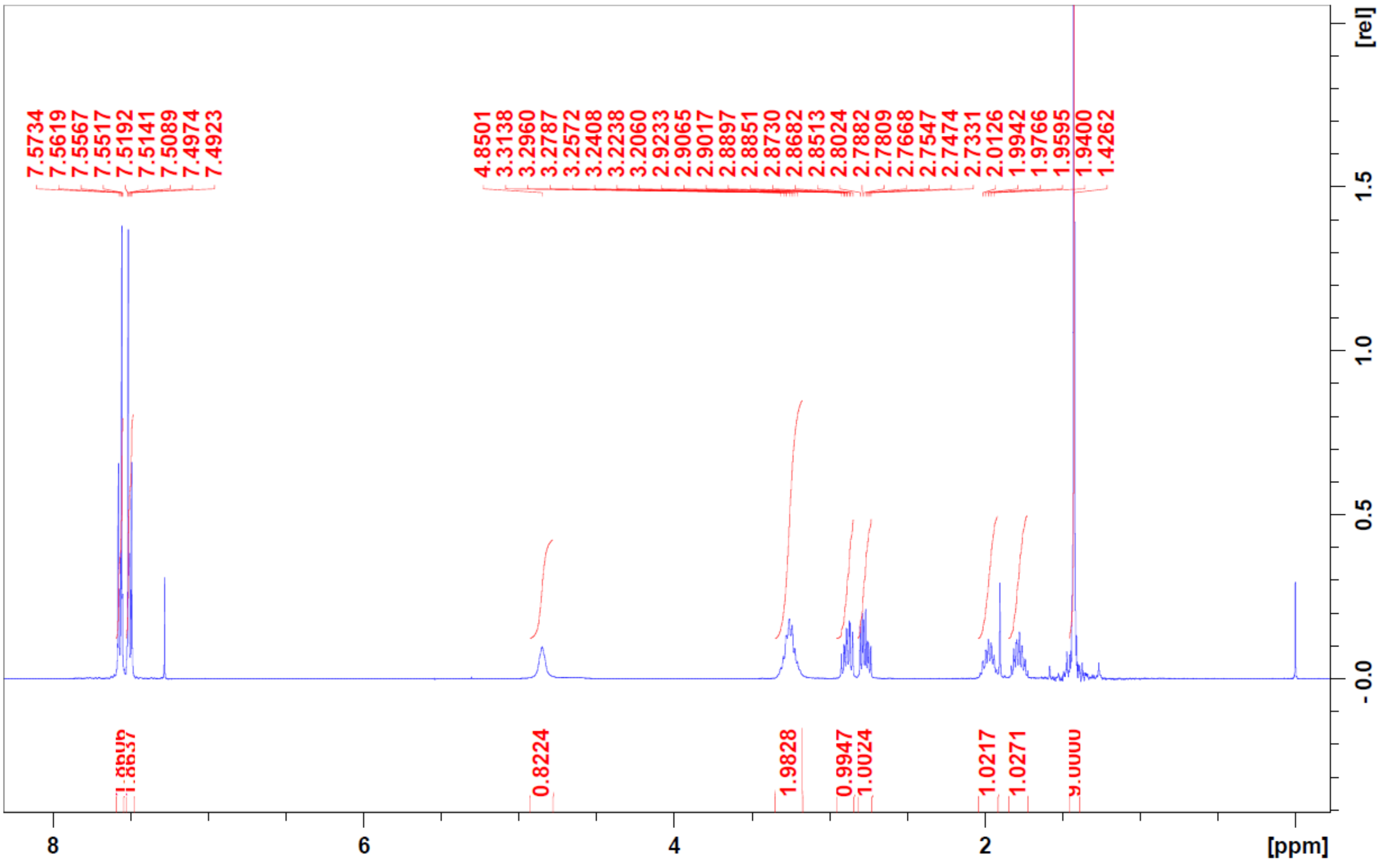

Figure S12. ^{1}H-NMR spectrum of *tert*-butyl [3-(4-chlorobenzene-1-sulfinyl)propyl]carbamate

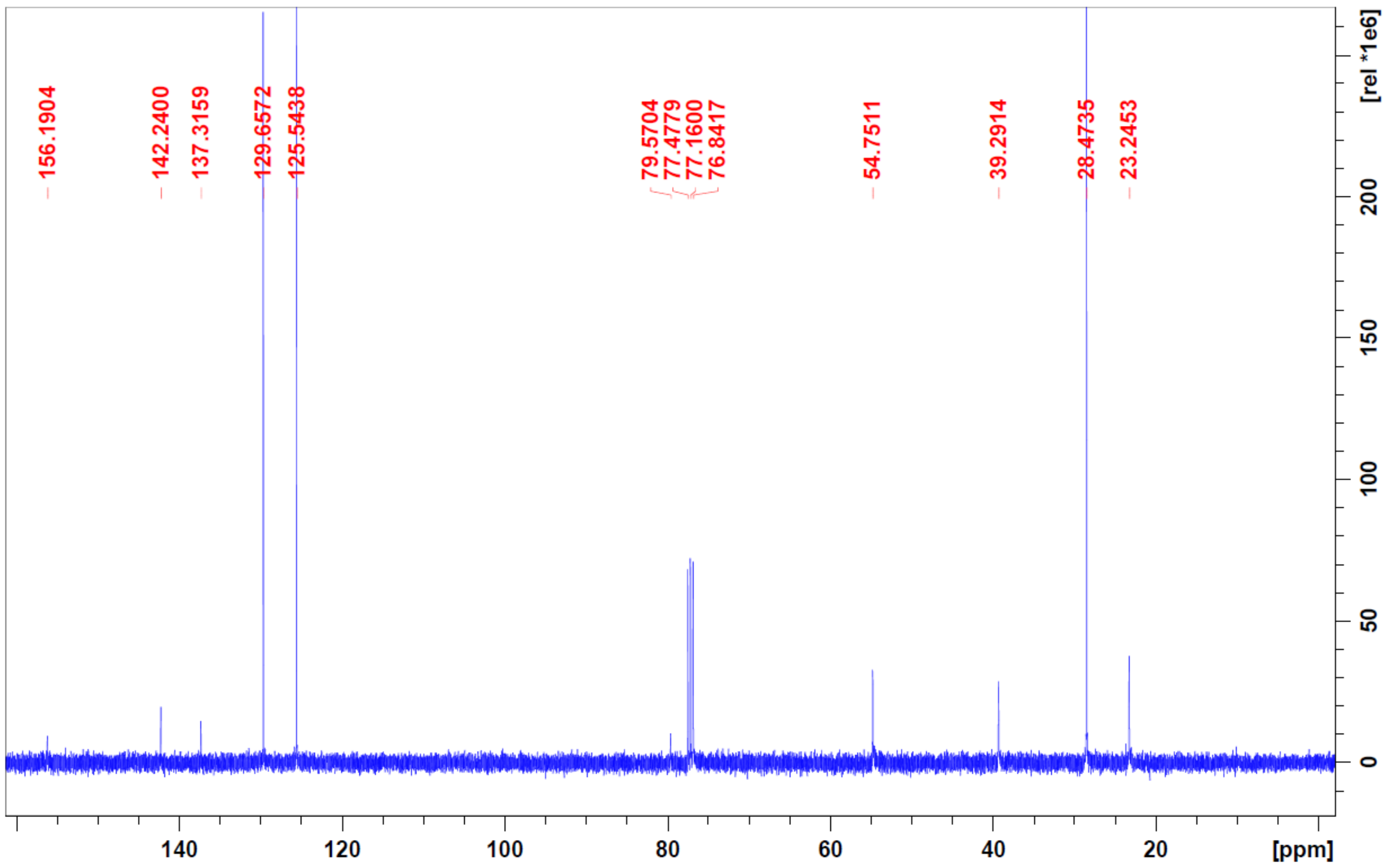

Figure S13. ^{13}C-NMR spectrum of *tert*-butyl [3-(4-chlorobenzene-1-sulfinyl)propyl]carbamate

3-(4-chlorobenzene-1-sulfinyl)propan-1-amine trifluoroacetate salt (SZV-2119)

To a solution of tert-butyl [3-(4-chlorobenzene-1-sulfinyl)propyl]carbamate (318 mg, 1.00 mmol) in DCM (10 mL) at 0°C TFA (3.30 mL) was added dropwise. The reaction mixture was let to warm up to rt and was stirred for 30 min (reaction monitored with TLC). The reaction mixture was evaporated to dryness, to the residue pentane was added. The suspension was aged overnight at 10–15°C. The following day the precipitated crystals were filtered off, washed with diethyl ether (2×5 mL) and pentane (2×5 mL), then dried under vacuum. White crystals (314 mg, 95%), mp 161.0–161.7°C. 1H NMR (DMSO-d6) δ (ppm): 7.85 (br s, 2H), 7.72–7.64 (m, 4H), 3.16–3.07 (m, 1H), 2.92–2.81(m, 3H), 1.97–1.84 (m, 1H), 1.72–1.59 (m, 1H). 13C NMR (DMSO-d6) δ (ppm): 142.5, 135.7, 129.4, 126.1, 51.5, 37.8, 19.6. Anal. (C11H13ClF3NO3S) calcd. C 39.83; H 3.95; N 4.22. Found: C 39.82; H 3.46; N 4.15.

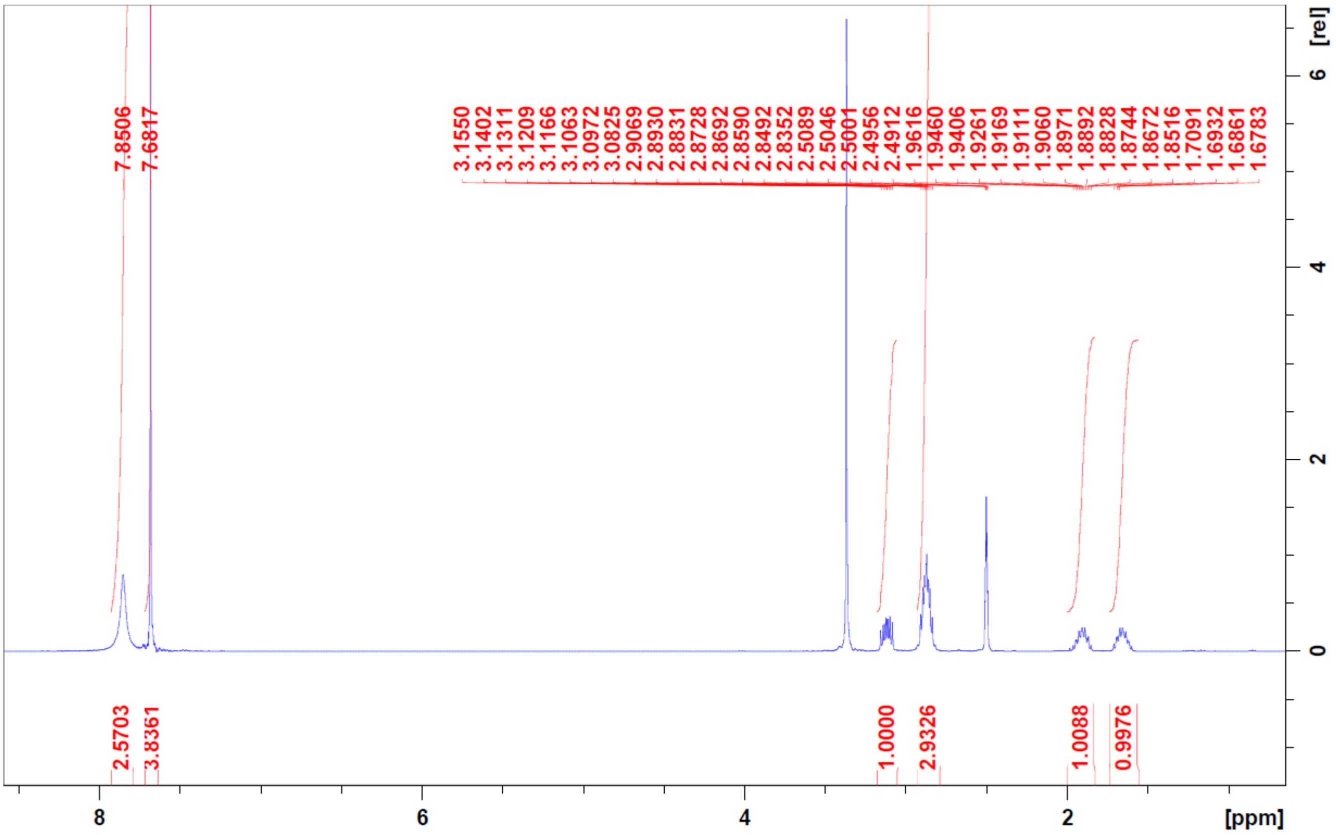

Figure S14. ^{1}H-NMR spectrum of 3-(4-chlorobenzene-1-sulfinyl)propan-1-amine trifluoroacetate salt

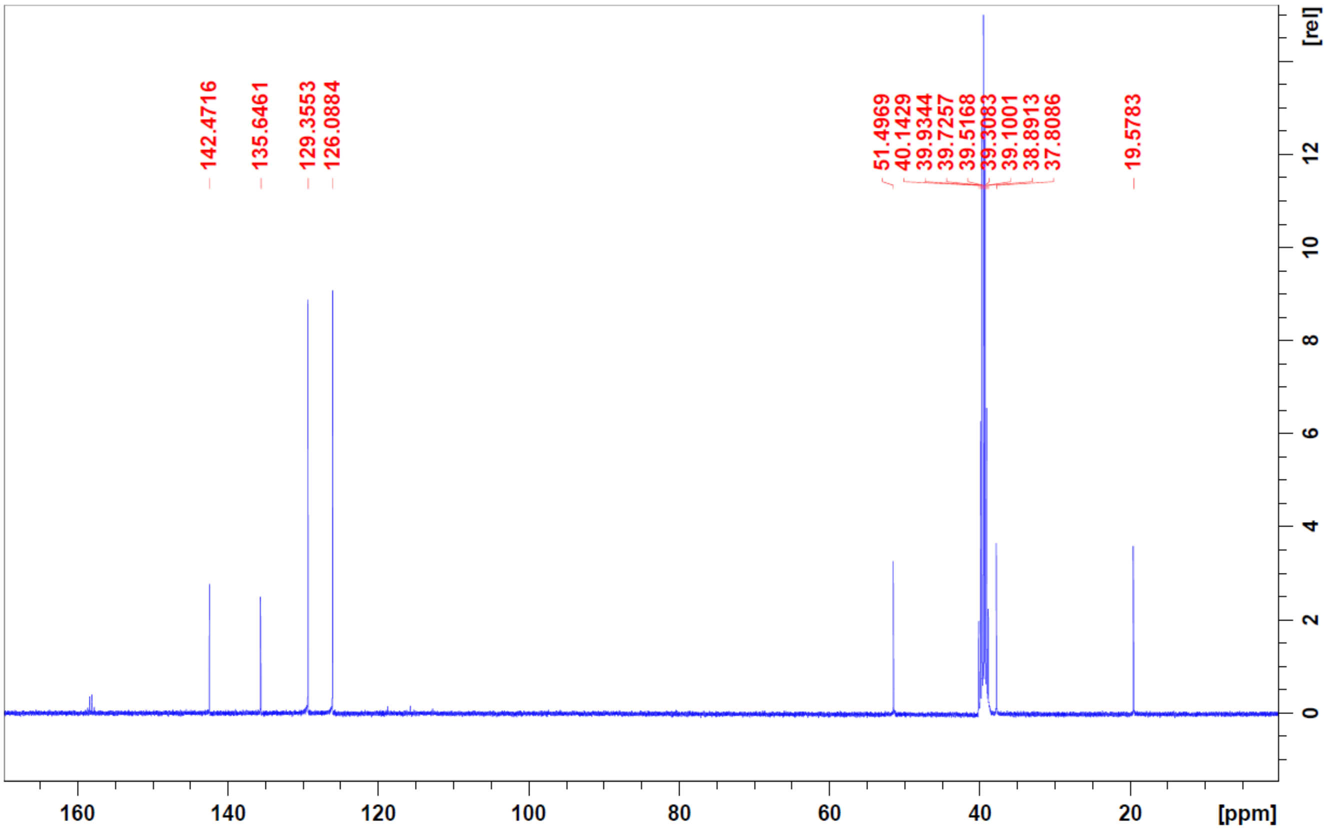

Figure S15. ^{13}C-NMR spectrum of 3-(4-chlorobenzene-1-sulfinyl)propan-1-amine trifluoroacetate salt

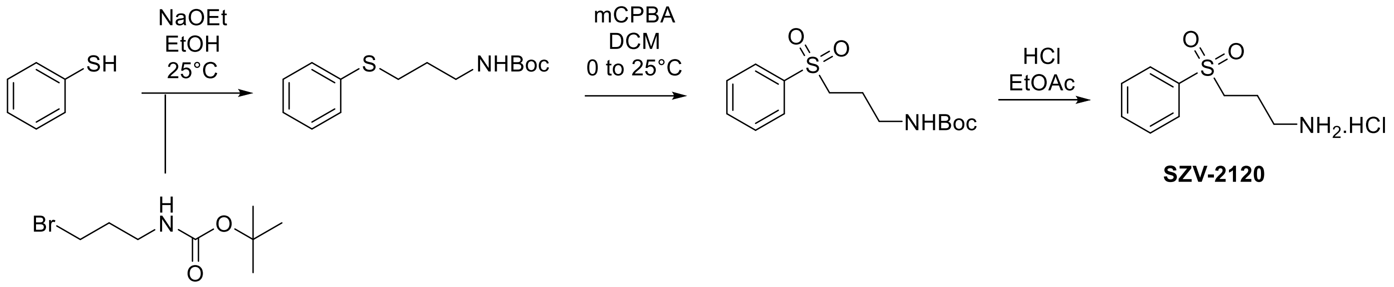

tert-butyl [3-(phenylsulfanyl)propyl]carbamate

A two-neck flask was charged with 40 mL EtOH and sodium (230 mg, 10.00 mmol, 1.0 equiv) was added (stirring till complete dissolution and cooling back to rt). Then thiophenol (1.00 mL, 10.00 mmol, 1.0 equiv) was added, followed by 10 min stirring. Finally tert-butyl (3-bromopropyl)carbamate (2.38 g, 8.00 mmol, 1.0 equiv) was added to the reaction mixture. The reaction was stirred at rt for 16 h (reaction monitored with TLC). The reaction mixture was evaporated to dryness. Water (40 mL) was added and the mixture was extracted with 3×40 mL DCM. The combined organic phases were washed with brine (1×40 mL), dried over MgSO4, filtered and evaporated to dryness. The crude product was purified with column chromatography (CHCl3). Colourless oil (2.50 g, 93%). 1H NMR (CDCl3) δ (ppm): 7.35–7.31 (m, 2H), 7.30–7.25 (m, 2H), 7.20–7.15 (m, 1H), 4.63 (br s, 1H), 3.24 (dd, J = 15.2, 5.9 Hz, 2H), 2.94 (t, J = 7.2 Hz, 2H), 1.81 (quin, J = 7.0 Hz, 2H), 1.43 (s, 9H). 13C NMR (CDCl3) δ (ppm): 156.1, 136.3, 129.4, 129.0, 126.2, 79.5, 39.6, 31.1, 29.5, 28.5.

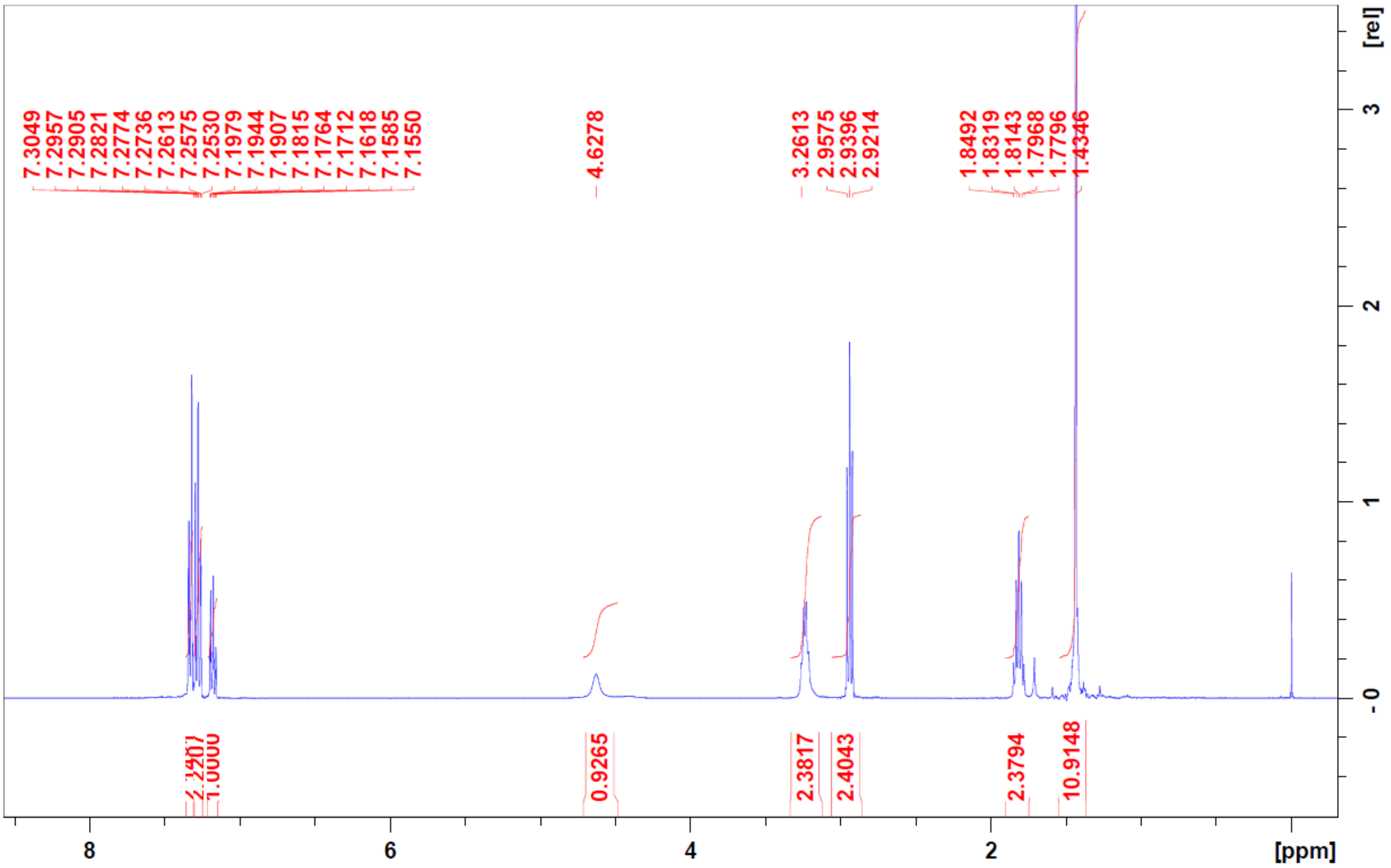

Figure S16. ^{1}H-NMR spectrum of *tert*-butyl [3-(phenylsulfanyl)propyl]carbamate

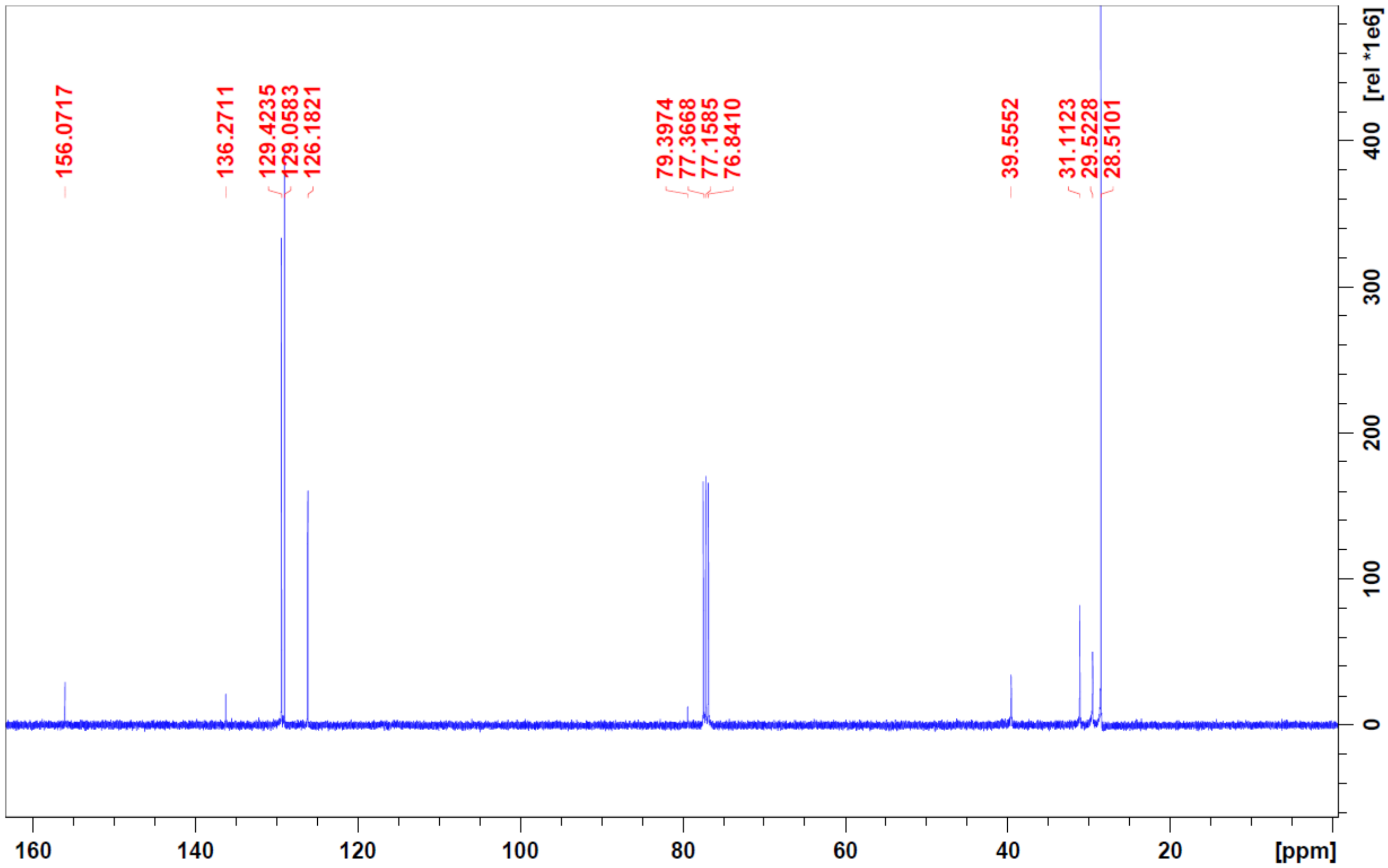

Figure S17. ^{13}C-NMR spectrum of *tert*-butyl [3-(phenylsulfanyl)propyl]carbamate

***tert*-butyl [3-(benzenesulfonyl)propyl]carbamate**

A three-neck flask equipped with a thermometer and a dropping funnel was charged with tert-butyl [3-(phenylsulfanyl)propyl]carbamate (535 mg, 2.00 mmol, 1.0 equiv) and DCM (5 mL). The solution was cooled to 0°C and a solution of m-chloroperbenzoic acid (~70%, 493 mg, 2.00 mmol, 1.0 equiv) in DCM (15 mL) was added dropwise upon cooling. After the addition of the reagent, the reaction mixture was let to warm up to rt and was stirred for 16 h (reaction monitored with TLC). The precipitate was filtered off and washed with 3 × 5 mL DCM. The filtrate was extracted with 1×15 mL sat. Na2SO3, 1×15 mL sat. NaHCO3 and 1 × 15 mL water, dried over MgSO4, filtered and evaporated to dryness. The obtained crude product was purified with column chromatography (EtOAc:CHCl3 5:95). The solid obtained after evaporation of the fractions was dissolved in CHCl3, filtered and evaporated to dryness. White crystals (534 mg, 89%), mp 117.6–118.9°C. 1H NMR (CDCl3) δ (ppm): 7.93–7.89 (m, 2H), 7.70–7.64 (m, 1H), 7.61–7.55 (m, 2H), 4.75 (br s, 1H), 3.23 (dd, J = 14.8, 6.3 Hz, 2H), 3.17–3.11 (m, 2H), 1.97–1.87 (m, 2H), 1.41 (s, 9H). 13C NMR (CDCl3) δ (ppm): 139.0, 134.3, 129.5, 128.1, 79.7, 53.8, 38.9, 28.5, 23.7. Anal. (C14H21NO4S) calcd. C 56.16; H 7.07; N 4.68. Found: C 56.08; H 7.05; N 4.62.

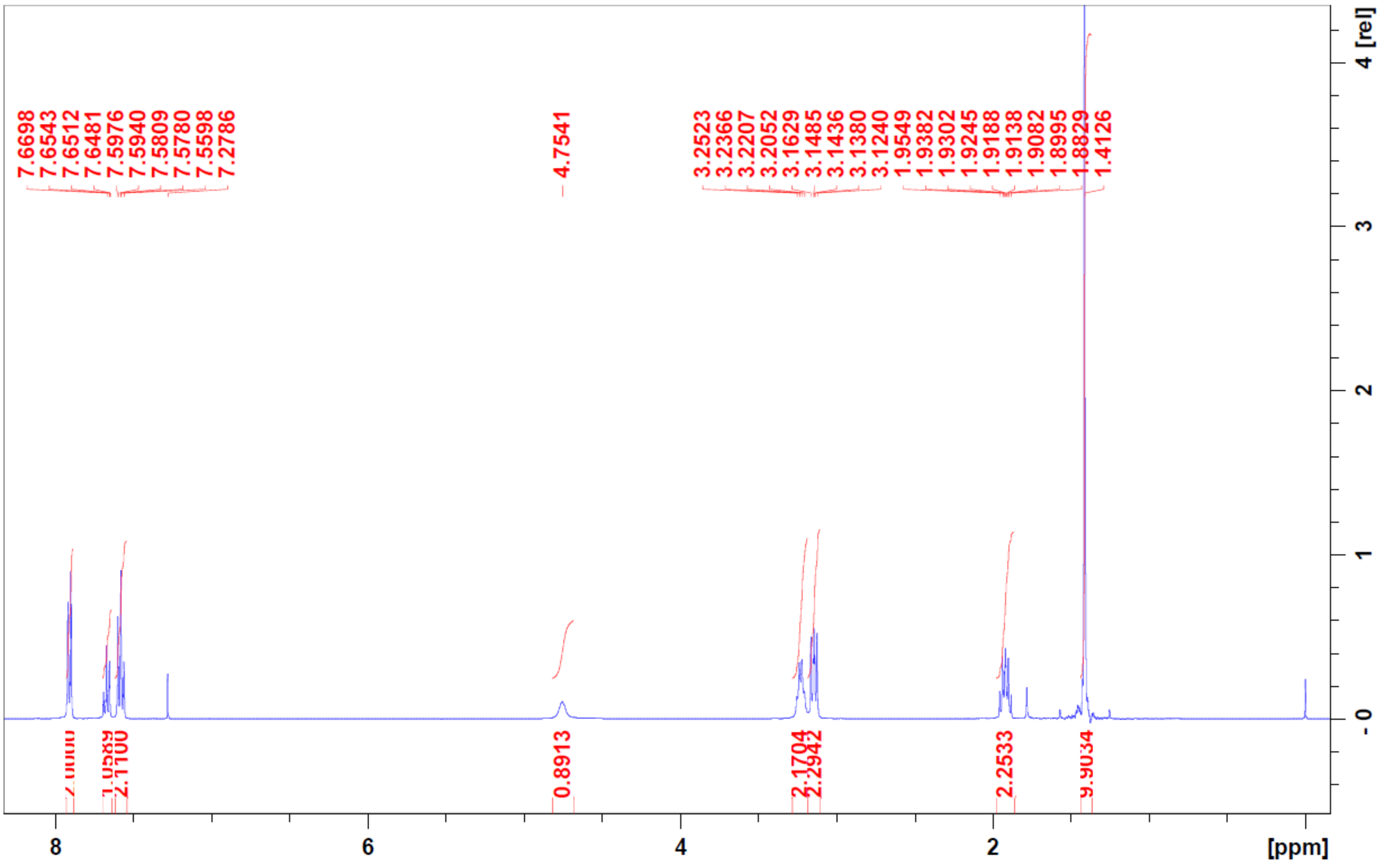

Figure S18. ^{1}H-NMR spectrum of *tert*-butyl [3-(benzenesulfonyl)propyl]carbamate

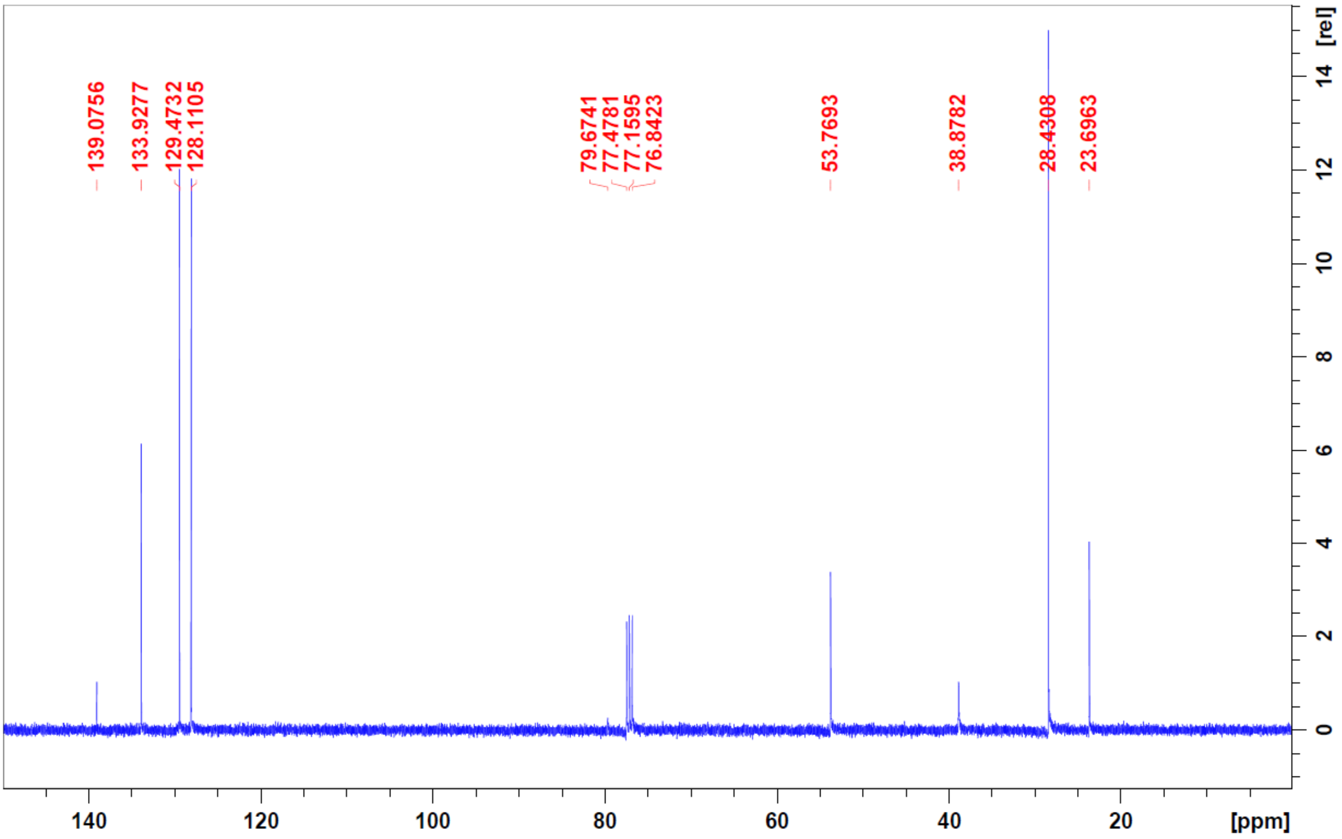

Figure S19. ^{13}C-NMR spectrum of compound 2a

3-(benzenesulfonyl)propan-1-amine hydrochloride salt (SZV-2120)

To a solution of tert-butyl [3-(benzenesulfinyl)propyl]carbamate (300 mg, 1.00 mmol) in EtOAc (5 mL) a solution of HCl in EtOAc (4.37 M, 1 mL) was added dropwise upon cooling (0–5°C). After the addition of the reagent, the solution was let to warm up to rt. After 4 h stirring an additional amount of HCl in EtOAc was added (4.37 M, 1 mL) and the mixture was stirred for a further 4 h at rt (reaction monitored with TLC). The reaction mixture was cooled back to 0–5°C, the precipitated crystals were filtered off, washed with 2×5 mL EtOAc, 2×5 mL diethyl ether, 2×5 mL pentane and dried under vacuum. White crystals (176 mg, 75%), mp 224.0–224.7°C. 1H NMR (DMSO-d6) δ (ppm): 8.10 (br s, 2H), 7.92–7.88 (m, 2H), 7.81–7.75 (m, 1H), 7.72–7.66 (m, 2H), 3.51–3.5 (m, 2H), 2.85 (t, J = 7.4 Hz, 2H), 1.86 (quint, J = 7.5 Hz, 2H). 13C NMR (DMSO-d6) δ (ppm): 138.7, 134.1, 129.6, 127.7, 51.8, 37.2, 20.8. Anal. (C9H14ClNO2S) calcd. C 45.86; H 5.99; N 5.94.

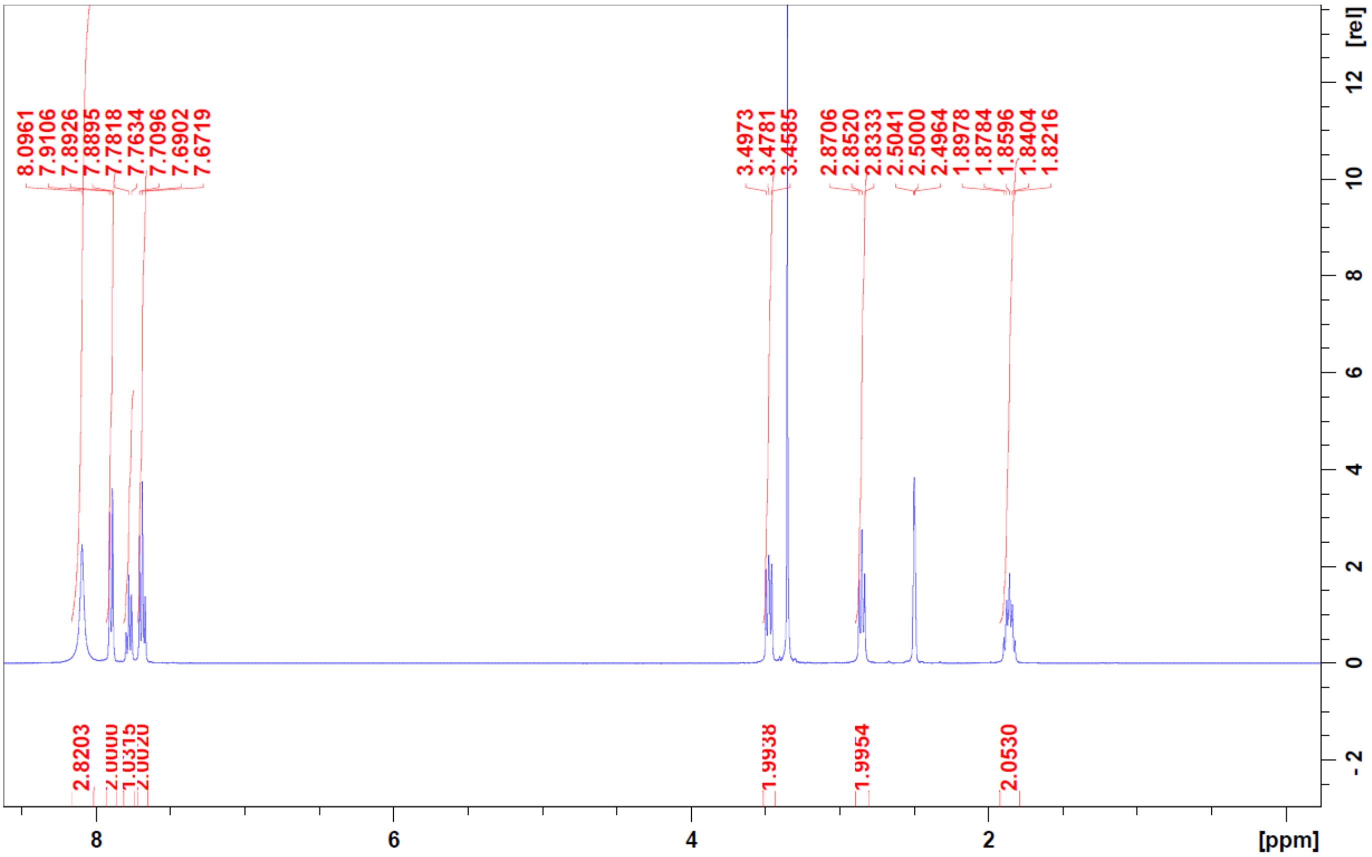

Figure S20. ^{1}H-NMR spectrum of 3-(benzenesulfonyl)propan-1-amine hydrochloride

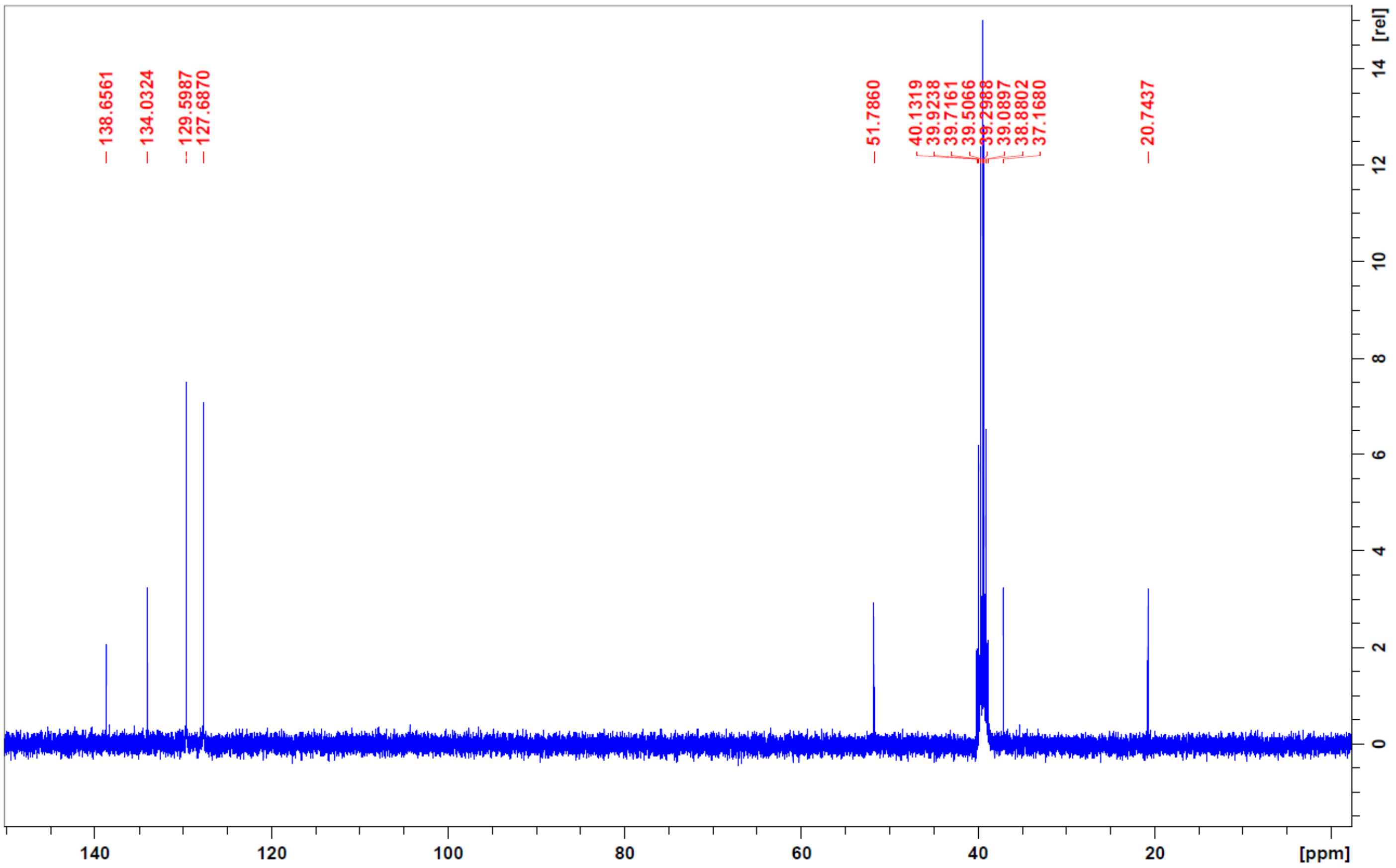

Figure S21. ^{13}C-NMR spectrum of 3-(benzenesulfonyl)propan-1-amine hydrochloride

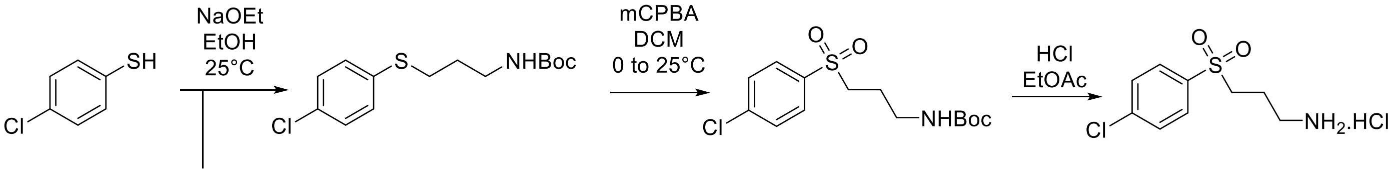

SZV-2121

***tert*-butyl [3-(4-chlorobenzene-1-sulfonyl)propyl]carbamate**

A three-neck flask equipped with a thermometer and a dropping funnel was charged with tert-butyl {3-[(4-chlorophenyl)sulfanyl]propyl}carbamate (905 mg, 3.00 mmol, 1.0 equiv) and DCM (85 mL). The solution was cooled to 0°C and a solution of m-chloroperbenzoic acid (~70%, 739 mg, 3.00 mmol, 1.0 equiv) in DCM (24 mL) was added dropwise upon cooling. After the addition of the reagent, the reaction mixture was let to warm up to rt and was stirred for 18 h (reaction monitored with TLC). The precipitate was filtered off and washed with 3×10 mL DCM. The filtrate was extracted with 1×25 mL sat. Na2SO3, 1×25 mL sat. NaHCO3 and 1×25 mL water, dried over MgSO4, filtered and evaporated to dryness. The obtained crude product was purified with column chromatography (EtOAc:CHCl3 5:95). The solid obtained after evaporation of the fractions was dissolved in CHCl3, filtered and evaporated to dryness. White crystals (890 mg, 89%), mp 115.0–115.9°C. 1H NMR (CDCl3) δ (ppm): 7.87–7.82 (m, 2H), 7.58–7.53 (m, 2H), 4.72 (br s, 1H), 3.23 (q, J = 6.4 Hz, 2H), 3.17–3.11 (m, 2H), 1.96–1.87 (m, 2H), 1.42 (s, 9H). 13C NMR (CDCl3) δ (ppm): 156.0, 140.7, 137.5, 129.8, 129.7, 79.8, 53.9, 38.9, 28.4, 23.7. Anal. (C14H21NO4S) calcd. C 50.37; H 6.04; N 4.20. Found: C 50.54; H 5.59; N 4.15.

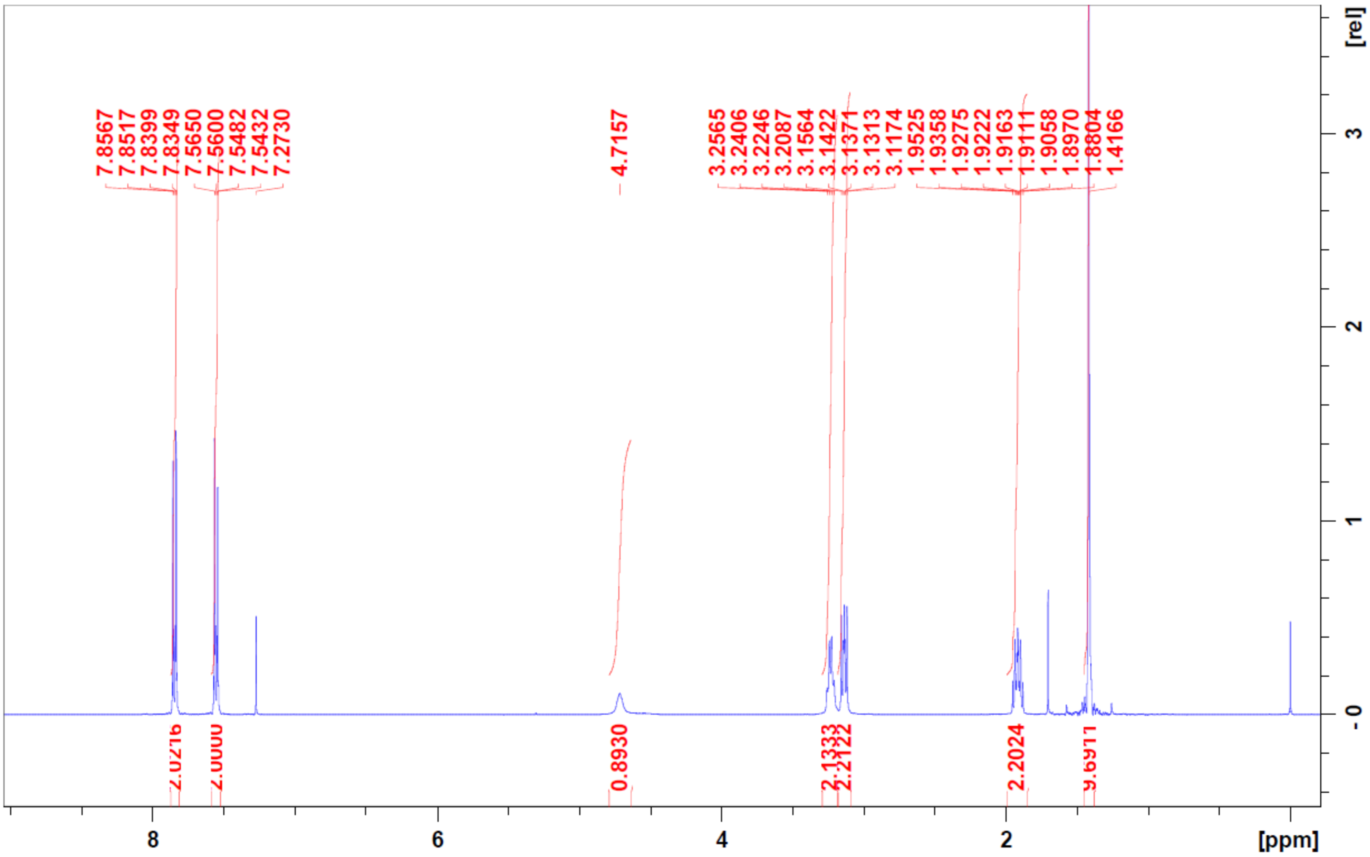

Figure S22. ^{1}H-NMR spectrum of *tert*-butyl [3-(4-chlorobenzene-1-sulfonyl)propyl]carbamate

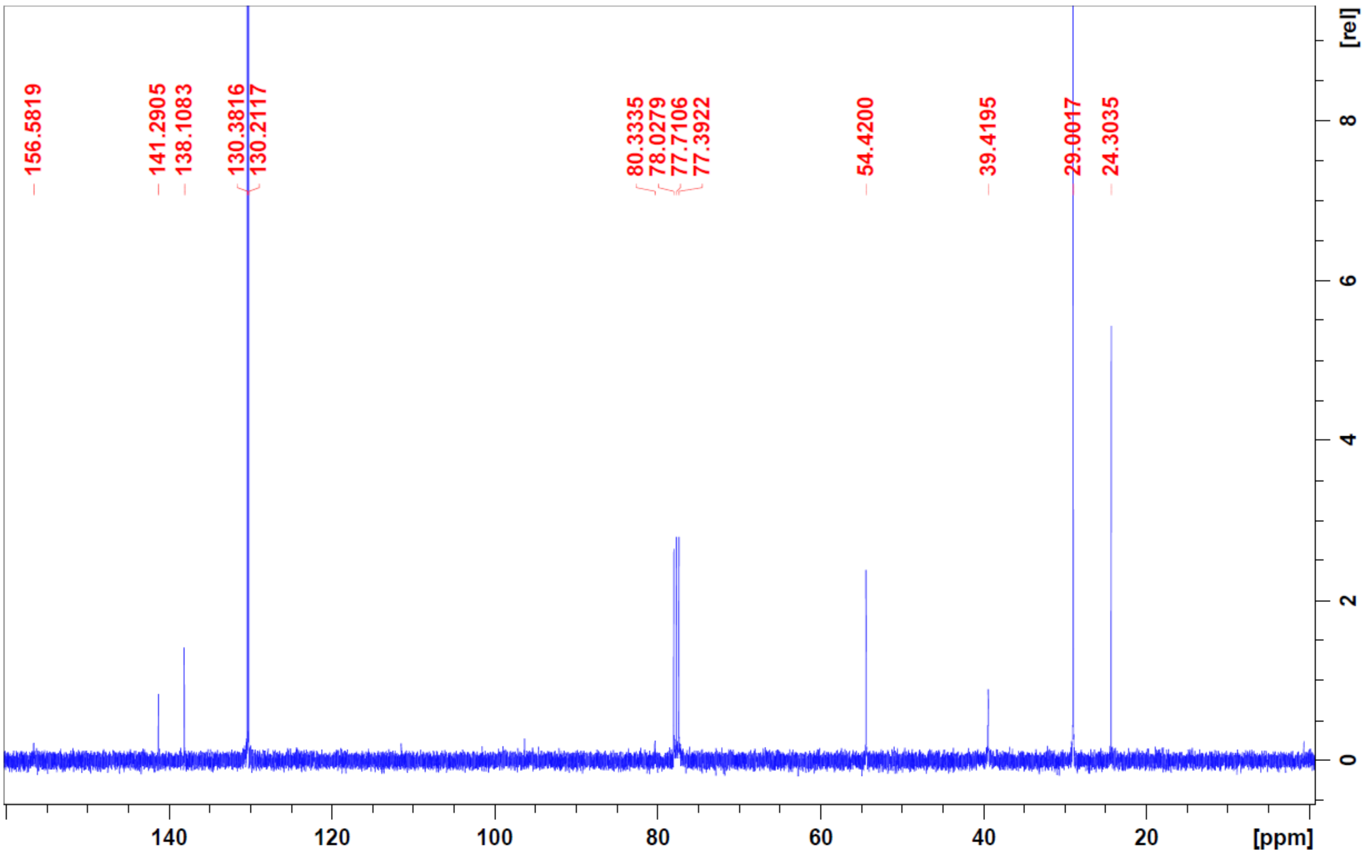

Figure S23. ^{13}C-NMR spectrum of *tert*-butyl [3-(4-chlorobenzene-1-sulfonyl)propyl]carbamate

3-(4-chlorobenzene-1-sulfonyl)propan-1-amine hydrochloride salt (SZV-2121)

To a solution of tert-butyl [3-(4-chlorobenzene-1-sulfonyl)propyl]carbamate (667 mg, 2.00 mmol) in EtOAc (10 mL) a solution of HCl in EtOAc (4.37 M, 2 mL) was added dropwise upon cooling (0–5°C). After the addition of the reagent, the solution was let to warm up to rt. After 4 h stirring an additional amount of HCl in EtOAc was added (4.37 M, 2 mL) and the mixture was stirred for a further 4 h at rt (reaction monitored with TLC). The reaction mixture was cooled back to 0–5°C, the precipitated crystals were filtered off, washed with 2 × 5 mL EtOAc, 2×5 mL diethyl ether, 2 × 5 mL pentane and dried under vacuum, then re-crystallized from EtOH. White crystals (482 mg, 89%), mp 200.7–202.0°C. 1H NMR (DMSO-d6) δ (ppm): 8.17 (br s, 2H), 7.94–7.89 (m, 2H), 7.80–7.75 (m, 2H), 3.56–3.49 (m, 2H), 2.85 (t, J = 7.3 Hz, 2H), 1.86 (quint, J = 7.7 Hz, 2H). 13C NMR (DMSO-d6) δ (ppm): 138.9, 137.2, 129.6, 129.5, 51.6, 36.9, 20.5. Anal. (C9H13Cl2NO2S) calcd. C 40.01; H 4.85; N 5.18. Found: C 39.91; H 4.51; N 5.10.

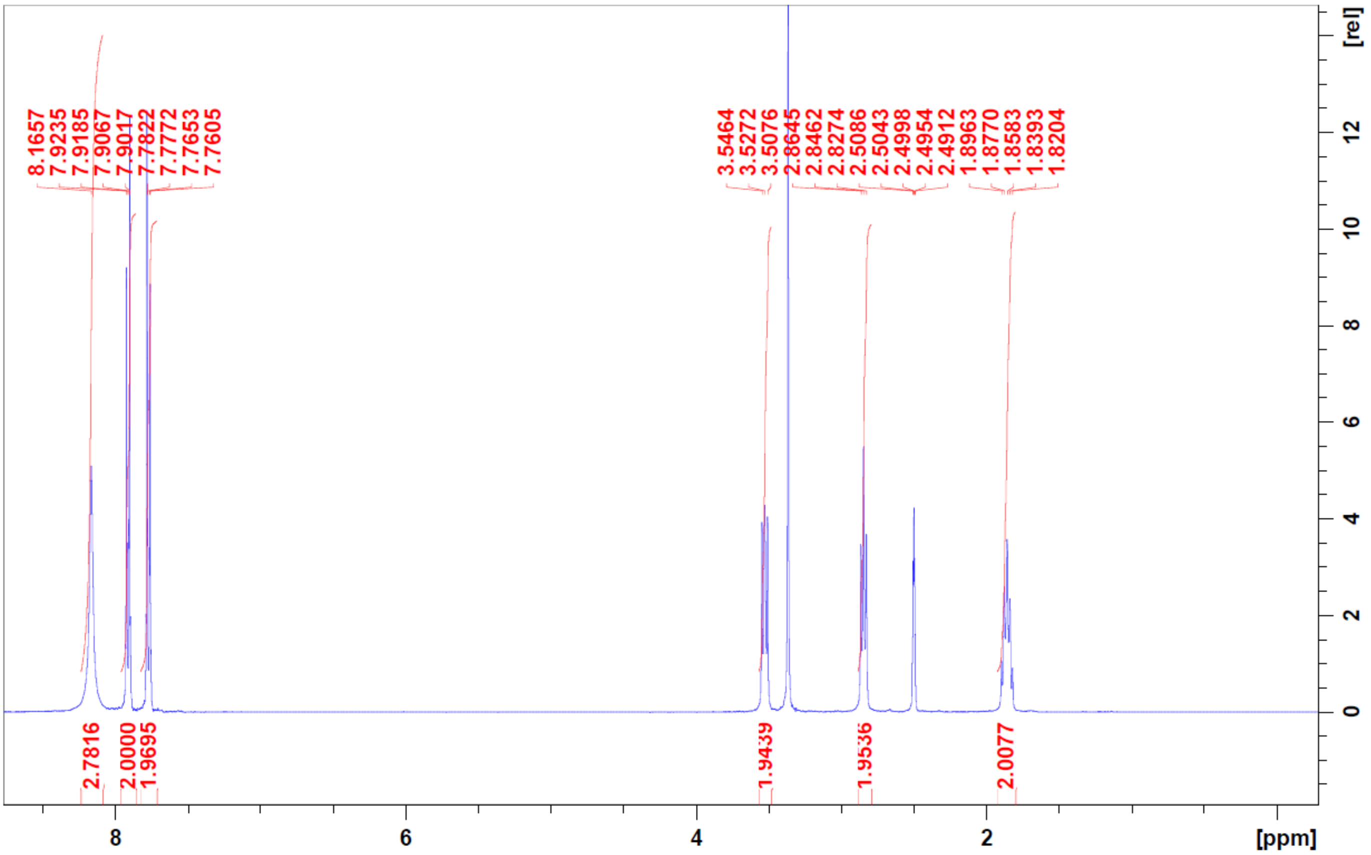

Figure S24. ^{1}H-NMR spectrum of 3-(4-chlorobenzene-1-sulfonyl)propan-1-amine hydrochloride

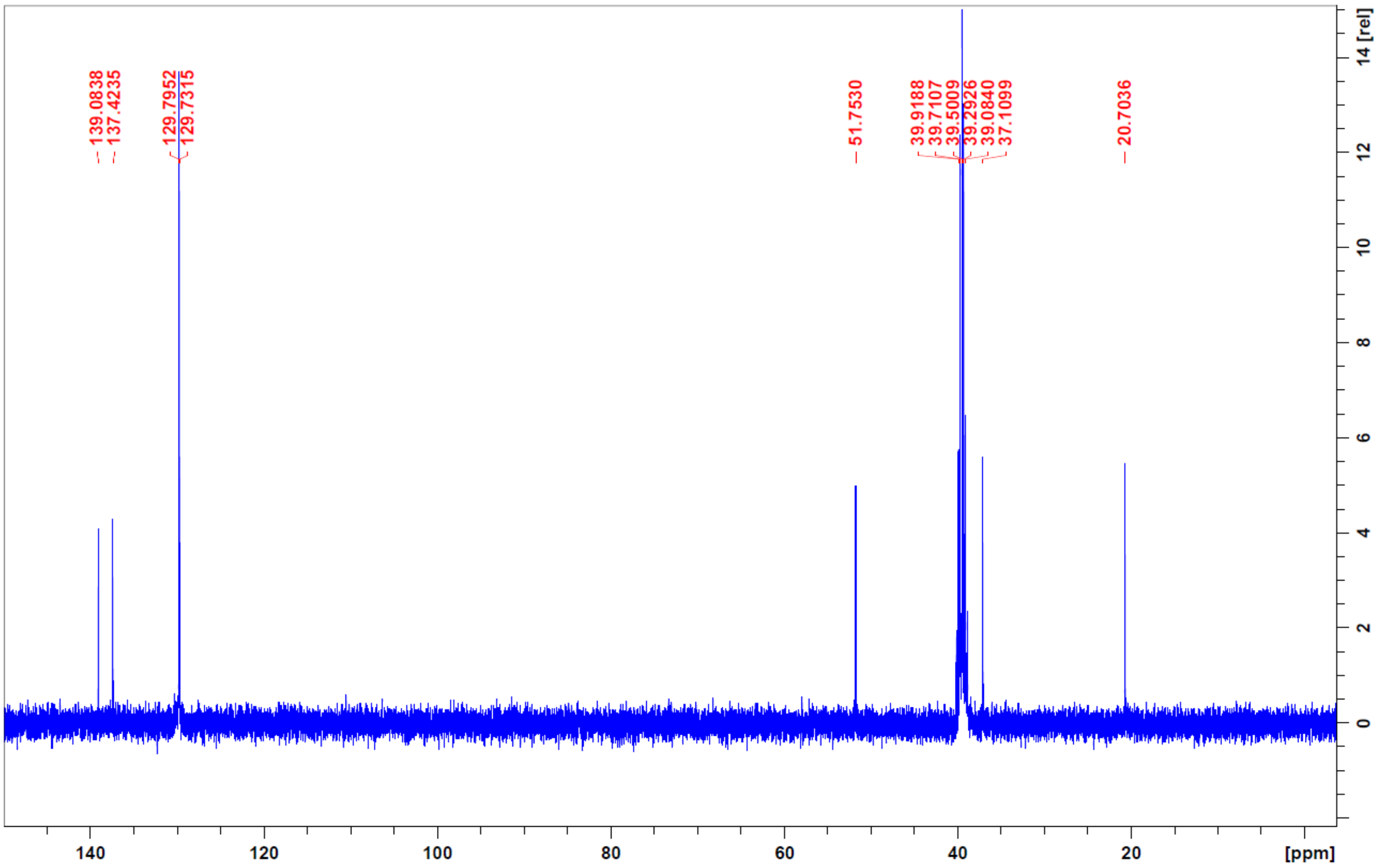

Figure S25. ^{13}C-NMR spectrum of 3-(4-chlorobenzene-1-sulfonyl)propan-1-amine hydrochloride

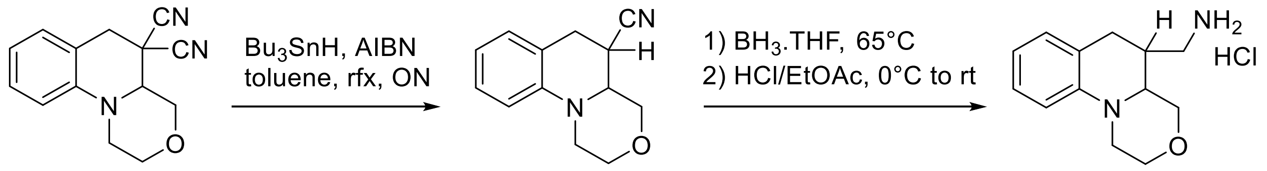

SZV-1398

1-(1,2,4,4a,5,6-hexahydro[1,4]oxazino[4,3-*a*]quinoline-5-yl)methanamine hydrochloride (2×HCl) (SZV-1398) [7].

The mononitrile derivative [8] (2.00 mmol) was dissolved in dry THF (20 mL). Under argon at 0°C BH3.THF (20.00 mmol, 10.0 equiv) was added dropwise. The reaction mixture was heated to reflux for 3 h, then cooled back to 0–5°C. Upon cooling methanol was added dropwise and the mixture was heated to reflux for 30 min. The white precipitate formed was filtered off, the filtrate was evaporated to dryness and the crude product thus obtained was purified with column chromatography (MeOH:EtOAc 60:40). The oil obtained was dissolved in EtOAc and treated with EtOAc/HCl.

White crystals (460 mg, 79%), mp 180°C (dec). 1H NMR (DMSO-d6) δ (ppm): 9.00 (br.s, 3H), 7.10 (t, J = 8.5 Hz, 1H), 7.01 (d, J = 7.3 Hz, 1H), 6.78 (d, J = 8.5 Hz, 1H), 6.72 (t, J = 8.5 Hz, 1H), 3.92 (dm, J = 9.8 Hz, 1H), 3.80 (dd, J = 11.0, 3,1 Hz, 1H), 3.73 – 3.54 (m, 3H), 3.36 (dt, J = 11.0, 3.1 Hz, 1H), 3.04 – 2.61 (m, 5H), 1.93 (m, 1H). 13C NMR (DMSO-d6) δ (ppm): 130.8, 127.7, 123.2, 119.1, 113.6, 105.5, 69.4, 67.3, 58.5, 48.6, 41.7, 37.9, 31.2. Anal. (C13H21Cl2N3) calcd.C 53.80; H 7.29; N 14.48. Found: C 53.70; H 7.20; N 14.38.

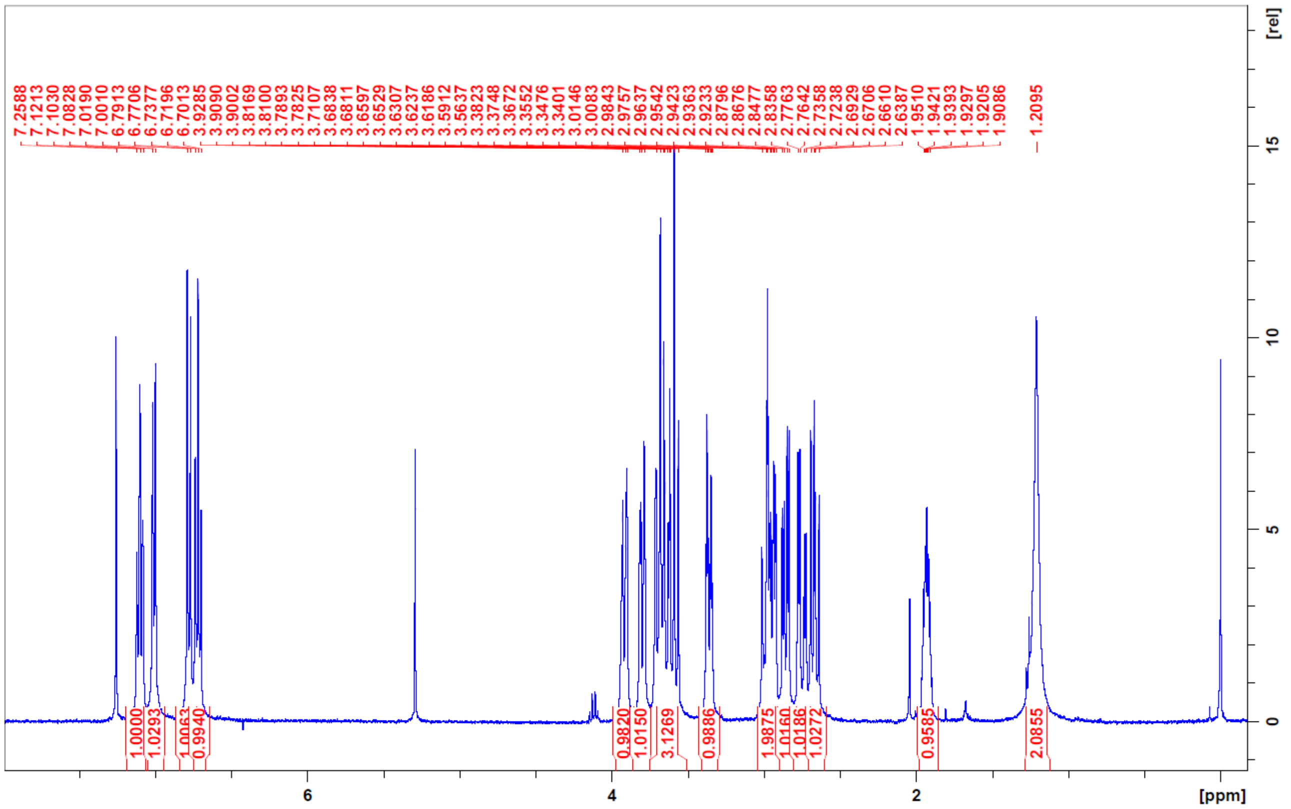

Figure S26. ^{1}H-NMR spectrum of 1-(1,2,4,4a,5,6-hexahydro[1,4]oxazino[4,3-*a*]quinoline-5-yl)methanamine hydrochloride**.**

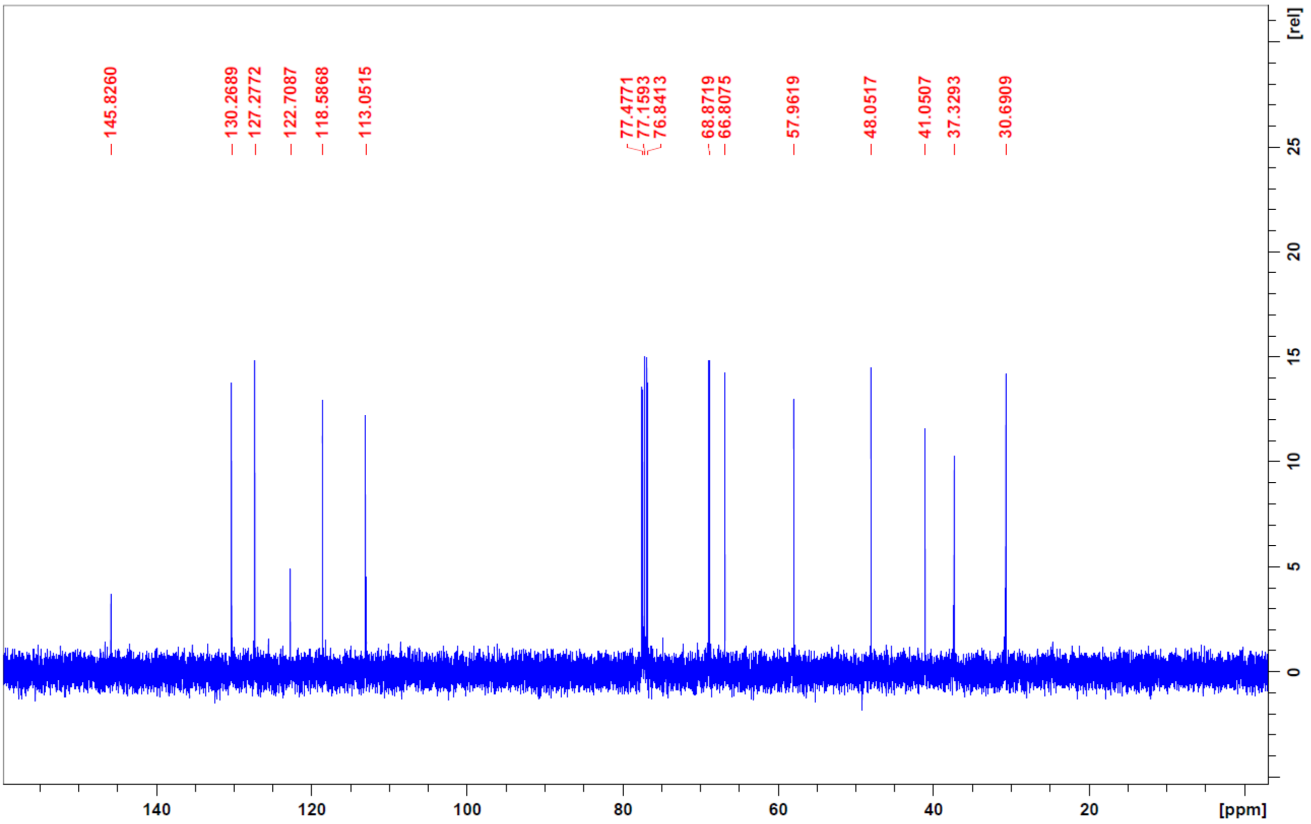

Figure S27. ^{13}C-NMR spectrum of 1-(1,2,4,4a,5,6-hexahydro[1,4]oxazino[4,3-*a*]quinoline-5-yl)methanamine hydrochloride

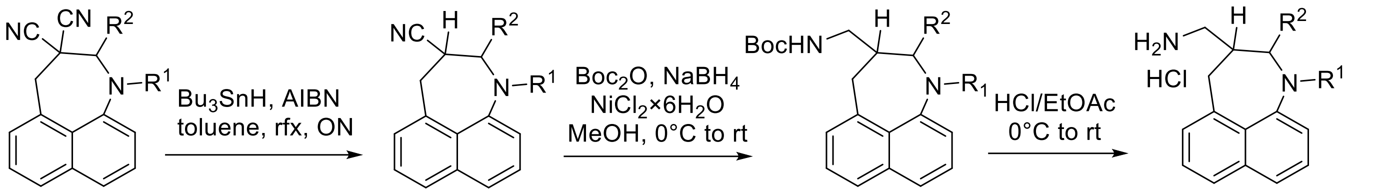

R^1=CH_3, R^2=H **SZV-2142**
R^1+R^2=-$(CH_2)_3$- **SZV-2141**

General procedure for the decyanation [8]

To the solution of the starting germinal dinitrile derivative (5.00 mmol) in dry toluene under argon tributyltin hydride (7.50 mmol) and AIBN (0.50 mmol) were added. The reaction mixture was heated to reflux (110°C) for 24 h. The solvent was distilled off and the crude product was purified with column chromatography (toluene:EtOAc 95:5).

1-methyl-1,2,3,4-tetrahydronaphtho[1,8-*bc*]azepine-3-carbonitrile

White crystals (967 mg, 87%), mp 69.0–69.5°C. 1H NMR (CDCl3) δ (ppm): 7.67 (dd, J = 8.2, 1,2 Hz, 1H), 7.39 – 7.26 (m, 3H), 7.17 (dd, J = 7.1, 0.6 Hz, 1H), 6.80 (dd, J = 7.1, 1.5 Hz, 1H), 3.60 – 3.45 (m, 4H), 3.33 – 3.26 (m, 1H), 3.08 (s, 3H). 13C NMR (CDCl3) δ (ppm): 152.1, 136.6, 134.2, 128.6, 127.2, 126.4, 126.3, 122.4, 121.3, 109.7, 59.3, 41.8, 37.4, 32.3. Anal. (C15H14N2) calcd. C 81.05; H 6.35; N 12.60. Found: C 80.79; H 6.35; N 12.56.

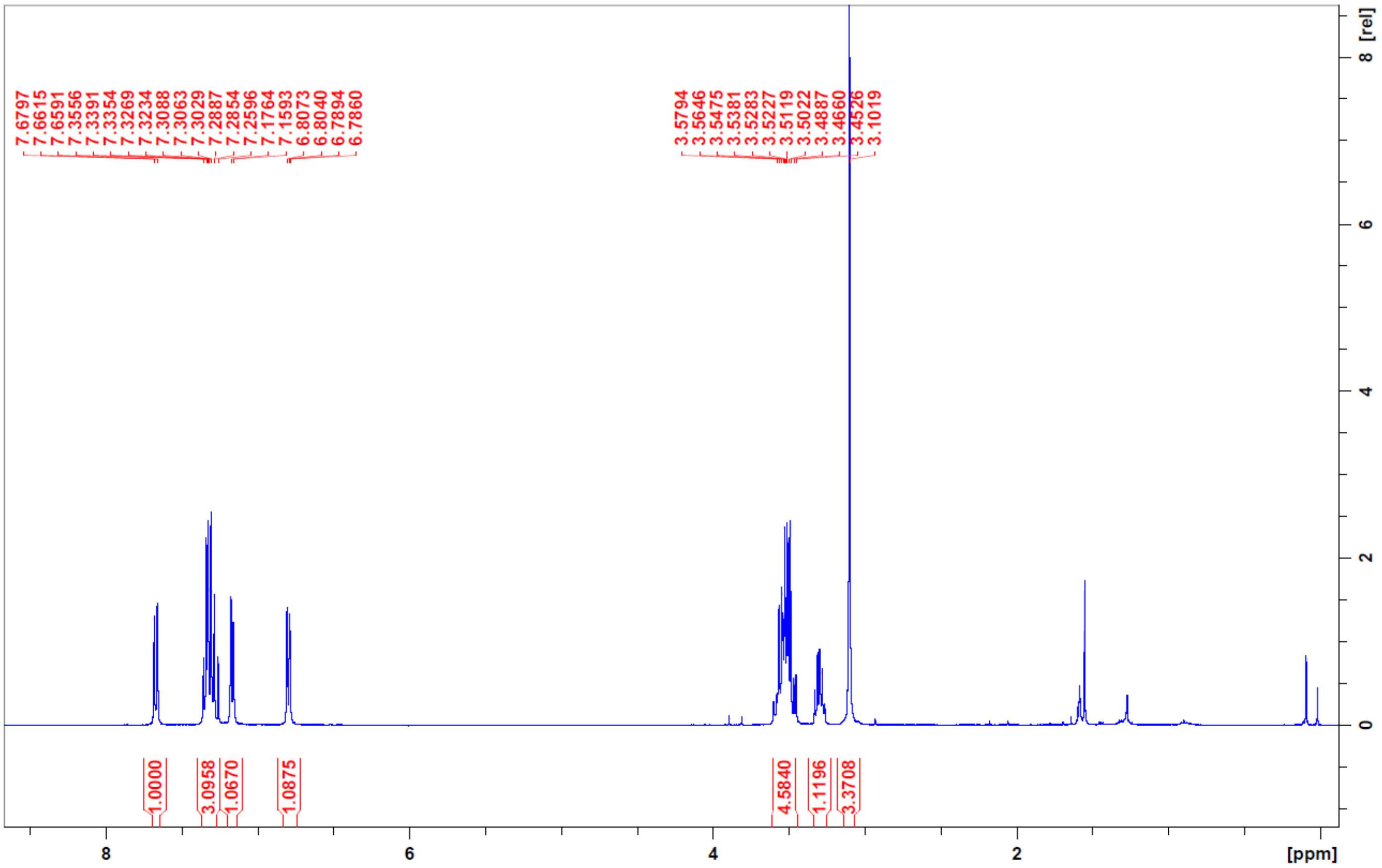

Figure S28. ¹H-NMR spectrum of 1-methyl-1,2,3,4-tetrahydronaphtho[1,8-*bc*]azepine-3-carbonitrile

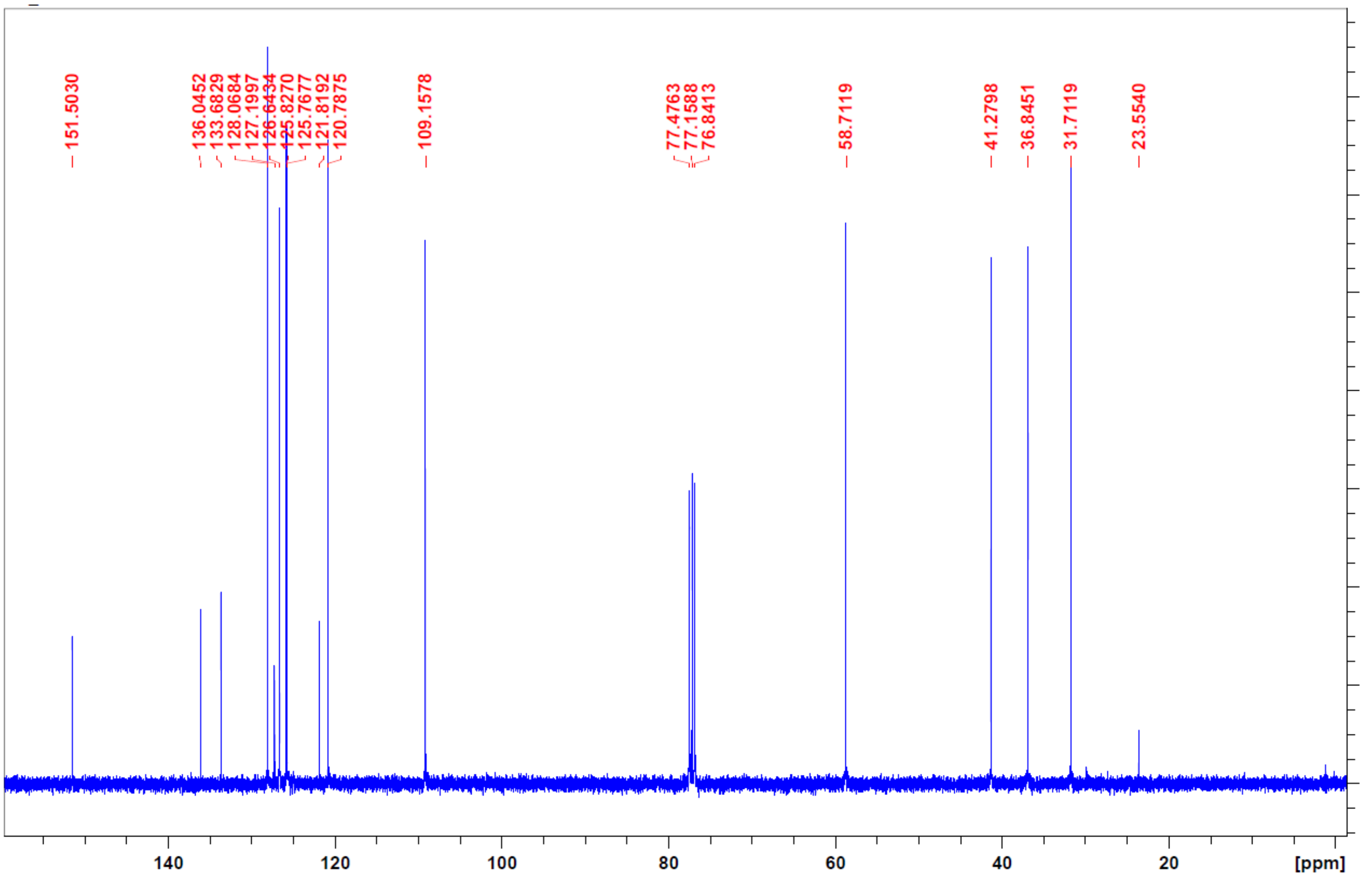

Figure S29. ^{13}C-NMR spectrum of 1-methyl-1,2,3,4-tetrahydronaphtho[1,8-*bc*]azepine-3-carbonitrile

1,2,3a,4,5-hexahydro-4*H*-pyrrolo[1,2-*a*]naphtho[1,8-*bc*]azepine-4-carbonitrile: the two diastereomers were separated with column chromatography (EtOAc:hexane 10:90), DS2 (the 2nd eluting diastereomer) was used for further steps.

DS1 (1st eluting diastereomer): white crystals (509 mg, 41%), mp 112.0–112.5°C. 1H NMR (CDCl3) δ (ppm): 7.55 (dm, J = 8.2 Hz, 1H), 7.22 – 7.16 (m, 3H), 7.01 (d, J = 6.8 Hz, 1H), 6.69 – 6.62 (m, 1H), 3.73 (t, J = 12.7 Hz, 1H), 3.63 (tm, J = 7.1 Hz, 1H), 3.39 (t, J = 7.7 Hz, 1H), 3.26 – 3.15 (m, 1H), 3.06 (dd, J = 12.7, 5.6 Hz, 1H), 3.02 – 2.91 (m, 1H), 2.56 – 2.40 (m, 1H), 2.04 - 1.89 (m, 3H). 13C NMR (CDCl3) δ (ppm): 150.1, 136.3, 134.5, 128.3, 126.7, 126.1, 121.6, 120.4, 108.8, 64.0, 51.0, 39.1, 38.2, 30.6, 24.7. Anal. (C17H16N2) calc. C 82.22; H 6.49; N 11.28. Found: C 82.28; H 6.22; N 11.25.

DS2 (2nd eluting diastereomer): white crystals (484 mg, 39%), mp 116.6–117.7°C. 1H NMR (CDCl3) δ (ppm): 7.66 (d, J = 8.2 Hz, 1H), 7.33 – 7.20 (m, 4H), 6.70 (d, J = 7.1 Hz, 1H), 3.90 (dd, J = 13.8, 7.4 Hz, 1H), 3.70 – 3.65 (m, 1H), 3.40 – 3.37 (m, 1H), 3.16 – 3.10 (m, 1H), 3.04 – 2.95 (m, 2H), 2.13 – 2.04 (m, 3H), 1.96 – 1.87 (m, 1H). 13C NMR (CDCl3) δ (ppm): 149.3, 136.2, 133.7, 128.2, 127.5, 127.3, 126.4, 121.8, 120.7, 108.4, 66.3, 50.9, 39.1, 37.0, 31.4, 24.4. Anal. (C17H16N2) calc. C 82.22; H 6.49; N 11.28. Found: C 82.20; H 6.15; N 11.51.

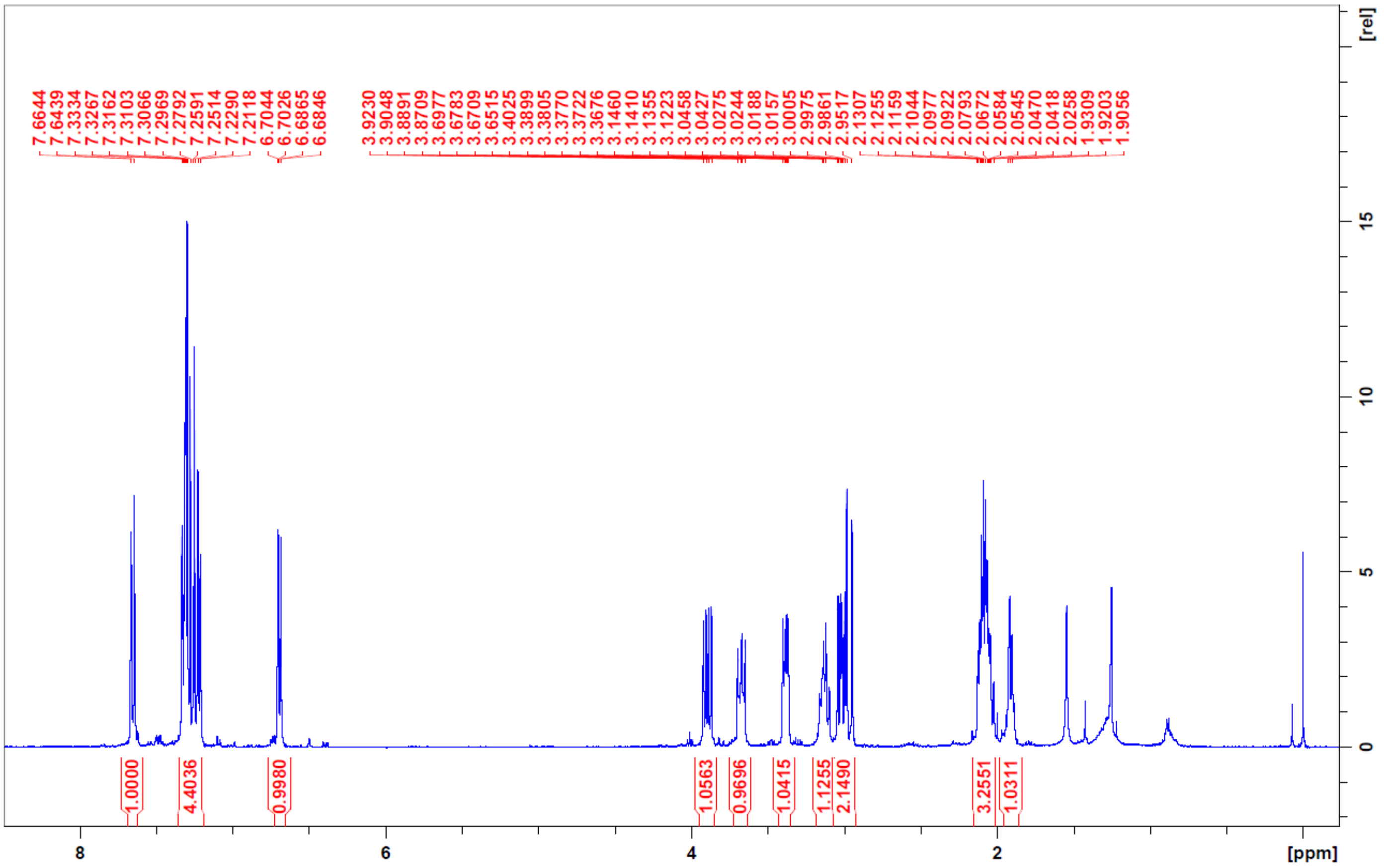

Figure S30. ^{1}H-NMR spectrum of 1,2,3a,4,5-hexahydro-4*H*-pyrrolo[1,2-*a*]naphtho[1,8-*bc*]azepine-4-carbonitrile

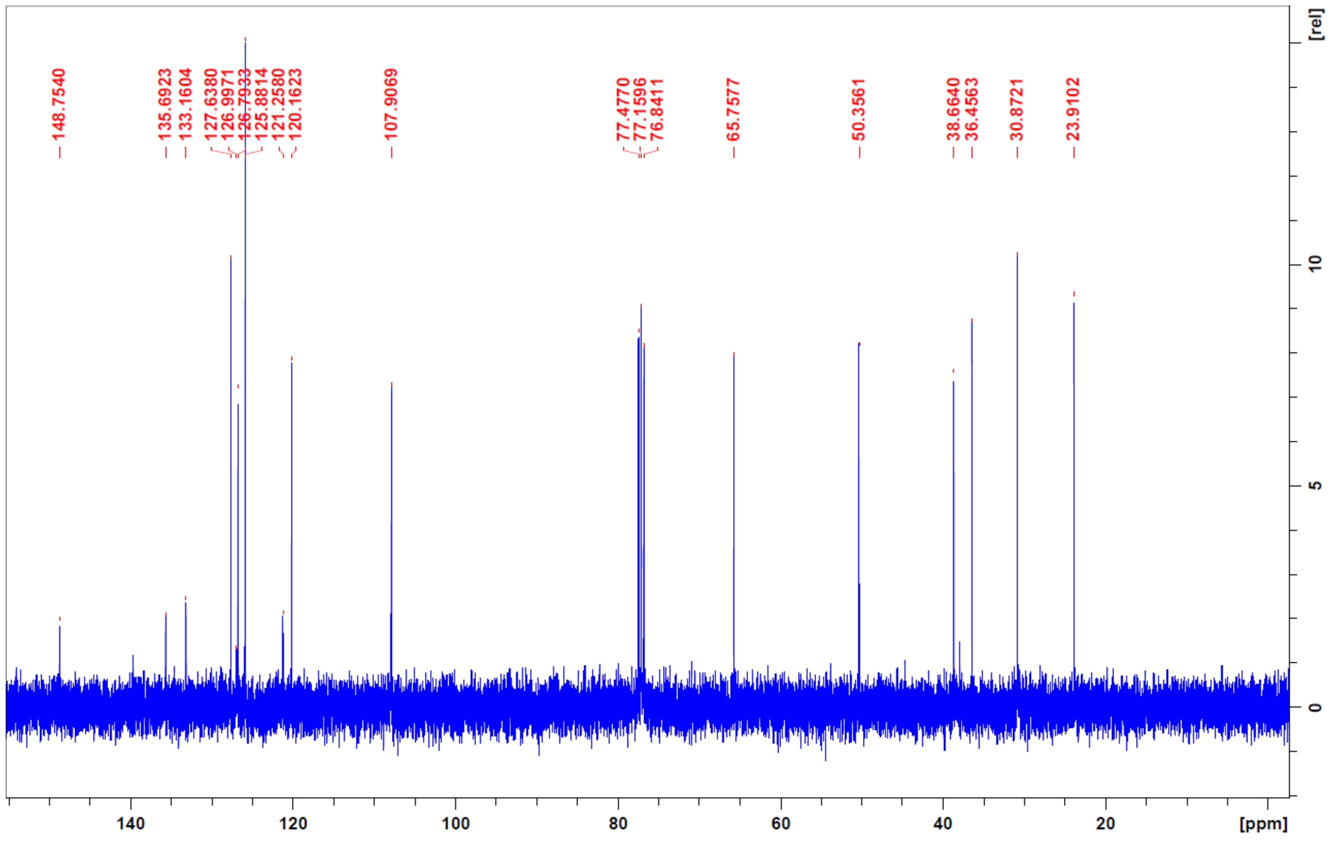

Figure S31. ^{13}C-NMR spectrum of 1,2,3a,4,5-hexahydro-4*H*-pyrrolo[1,2-*a*]naphtho[1,8-*bc*]azepine-4-carbonitrile

General procedure for the reduction [9]

The starting nitrile derivative (2.60 mmol) was dissolved in dry methanol (20 mL). At 0°C Boc2O (5.20 mmol) and NiCl2 × 10 H2O (1.00 mmol) were added to the solution. The NaBH4 (36.70 mmol) was added in small portions under 1 h, keeping the temperature below 5°C. After the addition of the reagent the mixture was let to warm up to rt and was stirred for 15 h. The mixture was cooled back and under 5°C 25% ammonia (15 ml) was added dropwise, then the mixture was stirred at rt for 1 h. The precipitated solid was filtered off and washed with water (2×20 mL). The precipitate was dissolved in EtOAc (50 mL) and extracted with sat. NaHCO3 (3×50 mL). The organic phase was dried over MgSO4, filtered and evaporated to dryness. The crude product was purified with column chromatography (DCM).

***tert*-butyl [(1-methyl-1,2,3,4-tetrahydronaphtho[1,8-*bc*]azepin-3-yl)methyl]carbamate**

White crystals (526 mg, 62%), mp 178.1–178.6°C. 1H NMR (CDCl3) δ (ppm): 7.60 (dd, J = 8.2, 1.1 Hz, 1H), 7.28 – 7.24 (m, 3H), 7.09 (d, J = 7.0 Hz, 1H), 6.73 (d, J = 7.0 Hz, 1H), 4.7 (br s, 1H), 3.29 (d, J = 6.2 Hz, 1H), 3.28 (d, J = 6.5 Hz, 1H), 3.23 – 3.05 (m, 3H), 3.02 (s, 3H), 2.99 (dd, J = 14.2, 5.3 Hz, 1H), 2.40 (m, 1H), 1.48 (s, 9H). 13C NMR (CDCl3) δ (ppm): 156.8, 153.0, 137.5, 136.6, 128.0, 127.4, 126.4, 126.2, 126.1, 120.5, 108.8, 80.1, 60.0, 44.3, 42.6, 41.3, 38.3, 29.1. Anal. (C20H26N2O2) calc. C 73.59; H 8.03; N 8.58. Found: C 73.10; H 8.20; N 8.26.

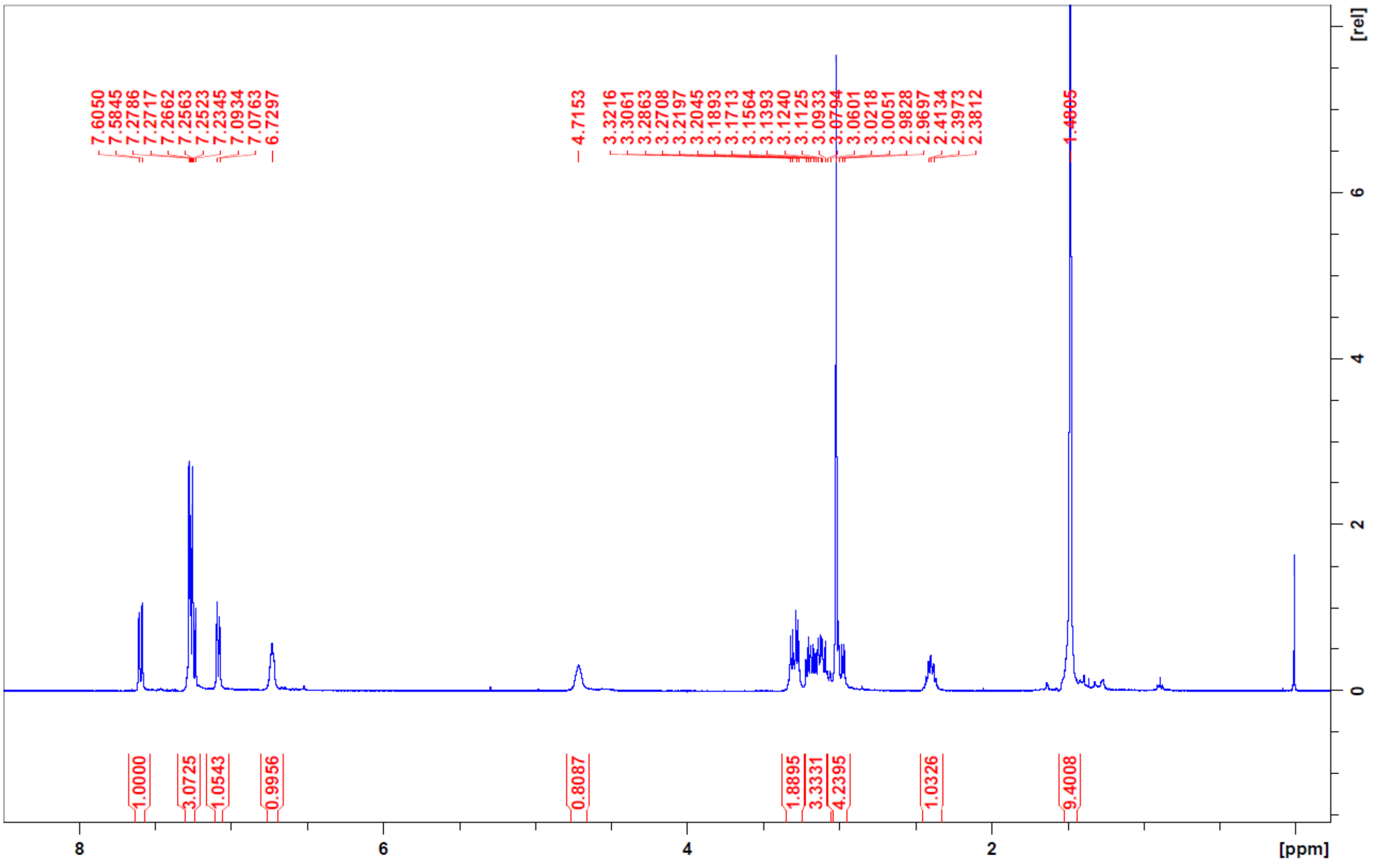

Figure S32. ^{1}H-NMR spectrum of *tert*-butyl [(1-methyl-1,2,3,4-tetrahydronaphtho[1,8-*bc*]azepin-3-yl)methyl]carbamate

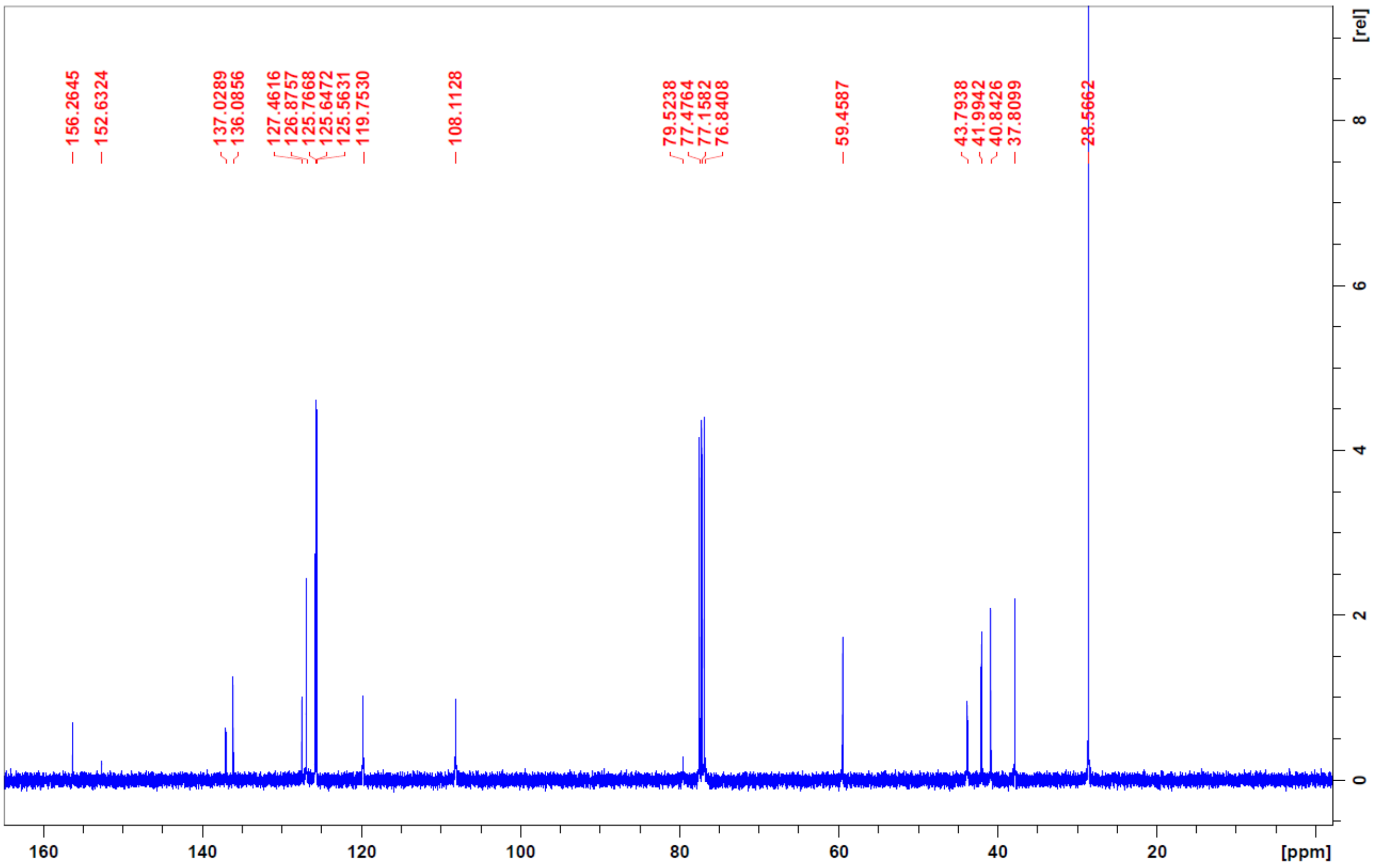

Figure S33. ^{13}C-NMR spectrum of *tert*-butyl [(1-methyl-1,2,3,4-tetrahydronaphtho[1,8-*bc*]azepin-3-yl)methyl]carbamate

***tert*-butyl-[(1,2,3a,4,5-hexahydro-4*H*-pyrrolo[1,2-a]naphtho-[1,8-bc]azepin-3-il)methyl]carbamate**

White crystals (715 mg, 78%), mp 185.2–186.1°C. 1H NMR (CDCl3) δ (ppm): 7.57 (d, J = 8.2 Hz, 1H), 7.31 – 7.20 (m, 3H), 7.06 (d, J = 6.9 Hz, 1H), 6.64 (dd, J = 7.2, 1.6 Hz, 1H), 4.78 (br s, 1H), 3.70 (dd, J = 13.4, 6.5 Hz, 1H), 3.37 (dd, J = 8.4, 6.5 Hz, 1H), 3.18 (m, 1H), 3.06 – 2.99 (m, 3H), 2.65 (d, J = 13.5 Hz, 1H), 2.12 (m, 1H), 2.10 – 2.01 (m, 2H), 2.00 – 1.86 (m, 2H), 1.48 (s, 9H, s). 13C NMR (CDCl3) δ (ppm): 156.7, 150.5, 136.6, 136.3, 127.8, 127.1, 126.6, 126.4, 126.1, 119.4, 107.7, 80.1, 66.8, 51.0, 47.6, 43.0, 37.1, 31.5, 29.1, 25.0. Anal. (C22H28N2O2) calc. C 74.97; H 8.01; N 7.95; O 9.08. Found C 75.78; H 8.23; N 7.87.

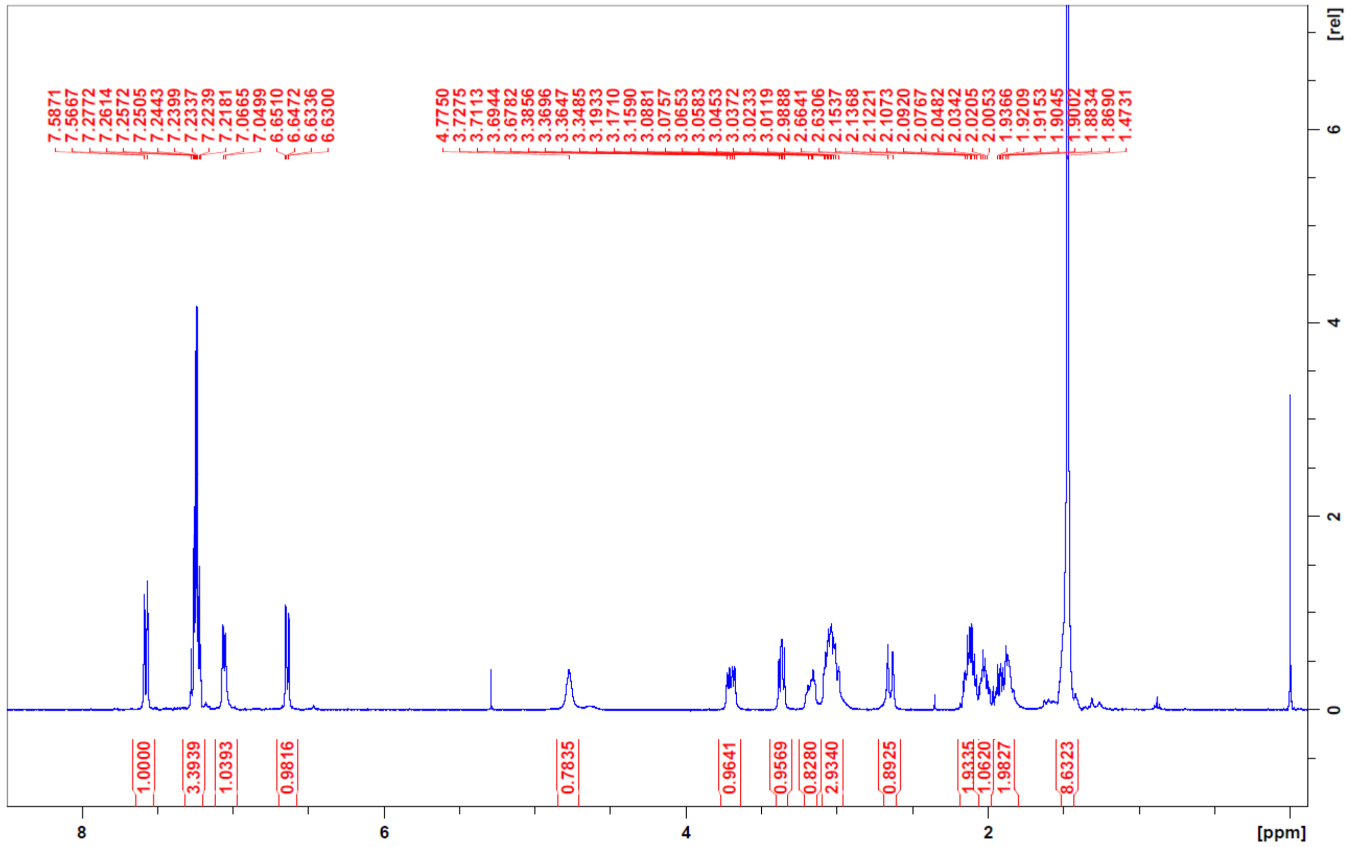

Figure S34. ^{1}H-NMR spectrum of *tert*-butyl-[(1,2,3a,4,5-hexahydro-4*H*-pyrrolo[1,2-a]naphtho-[1,8-bc]azepin-3-il)methyl]carbamate

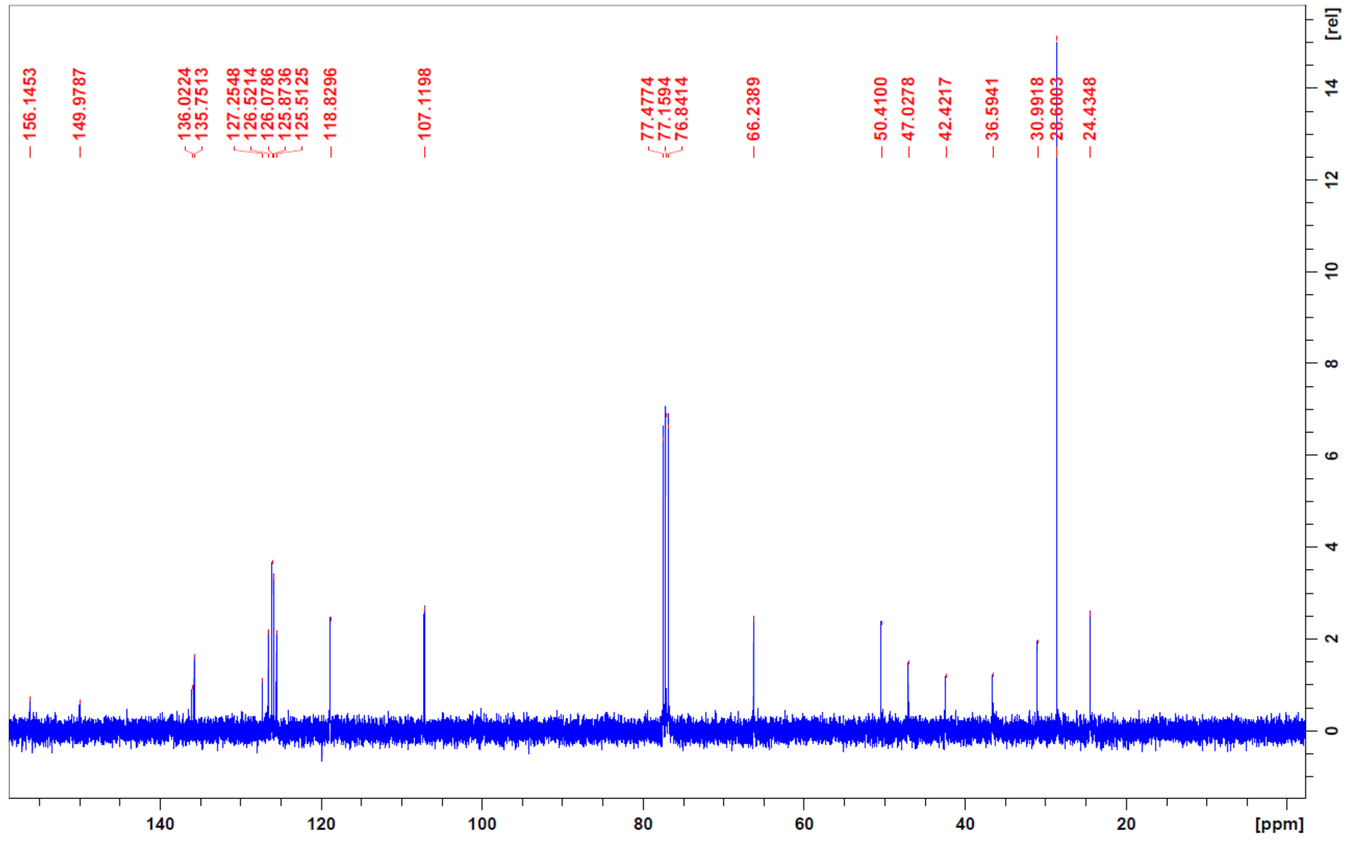

Figure S35. ^{13}C-NMR spectrum of *tert*-butyl-[(1,2,3a,4,5-hexahydro-4*H*-pyrrolo[1,2-a]naphtho-[1,8-bc]azepin-3-il)methyl]carbamate

General procedure for deprotection and salt formation [7]

The Boc amines (400 mg) were dissolved in EtOAc (20 mL). Upon cooling EtOAc/HCl (0.6 M, 10 mL) was added dropwise and the mixture was stirred at rt overnight. The white precipitate was allowed to settle and the EtOAc/HCl was decanted. The precipitate was washed with EtOAc and hexane, filtered and dried under vacuum.

1-(1-methyl-1,2,3,4-tetrahydronaphtho[1,8-*bc*]azepin-3-yl)methanamine hydrochloride (2×HCl) (SZV-2142)

White crystals (292 mg, 80%), mp 150°C (dec). 1H NMR (DMSO-d6) δ (ppm): 7.59 (d, J = 8.2 Hz, 1H), 7.31 – 7.20 (m, 3H), 7.09 (d, J = 6.9 Hz, 1H), 6.69 (dd, J = 7.2, 1.6 Hz, 1H), 3.31 (dd, J = 14.1, 6.3 Hz, 1H), 3.23 (dd, J = 13.0, 6.0 Hz, 1H), 3.00 (m, 5H), 2.85 (dd, J = 12.3, 7.1 Hz, 1H), 2.77 (dd, J = 12.3, 7.3 Hz, 1H), 2.28 (m, 1H), 1.38 (br s, 2H). 13C NMR (DMSO-d6) δ (ppm): 153.4, 138.2, 136.7, 128.1, 127.2, 126.1, 126.1, 126.1, 120.1, 108.4, 60.5, 46.1, 44.3, 42.6, 38.6. Anal. (C15H20Cl2N2) calc. C 60.21; H 6.74; N 9.36. Found: C 60.04; H 6.42; N 9.04.

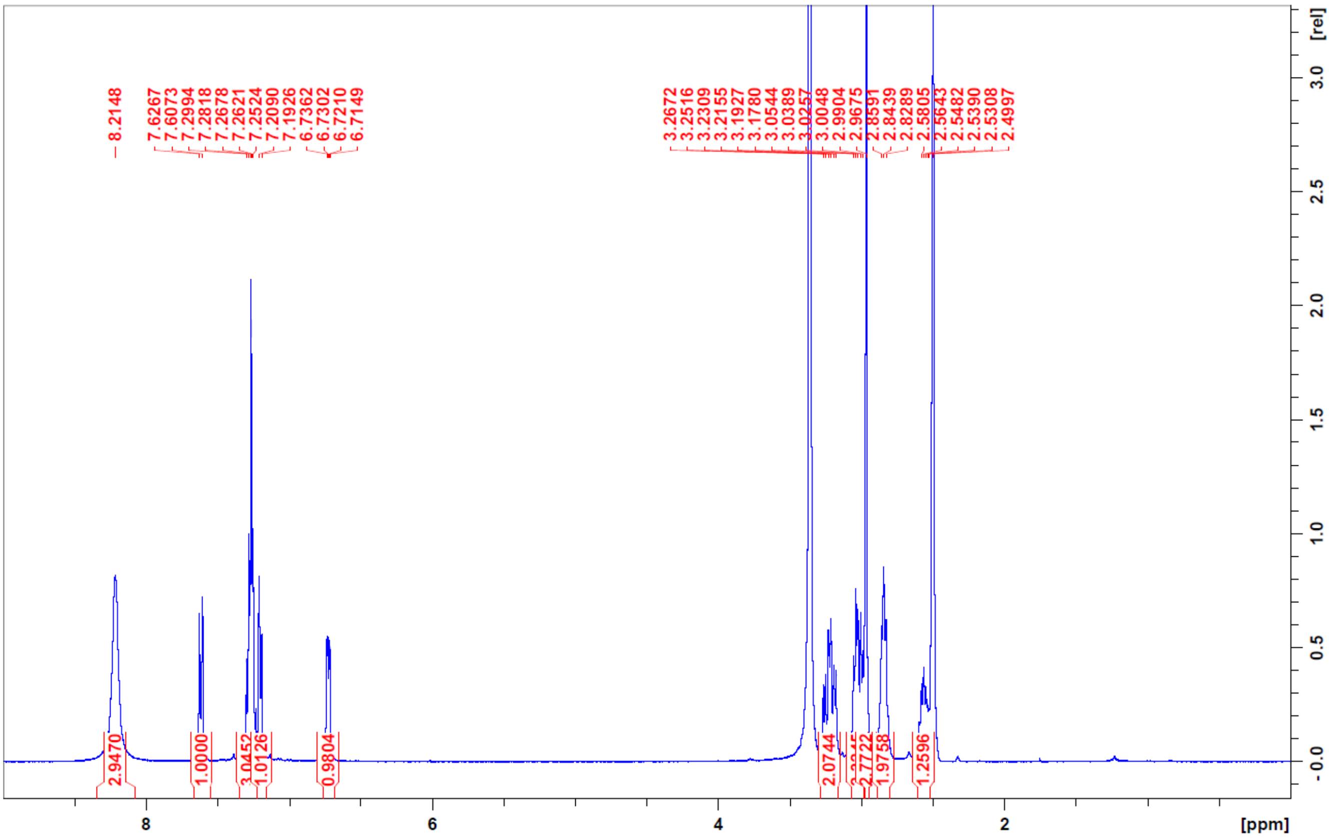

Figure S36. ^{1}H-NMR spectrum of 1-(1-methyl-1,2,3,4-tetrahydronaphtho[1,8-*bc*]azepin-3-yl)methanamine hydrochloride

1-(1,2,3a,4,5-hexahydro-4*H*-pyrrolo[1,2-*a*]naphth[1,8-*bc*]azepin-3-yl)methanamine hydrochloride (SZV-2141)

White crystals(298 mg, 81%, mp 170°C (dec). 1H NMR (DMSO-d6) δ (ppm): 8.35 (br s, 3H), 7.59 (d, J = 8.2 Hz, 1H), 7.37 (d, J = 6.5 Hz, 1H), 7.30 – 7.22 (m, 3H), 6.65 (t, J = 4.4 Hz, 1H), 3.49 (dd, J = 13.5, 6.2 Hz, 1H), 3.31 (t, J = 4.8 Hz, 1H), 3.04 – 2.95 (m, 2H), 2.85 (dd, J = 10.0, 8.3 Hz, 1H), 2.67 (m, 2H), 2.24 (m, 1H), 1.99 (m, 2H), 1.91 – 1.79 (m, 2H). 13C NMR (DMSO-d6) δ (ppm): 149.4, 135.2, 134.9, 126.7, 126.3, 126.2, 125.8, 125.3, 118.5, 107.3, 65.8, 50.1, 44.1, 34.2, 30.1, 23.7. Anal. (C17H21ClN2) calc. C 70.70; H 7.33; N 9.70. Found: C 70.35; H 7.03; N 9.29.

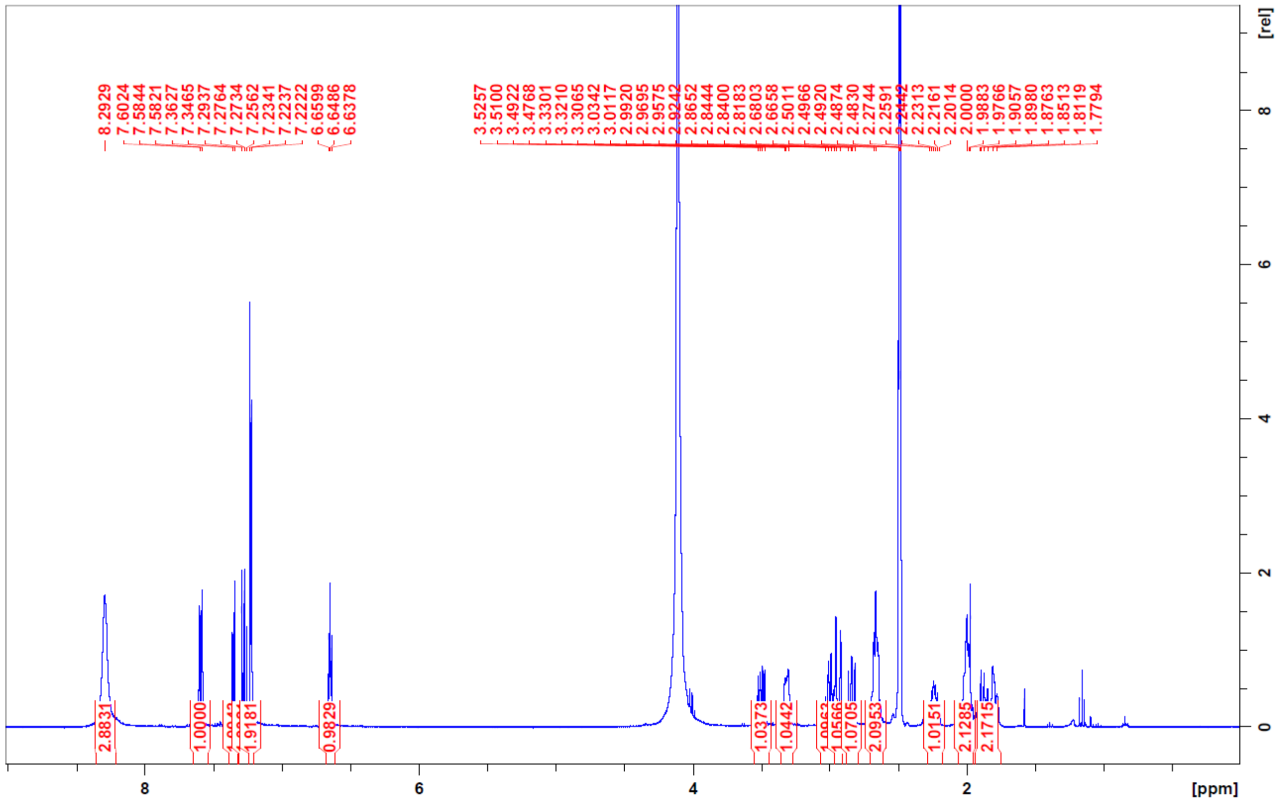

Figure S37. ^{1}H-NMR spectrum of 1-(1,2,3a,4,5-hexahydro-4*H*-pyrrolo[1,2-*a*]naphth[1,8-*bc*]azepin-3-yl)methanamine hydrochloride

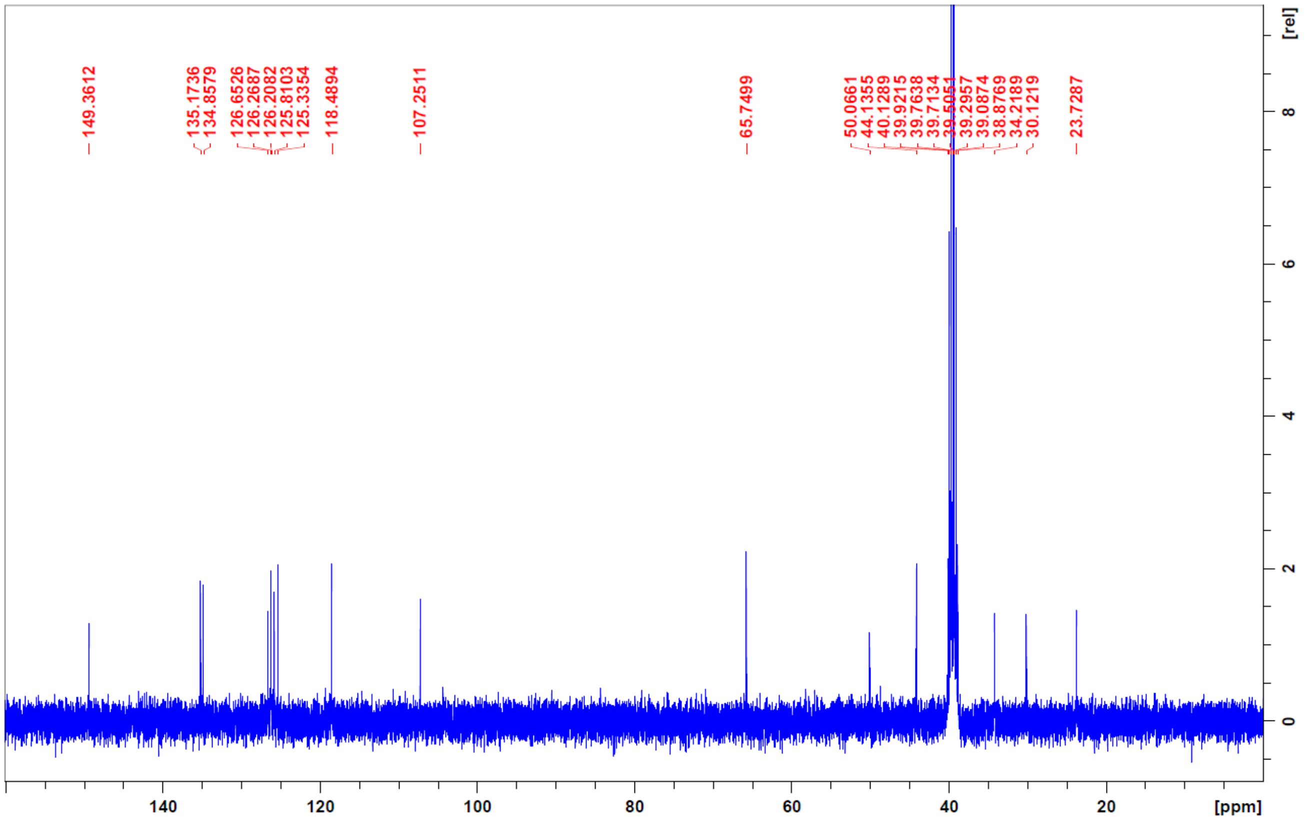

Figure S38. ^{13}C-NMR spectrum of 1-(1,2,3a,4,5-hexahydro-4*H*-pyrrolo[1,2-*a*]naphth[1,8-*bc*]azepin-3-yl)methanamine hydrochloride

References

1. Gallardo-Godoy, A.; Hernandez, M.; Sanz, E.; Unzeta, M. Synthesis of 4-methyl-thio-phenyl-propylamine and the evaluation of its interaction with different amine oxidases. *Bioorg Med Chem* **2004,** 12, 273–279. [doi 10.1016/j.bmc.2003.10.007]
2. Mercader, J.; Iffiu-Soltesz, Z.; Brenachot, X.; Foldi, A.; Dunkel, P.; Balogh, B.; Attané, C.; Valet, P.; Matyus, P.; Carpéné, C. SSAO substrates exhibiting insulin-like effects in adipocytes as a promising treatment option for metabolic disorders. *Future Med Chem* **2010,** 2, 1735–1749. [doi 10.4155/fmc.10.260]
3. Mátyus, P.; Magyar, K.; Pihlavista, M.; Gyires, K.; Haider, N.; Wang, Y.; Woda, P.; Dunkel, P.; Toth-Sarudy, E.; Turos, G. Compounds for inhibiting semicarbazide-sensitive amine oxidase (ssao) / vascular adhesion protein-1 (vap-1) and uses thereof for treatment and prevention of diseases. *PCT Int. Appl. WO2010029379.* **2010,** Available online: URL https://patents.google.com/patent/WO2010029379A1/en,
4. Wang, E. Y.; Gao, H.; Salter-Cid, L.; Zhang, J.; Huang, L.; Podar, E. M.; Miller, A.; Zhao, J.; O'Rourke, A.; Linnik, M. D. Design, synthesis, and biological evaluation of semicarbazide-sensitive amine oxidase (SSAO) inhibitors with anti-inflammatory activity. *J Med Chem* **2006,** 49, 2166–2173. [doi 10.1021/jm050538l]
5. Li, C.; Song, S.; Li, Y.; Xu, C.; Luo, Q.; Guo, Y.; Wang, X. Selective hydroboration of unsaturated bonds by an easily accessible heterotopic cobalt catalyst. *Nat Commun* **2021,** 12, 3813. [doi 10.1038/s41467-021-24117-5]
6. Renard, M.; Ghosez, L. A convergent asymmetric synthesis of γ-butenolides. *Tetrahedron Letters* **1999,** 40, 6237–6240. [doi https://doi.org/10.1016/S0040-4039(99)01242-3]
7. Földi, Á. A. New application of tert-amino effect: synthesis of naphthazepine and naphthazonine ring systems. PhD Thesis, *Semmelweis University, Budapest (Hungary)* **2011**,
8. Gerlach, U. Synthesis of tricyclic cyano-substituted tetrahydroquinolines by radical decyanation of geminal dinitriles. *Tetrahedron Lett* **1995,** 36, [doi doi:10.1016/0040-4039(95)01006-4]
9. Caddick, S.; Judd, D. B.; De K Lewis, A. K.; Reich, M. T.; Williams, M. R. V. A generic approach for the catalytic reduction of nitriles. *Tetrahedron* **2003,** 59, 5417–5423.
